# Asymptotic State Transformations of Continuous Variable Resources

**DOI:** 10.1007/s00220-022-04523-6

**Published:** 2022-12-02

**Authors:** Giovanni Ferrari, Ludovico Lami, Thomas Theurer, Martin B. Plenio

**Affiliations:** 1 Dipartimento di Fisica e Astronomia Galileo Galilei, Università degli studi di Padova, Via Marzolo 8, 35131 Padua, Italy; 2 Institut für Theoretische Physik und IQST, Universität Ulm, Albert-Einstein-Allee 11, 89069 Ulm, Germany

## Abstract

We study asymptotic state transformations in continuous variable quantum resource theories. In particular, we prove that monotones displaying lower semicontinuity and strong superadditivity can be used to bound asymptotic transformation rates in these settings. This removes the need for asymptotic continuity, which cannot be defined in the traditional sense for infinite-dimensional systems. We consider three applications, to the resource theories of (I) optical nonclassicality, (II) entanglement, and (III) quantum thermodynamics. In cases (II) and (III), the employed monotones are the (infinite-dimensional) squashed entanglement and the free energy, respectively. For case (I), we consider the measured relative entropy of nonclassicality and prove it to be lower semicontinuous and strongly superadditive. One of our main technical contributions, and a key tool to establish these results, is a handy variational expression for the measured relative entropy of nonclassicality. Our technique then yields computable upper bounds on asymptotic transformation rates, including those achievable under linear optical elements. We also prove a number of results which guarantee that the measured relative entropy of nonclassicality is bounded on any physically meaningful state and easily computable for some classes of states of interest, e.g., Fock diagonal states. We conclude by applying our findings to the problem of cat state manipulation and noisy Fock state purification.

## Introduction

In recent years, the paradigm of quantum resource theories has established itself as the main framework to analyze and assess the operational usefulness of quantum resources [1–3]. The general setting involves two sets of objects that are considered easily accessible: free states and free operations. Once these have been identified, the resource content of a state is determined by its transformation properties under free operations [3, Section V]. In the long-established tradition of classical [4, 5] as well as quantum [6–8] information theory, in this work we consider ultimate limitations on those transformation properties, and thus look at the asymptotic setting. Namely, we study free approximate conversion of a large number of copies of the initial state $\rho$ into as many copies of the target state $\sigma$ as possible, under the constraint that the approximation error vanishes asymptotically. The resulting transformation rate $R(\rho \!\rightarrow \!\sigma )$ can be turned into a whole family of resource quantifiers: for a fixed resourceful state $\sigma$ (respectively, $\rho$), the function $R(\,\cdot \!\rightarrow \!\sigma )$ (respectively, $R(\rho \! \rightarrow \! \cdot \,)^{-1}$) is a resource quantifier with a solid operational interpretation. In entanglement theory, for example, considering free all those transformations that can be implemented with local operations assisted by classical communication (LOCC) and choosing as fixed states Bell pairs, the above procedure leads to the distillable entanglement and the entanglement cost, respectively [8, Section XV].

Since exact computations of asymptotic transformation rates are often challenging, it is important to establish rigorous bounds on them. In finite-dimensional resource theories, it is possible do so as follows: if *G* is a resource monotone, i.e., a function from quantum states to the set of nonnegative real numbers that does not increase under free operations, the inequality $R(\rho \!\rightarrow \!\sigma )\le \frac{G(\rho )}{G(\sigma )}$ holds if *G* is (i) additive on multiple copies of a state, and (ii) asymptotically continuous [9–13] (see also [3, Section VI.A.5]). Property (i) can be enforced by regularization [3, Section VI.A.4], and (ii) turns out to hold for many monotones in finite-dimensional systems. For infinite-dimensional resource theories, this approach is however not viable, because the conventional definition of asymptotic continuity, which involves the dimension *d* of the underlying Hilbert space, becomes meaningless. And indeed, in infinite dimensions many monotones — especially those based on entropic quantities — are discontinuous everywhere [14–16]. A weaker version of asymptotic continuity can be restored by imposing an energy constraint [16–19], yet doing so still does not result in any bound on the transformation rates, because the free operations employed are a priori *not* required to be (uniformly) energy-constrained. Due to this seemingly merely technical complication, we are not aware of any general technique to upper bound asymptotic transformation rates in infinite-dimensional quantum resource theories prior to our work. We dub this state of affairs the “asymptotic continuity catastrophe”.

This situation is particularly undesirable because infinite-dimensional systems, especially quantum harmonic oscillators, are ubiquitous in physics, and — as suggested by quantum field theory — perhaps fundamental. The optical modes that underlie the flourishing field of continuous variable (CV) quantum technologies [20–22] are a prime example, but harmonic oscillators appear whenever the behavior of a physical system close to equilibrium is approximated to second order.

Here, we devise a simple yet general way to circumvent the asymptotic continuity catastrophe, and establish rigorous bounds on transformation rates that are equally valid for finite- and infinite-dimensional quantum resource theories. Our approach relies on monotones *G* that satisfy, in addition to (i), also (ii’) lower semicontinuity, which is much weaker than (ii) and does not depend upon the Hilbert space dimension, and (iii) strong superaddivity, i.e., $G(\rho _{AB})\ge G(\rho _A) + G(\rho _B)$. We show how (i), (ii’), and (iii) combined imply the sought general bound $R(\rho \!\rightarrow \!\sigma )\le \frac{G(\rho )}{G(\sigma )}$ on the transformation rate (Theorem 15).

We then study three main applications, to the infinite-dimensional resource theories of: (I) optical nonclassicality [23–28]; (II) quantum entanglement [6–8]; and (III) continuous variable quantum thermodynamics [29, 30]. Each of these applications rests upon a different strongly superadditive monotone, namely (I) the measured relative entropy of nonclassicality, introduced and studied here, (II) the squashed entanglement [31–35], and (III) the free energy [29]. Albeit strong subadditivity was known to hold for the latter two monotones, it is only thanks to our Theorem 15 that we are able to employ this mathematical property to deduce an upper bound on asymptotic transformation rates. To the extent of our knowledge, ours are the first such bounds for any and thus in particular the above infinite-dimensional resource theories.

From the technical standpoint, our main results are obtained in the context of nonclassicality (I). Here, one of our key contributions is the proof of a handy variational expression for the measured relative entropy of nonclassicality. This result relies on a careful application of Sion’s Theorem, based, in turn, on a crucial and carefully made choice of topology on the space of trace class operators acting on an infinite-dimensional Hilbert space. This variational expression has several implications, most notably: (a) it immediately implies strong superadditivity, allowing us to deduce (b) the sought bound on asymptotic transformation rates; and finally (c) it points to a technique for estimating, up to arbitrary precision, said upper bound. Another useful result established here is the finiteness of our nonclassicality monotones for any state with either finite energy or finite Wehrl entropy. Other nonclassicality monotones, such as the standard robustness of nonclassicality [27, 28], often diverge, giving little to no information about the actual resource content of a state.

This manuscript is organized as follows. In Sect. 2 we introduce the basic notation and concepts of quantum mechanics for infinite-dimensional systems that will be used in the remainder of the work. Then, Sect. 3 features a concise review of the mathematical framework of quantum resource theories. In Sect. 4 we introduce our main results, in particular Theorems 15, 19, and 23, and briefly discuss their implications. The proofs start from Sect. 5, where we initiate the study of our monotones. In Sect. 6 we establish our first main result, Theorem 15, together with some of its consequences. Section 7 is devoted instead to the more involved proof of Theorems 19 and 23. In Sect. 8 we explore further properties of our nonclassicality monotones. In the subsequent Sect. 9 we apply them to study a wealth of examples, including noisy Fock states, Schrödinger cat states, and squeezed states; we also test our bounds on rates for the case of distillation and dilution of Fock states and cat states in the theory of nonclassicality. Appendix A is concerned with various restricted notions of asymptotic continuity for infinite-dimensional systems and with their limitations in proving general bounds on asymptotic transformation rates, which further motivates our analysis.

## Quantum Mechanics for Infinite-Dimensional Systems

With every quantum system one associates a Hilbert space $\mathcal {H}$; in this work we will be mainly concerned with the case where $\dim \mathcal {H}=\infty$, i.e., with infinite-dimensional quantum systems. When dealing with (both finite- and infinite-dimensional) open quantum systems, both physical states and observables are described in terms of linear self-adjoint operators acting on $\mathcal {H}$ and fulfilling specific properties. As we will discuss in a moment, the structure of operator spaces is more complex in the infinite-dimensional case than in the finite-dimensional one, where many of them coincide. We start by setting the basic notation and definitions and introduce the objects that we will use in the following.

### Notation and definitions

For a generic linear self-adjoint operator *X* acting on a Hilbert space $\mathcal {H}$ one can define the **operator norm** as follows:

$$\begin{aligned} \begin{aligned} { \Vert X\Vert _\infty :=\sup _{\vert \psi \rangle \in \mathcal {H}\setminus \{0\}}\frac{|\langle \psi |X|\psi \rangle |}{\langle \psi |\psi \rangle }\,. } \end{aligned} \end{aligned}$$

Operators with finite operator norm, i.e., $\Vert B\Vert _\infty <\infty$ are said to be **bounded**. Moreover, an operator is said to be **trace class** if its **trace norm**

$$\begin{aligned} \begin{aligned} \Vert T\Vert _1:=\sum _{n=0}^\infty \langle \phi _n|\sqrt{T^\dagger T}|\phi _n\rangle \end{aligned} \end{aligned}$$

is finite, where $\{\phi _n\}_n$ is any orthogonal basis of $\mathcal {H}$. For trace class operators we can define the **trace** as:

$$\begin{aligned} \begin{aligned} {{\,\textrm{Tr}\,}}[T]:=\sum _{n=0}^\infty \langle \phi _n|T|\phi _n\rangle \,, \end{aligned} \end{aligned}$$

which is independent of the choice of the orthogonal basis $\{\phi _n\}_n$. The trace norm of a trace class operator *T* can then be written as $\Vert T\Vert _1={{\,\textrm{Tr}\,}}\left[ \sqrt{T^\dagger T}\right]$.

We are now ready to list below the most relevant operator spaces:

- $\mathcal {B}_{\textrm{sa}}(\mathcal {H}_{})$: the Banach space of bounded self-adjoint operators on $\mathcal {H}$;
- $\mathcal {T}_{\textrm{sa}}(\mathcal {H}_{})$: the Banach space of self-adjoint trace class operators on $\mathcal {H}$;
- $\mathcal {K}_{\textrm{sa}}(\mathcal {H}_{})$: the Banach space of self-adjoint **compact** operators on $\mathcal {H}$, defined as the closure with respect to the operator norm of $\mathcal {T}_{\textrm{sa}}(\mathcal {H}_{})$;
- $\mathcal {D}(\mathcal {H}_{})$: the set of **density operators** (i.e., positive semidefinite trace class operators with trace 1) on $\mathcal {H}$;
- $\mathcal {T}_{\textrm{sa}}^+(\mathcal {H}_{})$: the cone of positive semidefinite (and hence self-adjoint) trace class operators on $\mathcal {H}$;
- $\mathcal {K}_{\textrm{sa}}^+(\mathcal {H}_{})$: the cone of positive semidefinite (and hence self-adjoint) compact operators on $\mathcal {H}$;

One has that $\mathcal {T}_{\textrm{sa}}(\mathcal {H}_{}) \subseteq \mathcal {K}_{\textrm{sa}}(\mathcal {H}_{}) \subseteq \mathcal {B}_{\textrm{sa}}(\mathcal {H}_{})$, with equality iff $\mathcal {H}$ is finite dimensional. Also, the duality relation $\mathcal {T}_{\textrm{sa}}(\mathcal {H}_{})^* = \mathcal {B}_{\textrm{sa}}(\mathcal {H}_{})$ holds at the level of Banach spaces. We remind the reader that the dual of a Banach space *X* equipped with a norm $\Vert \cdot \Vert _X$ is the vector space of all linear functionals $\varphi :X\rightarrow \mathbb {R}$ such that $\Vert \varphi \Vert _{X^*}:=\sup _{\Vert x\Vert _X\le 1} |\varphi (x)|<\infty$, equipped with the norm $\Vert \cdot \Vert _{X^*}$.

A **quantum state** on a quantum system *A* with Hilbert space $\mathcal {H}_A$ is represented by a density operator $\rho _A\in \mathcal {D}(\mathcal {H}_{A})$. **Quantum channels** from *A* to *B*, where *A*, *B* are quantum systems, are completely positive trace preserving maps $\Lambda :\mathcal {T}_{\textrm{sa}}(\mathcal {H}_{A})\rightarrow \mathcal {T}_{\textrm{sa}}(\mathcal {H}_{B})$. For a quantum channel $\Lambda :\mathcal {T}_{\textrm{sa}}(\mathcal {H}_{A})\rightarrow \mathcal {T}_{\textrm{sa}}(\mathcal {H}_{B})$, the **adjoint** $\Lambda ^\dag$ is the linear map $\Lambda ^\dag : \mathcal {B}_{\textrm{sa}}(\mathcal {H}_{B})\rightarrow \mathcal {B}_{\textrm{sa}}(\mathcal {H}_{A})$ defined by ${{\,\textrm{Tr}\,}}\left[ T_A \Lambda ^\dag (X_B)\right] :={{\,\textrm{Tr}\,}}\left[ \Lambda (T_A) X_B\right]$ for all $T_A\in \mathcal {T}_{\textrm{sa}}(\mathcal {H}_{A})$ and $X_B\in \mathcal {B}_{\textrm{sa}}(\mathcal {H}_{B})$. Among the simplest examples of quantum channels are **quantum measurements**, represented by **positive operator-valued measures** (POVM), i.e., finite collections $\mathcal {M}= \{E_x\}_{x\in \mathcal {X}}$ of positive semidefinite (bounded) operators $E_x \ge 0$ that obey the normalization rule $\sum _x E_x=\mathbb {1}$. Any quantum measurement can be written as a trace-preserving map by making use of classical flags $\{\vert \phi _x\rangle \}_{x\in \mathcal {X}}$: $\rho \mapsto \sum \nolimits _x{{\,\textrm{Tr}\,}}[\rho E_x]\rho _x\otimes \vert \phi _x\rangle \!\langle \phi _x\vert$, where $\rho _x$ is the output state in case the outcome *x* is measured.

It is well known that the topological structure of infinite-dimensional spaces is much richer than in the finite-dimensional case. There is a wealth of topologies that can be defined on infinite-dimensional Banach spaces, and in particular on the operator spaces discussed above [36]. In light of this fact, and for later convenience, we provide here a quick guide:

- the **weak operator topology** on $\mathcal {B}_{\textrm{sa}}(\mathcal {H}_{})$ (and hence on $\mathcal {T}_{\textrm{sa}}(\mathcal {H}_{})$ and $\mathcal {D}(\mathcal {H}_{})$) is the coarsest topology that makes all functionals $A \mapsto \langle \psi |A| \psi \rangle$ continuous, for all $\vert \psi \rangle \in \mathcal {H}$;
- the weak* topology on $\mathcal {T}_{\textrm{sa}}(\mathcal {H}_{})$ is the coarsest topology that makes all functionals $T\mapsto {{\,\textrm{Tr}\,}}[TK]$ continuous, for all $K\in \mathcal {K}_{\textrm{sa}}(\mathcal {H}_{})$;
- the **weak topology** on $\mathcal {T}_{\textrm{sa}}(\mathcal {H}_{})$ is the coarsest topology that makes all functionals $T\mapsto {{\,\textrm{Tr}\,}}[TA]$ continuous, for all $A\in \mathcal {B}_{\textrm{sa}}(\mathcal {H}_{})$;
- the **trace norm topology** on $\mathcal {T}_{\textrm{sa}}(\mathcal {H}_{})$ is the one induced by the trace norm $\Vert \cdot \Vert _1$;
- the **operator norm topology** on $\mathcal {B}_{\textrm{sa}}(\mathcal {H}_{})$ is the one induced by the operator norm $\Vert \cdot \Vert _\infty$.

The role of the weak* topology on $\mathcal {T}_{\textrm{sa}}(\mathcal {H}_{})$ will play a special role for us (cf. Lemma 38).

#### Remark 1

The weak* topology is the topology induced by the Banach space $\mathcal {K}_{\textrm{sa}}(\mathcal {H}_{})$ on its dual $\mathcal {K}_{\textrm{sa}}(\mathcal {H}_{})^*=\mathcal {T}_{\textrm{sa}}(\mathcal {H}_{})$. Therefore, by the Banach–Alaoglu theorem the unit ball $B_{\mathcal {T}_{\textrm{sa}}(\mathcal {H}_{})}:=\{T\in \mathcal {T}_{\textrm{sa}}(\mathcal {H}_{}): \Vert T\Vert _1\le 1\}$ of $\mathcal {T}_{\textrm{sa}}(\mathcal {H}_{})$ is weak* compact. This fact will be crucial for one of the main results of the work.

We conclude this section by stating some useful facts about operator topologies. We start by noting the following remarkable lemma, originally discovered by Davies [37, Lemma 4.3] — see also the ‘gentle measurement lemma’ by Winter [38, Lemma 9] for a refined version.

#### Lemma 2

[37, Lemma 4.3] For a net^1^$(\omega _\alpha )_\alpha \subseteq \mathcal {T}_{\textrm{sa}}^+(\mathcal {H}_{})$ of positive semidefinite trace class operators, if  in the weak operator topology, and moreover , then  in norm.

Since two topologies are equal if and only if they have the same convergent nets, it is immediate to deduce the following.

#### Corollary 3

The weak topology and the norm topology coincide on $\mathcal {T}_{\textrm{sa}}^+(\mathcal {H}_{})$. They also coincide with the weak operator topology on $\mathcal {D}(\mathcal {H}_{})$.

#### Remark 4

The norm topology does not coincide with the weak operator topology on $\mathcal {T}_{\textrm{sa}}^+(\mathcal {H}_{})$. For instance, the sequence of Fock states $\left( \vert n\rangle \!\langle n\vert \right) _n$ converges to 0 in the weak operator topology, but it is not convergent in the norm topology (for instance because it is not of Cauchy type).

### Continuous variable systems

Among all infinite-dimensional quantum systems, a central role is played by **continuous variable (CV) systems**, and here, perhaps most notably, by finite collections of harmonic oscillators. The Hilbert space corresponding to an *m*-mode CV system is composed of all square-integrable complex-valued functions on the Euclidean space $\mathbb {R}^m$, denoted with $\mathcal {H}_m=L^2(\mathbb {R}^m)$; one can then identify $\mathcal {H}_m\simeq \mathcal {H}_1^{\otimes m}$. Note that we will adopt the convention $\hbar =1$ hereafter. Then, the **canonical operators** $x_j$ and $p_j:=-i \frac{\partial }{\partial x_j}$ ($j=1,\ldots , m$) satisfy the **canonical commutation relations** $[x_j,x_k]= 0 = [p_j,p_k]$ and $[x_j,p_k]=i \delta _{jk} \mathbb {1}$, with $\mathbb {1}$ denoting the identity over $\mathcal {H}_m$. It is customary to define the annihilation and creation operators by

1 $$\begin{aligned} \begin{aligned} a_j:=\frac{x_j + i p_j}{\sqrt{2}}\, ,\qquad a_j^\dag :=\frac{x_j - i p_j}{\sqrt{2}}\, . \end{aligned} \end{aligned}$$

In terms of $a_j,a_j^\dag$, the canonical commutation relations take the form $[a_j,a_k]\equiv 0$, $[a_j, a_k^\dag ] = \delta _{jk} \mathbb {1}$.

On a single-mode system, **Fock states** are defined for $k\in \mathbb {N}$ by $\vert k\rangle :=\frac{1}{\sqrt{k!}} (a^\dag )^k\vert 0\rangle$, where $\vert 0\rangle$ is the **vacuum state**. For $\alpha \in \mathbb {C}$, the associated **coherent state** takes the form [39–42]

2 $$\begin{aligned} \vert \alpha \rangle :=e^{-\frac{|\alpha |^2}{2}}\sum _{k=0}^\infty \frac{\alpha ^k}{\sqrt{k!}}\, \vert k\rangle \, . \end{aligned}$$

Extending these definitions to multimode systems is quite straightforward. For $k = (k_1,\ldots , k_m)^\intercal \in \mathbb {N}^m$, one sets $\vert k\rangle :=\bigotimes _{j=1}^m \vert k_j\rangle$; analogously, for $\alpha = (\alpha _1,\ldots , \alpha _m)^\intercal \in \mathbb {C}^m$, a multimode coherent state is defined by $\vert \alpha \rangle :=\bigotimes _{j=1}^m \vert \alpha _j\rangle$.

The **displacement operators** form a special family of unitary operators acting on $\mathcal {H}_m$. For $\alpha \in \mathbb {C}^m$, they are defined by

3 $$\begin{aligned} {\mathscr {D}}(\alpha ):=\exp \left[ \sum \nolimits _{j=1}^m \left( \alpha _j a_j^\dag - \alpha _j^* a_j\right) \right] . \end{aligned}$$

They satisfy the identity

4 $$\begin{aligned} {\mathscr {D}}(\alpha ) {\mathscr {D}}(\beta ) = e^{\frac{1}{2}\left( \alpha ^\intercal \beta ^* - \alpha ^\dag \beta \right) }\,{\mathscr {D}}(\alpha +\beta )\, , \end{aligned}$$

called the **Weyl form of the canonical commutation relations**, for all $\alpha ,\beta \in \mathbb {C}^m$, and they yield coherent states upon acting on the vacuum, i.e.,

5 $$\begin{aligned} {\mathscr {D}}(\alpha ) \vert 0\rangle = \vert \alpha \rangle \qquad \forall \ \alpha \in \mathbb {C}^m\, . \end{aligned}$$

For an arbitrary trace class operator $T\in \mathcal {T}_{\textrm{sa}}(\mathcal {H}_{m})$, its **characteristic function** $\chi _T:\mathbb {C}^m\rightarrow \mathbb {C}$ is given by

6 $$\begin{aligned} \chi _T(\alpha ) :={{\,\textrm{Tr}\,}}[T{\mathscr {D}}(\alpha )]\, . \end{aligned}$$

For a *m*-mode quantum state $\rho \in \mathcal {D}(\mathcal {H}_{m})$, a quantity which is intimately related to its characteristic function is the **Husimi** ${\textbf{Q}}$**-function** $Q:\mathbb {C}^m\rightarrow \mathbb {C}$, defined by $Q_\rho (\alpha ) :=\frac{1}{\pi ^m} \langle \alpha |\rho |\alpha \rangle$ [43].

### Entropies and relative entropies

The (von Neumann) **entropy** of some positive semidefinite trace class operator $A\in \mathcal {T}_{\textrm{sa}}^+(\mathcal {H})$ can be defined as

7 $$\begin{aligned} S(A) :=- {{\,\textrm{Tr}\,}}\left[ A \log _2 A \right] . \end{aligned}$$

Note that this is a well-defined although possibly infinite quantity. One way to make sense of the expression (7) is via the infinite sum $S(A) = \sum _i (- a_i \log _2 a_i)$, where $A=\sum _i a_i \vert a_i\rangle \!\langle a_i\vert$ is the spectral decomposition of *A*  where we convene that $0\log _2 0=0$. Since  because *A* is trace class, the terms of the above sum are eventually positive. Hence, the sum itself can be assigned a well-defined value, possibly $+\infty$. An alternative approach is to define is the **Wehrl entropy** instead:

8 $$\begin{aligned} S_W(\rho ):=-\int d^{2m}\alpha \,Q_\rho (\alpha )\log _2 \left( \pi ^m\, Q_\rho (\alpha )\right) \,. \end{aligned}$$

It is well known that $S_W(\rho )\ge S(\rho )$ for any quantum state $\rho \in \mathcal {D}(\mathcal {H}_{})$.

The **relative entropy** between two positive $A,B\in \mathcal {T}_{\textrm{sa}}^+(\mathcal {H})$ is usually written as [44, 45]

9 $$\begin{aligned} D(A\Vert B) :={{\,\textrm{Tr}\,}}\left[ A (\log _2 A - \log _2 B)\right] . \end{aligned}$$

Again, the above expression is well defined and possibly infinite [46]. To see why, we represent it as the infinite sum $D(A \Vert B) :=\sum _{i,j} \left| \langle a_i | b_j\rangle \right| ^2 \left( a_i \log _2 a_i - a_i \log _2 b_j \right. \left. + \log _2(e) (b_j - a_i) \right) + \log _2(e) {{\,\textrm{Tr}\,}}[A-B]$, where $A = \sum _i a_i \vert a_i\rangle \!\langle a_i\vert$ and $B = \sum _j b_j \vert b_j\rangle \!\langle b_j\vert$ are the spectral decompositions of *A* and *B*, respectively, and we assume that only terms with $a_i>0$ and $b_j>0$ are included. As above, we follow the convention of setting $0\log _2 0=0$, and we set $D(A\Vert B)=+\infty$ if there exist two indices *i* and *j* with $a_i>0$, $b_j=0$, and $\langle a_i | b_j\rangle \ne 0$. As detailed in [46], the convexity of $a\mapsto a\log _2 a$ implies that all terms of the above infinite sum are non-negative, making the expression well defined. In light of the above discussion, it is not difficult to realize that a necessary condition for $D(A \Vert B)$ to be finite is that ${{\,\textrm{supp}\,}}A \subseteq {{\,\textrm{supp}\,}}B$. Thus, up to projecting everything onto a subspace we will often assume that *B* is faithful, i.e., that $B>0$. The relative entropy can be endowed with an operational interpretation in the context of asymmetric hypothesis testing [47–49].

An alternative approach to the quest for defining a quantum relative entropy could be that of bringing the problem back to the classical setting by means of quantum measurements. Namely, for a state $\rho$ and a measurement $\mathcal {M}= \{E_x\}_{x\in \mathcal {X}}$, we define the associated outcome probability distribution on $\mathcal {X}$ as $P^{\mathcal {M}}_\rho (x):={{\,\textrm{Tr}\,}}\left[ \rho E_x\right]$. Remembering that for two classical probability distributions *p* and *q* the **Kullback–Leibler divergence** is given by $D_{K\! L}\!(p\Vert q):=\sum _x p_x (\log _2 p_x - \log _2 q_x)$ [50], let us define the **measured relative entropy** between any two states $\rho$ and $\sigma$ as [47, 51]

10 $$\begin{aligned} D^M\!(\rho \Vert \sigma ) :=\sup _{\mathcal {M}} D_{K\! L}\!\left( P^{\mathcal {M}}_\rho \big \Vert P^{\mathcal {M}}_\rho \right) . \end{aligned}$$

It is known that $D^M\!(\rho \Vert \sigma )\le D(\rho \Vert \sigma )$ for all pairs of states $\rho ,\sigma$ [51]. Recently, extending a result by Petz [52], Berta et al. have shown that for finite-dimensional systems equality holds if and only if $[\rho ,\sigma ]=0$ [53].

## Quantum Resource Theories

In this section we introduce a general notion of quantum resource theory, and some related concepts and results. Note that our definition is slightly different from that in the recent review by Chitambar and Gour [3, Definition 1], in that we require also parallel composition (i.e., tensor product) of free operations to be free.

### Definition 5

A **quantum resource theory** (QRT) is a pair ${\mathscr {R}} = \left( {\mathscr {S}}, {\mathscr {F}} \right)$, where ${\mathscr {S}}$ is a family of quantum systems that is closed under tensor products, in the sense that $A,B\in {\mathscr {S}}$ implies that $AB :=A\otimes B \in {\mathscr {S}}$; and contains the trivial system 1 with Hilbert space $\mathbb {C}$, while ${\mathscr {F}}$, called the set of free operations, is a mapping that assigns to every pair of systems $A,B\in {\mathscr {S}}$ a set of channels from system *A* to *B*. Such a set will be denoted with ${\mathscr {F}} (A\rightarrow B)$.^2^ We will require that the following three consistency conditions are satisfied:

(i) For all $A\in {\mathscr {S}}$, the identity is a free operation on *A*, in formula $I_A \in {\mathscr {F}}(A\rightarrow A)$;
(ii) Free operations are closed under sequential compositions, namely, if $A,B,C\in {\mathscr {S}}$ and $\Lambda \in {\mathscr {F}}(A\rightarrow B)$, $\Gamma \in {\mathscr {F}}(B\rightarrow C)$, then also $\Gamma \circ \Lambda \in {\mathscr {F}}(A\rightarrow C)$;
(iii) Free operations are closed under parallel compositions, namely, if for $j=1,2$ one chooses $A_j,B_j\in {\mathscr {S}}$ and $\Lambda _j\in {\mathscr {F}}(A_j\rightarrow B_j)$, then also $\Lambda _1\otimes \Lambda _2 \in {\mathscr {F}}(A_1\otimes A_2\rightarrow B_1\otimes B_2)$.

If every system in ${\mathscr {S}}$ is finite dimensional, we will say that ${\mathscr {R}}$ itself is **finite dimensional**.

### Monotones

Given a QRT ${\mathscr {R}}$ as above, one defines the set of free states on the system $A\in {\mathscr {S}}$ as

11 $$\begin{aligned} {\mathscr {F}}_S(A) :={\mathscr {F}} (1\rightarrow A)\, . \end{aligned}$$

Clearly, if partial traces are free, then ${{\,\textrm{Tr}\,}}_A\left[ {\mathscr {F}}_S(AB)\right] \subseteq {\mathscr {F}}_S(B)$. A central role in our paper is played by resource quantifiers, i.e., monotones. We define them as follows.

#### Definition 6

Let ${\mathscr {R}} = \left( {\mathscr {S}}, {\mathscr {F}} \right)$ be a resource theory. A mapping *G* assigning to each $A\in {\mathscr {S}}$ a function $G_A:\mathcal {D}(\mathcal {H}_{A})\rightarrow [0,+\infty ]$ on the set of states on *A* that takes on values in the extended reals $[0,+\infty ]$ is called a **resource monotone** — or simply a **monotone** — if

(i) $G_B \left( \Lambda (\rho )\right) \le G_A (\rho )$ holds for all states $\rho$ on $A\in {\mathscr {S}}$ and for all free operations $\Lambda \in {\mathscr {F}}(A\rightarrow B)$, where $B\in {\mathscr {S}}$ is arbitrary;
(ii) $G_A(\sigma )=0$ for all $\sigma \in {\mathscr {F}}_S(A)$, with ${\mathscr {F}}_S(A)$ defined by (11).

A monotone *G* is said to be:

- **Faithful**, if $G_A(\rho )=0$ implies that $\rho \in {\mathscr {F}}_S(A)$;
- **Convex**, if all functions $G_A$ are convex, i.e., $G_A\left( \sum \nolimits _i p_i \rho _i \right) \le \sum _i p_i G_A(\rho _i)$ for all $A\in {\mathscr {S}}$ and all statistical ensembles $\{p_i, \rho _i\}$ on *A*;
- **Asymptotically continuous** [9, 11–13] on some subsets of systems ${\mathscr {S}}'\subseteq {\mathscr {S}}$, if for all $A\in {\mathscr {S}}'$ we have that $\dim \mathcal {H}_A<\infty$, and moreover there exist two continuous functions $f,g:[0,1]\rightarrow \mathbb {R}$ independent of $\dim \mathcal {H}_A$ such that $f(0) = g(0) = 0$ and
  12 $$\begin{aligned} \left| G_A(\rho ) - G_A(\sigma ) \right| \le f(\epsilon ) \log (\dim \mathcal {H}_A) + g(\epsilon ) \end{aligned}$$
  for all $\rho ,\sigma \in \mathcal {D}(\mathcal {H}_{A})$ at trace distance $\epsilon :=\frac{1}{2} \left\| \rho -\sigma \right\| _1$.
- **Lower semicontinuous**, if $G_A$ is lower semicontinuous as a function on $\mathcal {D}(\mathcal {H}_{A})$ for all $A\in {\mathscr {S}}$, i.e., if $\lim _{n\rightarrow \infty }\left\| \rho _n-\rho \right\| _1=0$ for a sequence of states on *A* implies that $\liminf _{n\rightarrow \infty } G_A(\rho _n)\ge G_A(\rho )$;
- **Strongly superadditive**, if $G_{AB}(\rho _{AB}) \ge G_A(\rho _A) + G_B(\rho _B)$ holds for all $A,B\in {\mathscr {S}}$ and for all states $\rho _{AB}\in \mathcal {D}(\mathcal {H}_{AB})$;
- **Superadditive**, if $G_{AB}(\rho _A \otimes \sigma _B) \ge G_A(\rho _A) + G_B(\sigma _B)$ for all $A,B\in {\mathscr {S}}$ and for all states $\rho _{A}\in \mathcal {D}(\mathcal {H}_{A})$ and $\sigma _B\in \mathcal {D}(\mathcal {H}_{B})$;
- **Weakly superadditive**, if $G_{A_1\ldots A_n}\left( \rho ^{\otimes n}\right) \ge n\, G_A(\rho )$ for all $A\in {\mathscr {S}}$, for all *n* and all states $\rho \in \mathcal {D}(\mathcal {H}_{A})$, where $A_1\ldots A_n$ denotes the joint system formed by *n* copies of *A*;
- **Additive**, if $G_{AB}(\rho _A \otimes \sigma _B) = G_A(\rho _A) + G_B(\sigma _B)$ for all $A,B\in {\mathscr {S}}$ and for all states $\rho _{A}\in \mathcal {D}(\mathcal {H}_{A})$ and $\sigma _B\in \mathcal {D}(\mathcal {H}_{B})$;
- **Weakly additive**, if $G_{A_1\ldots A_n}\left( \rho ^{\otimes n}\right) = n G_A(\rho )$ for all $A\in {\mathscr {S}}$ and all states $\rho \in \mathcal {D}(\mathcal {H}_{A})$, where $A_1\ldots A_n$ denotes the joint system formed by *n* copies of *A*.

#### Remark 7

In Definition 6, $\text {(e)}\Rightarrow \text {(f)}\Rightarrow \text {(g)}$ and $\text {(h)}\Rightarrow \text {(j)}$.

#### Remark 8

Any monotone is automatically invariant under free unitaries whose inverse is also free.

#### Remark 9

The notions of upper semicontinuous, strongly subadditive, or subadditive monotone are obtained by reversing the inequalities and exchanging $\liminf$ with $\limsup$ in (d), (e), and (f) of Definition 6.

#### Note

In what follows, with a slight abuse of notation we will often drop the subscript of *G* specifying the system it refers to, and think of a monotone *G* as a function defined directly on the collection of states on all possible systems $A\in {\mathscr {S}}$.

### Transformation rates

We continue by recalling the definition of asymptotic transformation rate.

#### Definition 10

Let $({\mathscr {S}},{\mathscr {F}})$ be a QRT. For any two systems $A,B\in {\mathscr {S}}$ and any two states $\rho _A\in \mathcal {D}(\mathcal {H}_{A})$ and $\sigma _B\in \mathcal {D}(\mathcal {H}_{B})$, the corresponding **(standard) asymptotic transformation rate** is given by

13 $$\begin{aligned} R(\rho _{A} \rightarrow \sigma _{B}) :=\sup \left\{ r: \lim _{n\rightarrow \infty } \inf _{\Lambda _n\in {\mathscr {F}}\left( A^n\rightarrow B^{\left\lfloor rn\right\rfloor }\right) } \left\| \Lambda _n\left( \rho _{A}^{\otimes n}\right) - \sigma _{B}^{\otimes \left\lfloor r n\right\rfloor } \right\| _1 = 0 \right\} , \end{aligned}$$

where $A^n$ denotes the system composed of *n* copies of *A*. Any number $r>0$ in the set on the right-hand side of (13) is called a (standard) **achievable rate** for the transformation $\rho _{A} \rightarrow \sigma _{B}$.

The above definition captures the intuitive notion of maximum yield of copies of the target state $\sigma _B$ that can be obtained per copy of the initial state $\rho _A$ by means of free operations and with asymptotically vanishing error. In Definition 10, we have measured the error using the global trace distance. However, it is possible and sometimes even reasonable to modify the error criterion. For instance, in a situation where the output copies are distributed to noninteracting parties, what is relevant is the maximum local error rather than the global one. This train of thought inspires the following definition.

#### Definition 11

Let $({\mathscr {S}},{\mathscr {F}})$ be a QRT. For any two systems $A,B\in {\mathscr {S}}$ and any two states $\rho _A\in \mathcal {D}(\mathcal {H}_{A})$ and $\sigma _B\in \mathcal {D}(\mathcal {H}_{B})$, the corresponding **maximal asymptotic transformation rate** is given by

14 $$\begin{aligned}{} & {} {\widetilde{R}}(\rho _{A} \rightarrow \sigma _{B})\nonumber \\{} & {} \quad :=\sup \left\{ r : \lim _{n\rightarrow \infty } \inf _{\Lambda _n\in {\mathscr {F}}\left( A^n\rightarrow B^{\left\lfloor rn\right\rfloor }\right) } \max _{j=1,\ldots , \left\lfloor rn\right\rfloor } \left\| \left( \Lambda _n \left( \rho _{A}^{\otimes n}\right) \right) _{j} - \sigma _{B} \right\| _1\! =\! 0 \right\} , \nonumber \\ \end{aligned}$$

where for a state $\Omega \in \mathcal {D}(\mathcal {H}_{B^{k}})$ defined on *k* copies of *B* we defined $\Omega _j:={{\,\textrm{Tr}\,}}_{B^k\setminus B_j} \left[ \Omega \right] \in \mathcal {D}(\mathcal {H}_{B_j})$ as the reduced state on the $j^{\text {th}}$ subsystem. Any number $r>0$ in the set on the right-hand side of (13) is called a **maximally achievable rate** for the transformation $\rho _{A} \rightarrow \sigma _{B}$.

It is immediate to see that for any given pair of states the maximal rate always upper bounds the corresponding standard rate (Lemma 31).

### Infinite-dimensional quantum resource theories

The prime example of a quantum resource theory is naturally that of entanglement [3, 6, 8, 54]. In spite of their central importance, very little is known about many fundamental operational questions in the infinite-dimensional case [16, 35, 55, 56], with a partial exception being the theory of Gaussian entanglement [57–61]. We can formally define entanglement as a resource theory as follows.

#### Definition 12

The resource theory of bipartite **entanglement** is defined by setting:

- ${\mathscr {S}}$ to be the family of all (possibly infinite-dimensional) quantum systems $A\!:\!B$, where the colon indicates the bipartition in separate parties;
- ${\mathscr {F}}_S(A\!:\!B) = \text {Sep }(A\!:\!B) :={\overline{{{\,\textrm{conv}\,}}}}\left\{ \vert \phi _A\rangle \!\langle \phi _A\vert \otimes \vert \psi _B\rangle \!\langle \psi _B\vert :\,\vert \phi _A\rangle \in \mathcal {H}_A,\,\vert \psi _B\rangle \in \mathcal {H}_B \right\}$ for any bipartite system $A\!:\!B\in {\mathscr {S}}$, where ${\overline{{{\,\textrm{conv}\,}}}}$ denotes the closed (in trace norm topology) convex hull of a set;
- ${\mathscr {F}}(A\!:\!B\rightarrow A'\!:\!B')=\text {LOCC }(A\!:\!B\rightarrow A'\!:\!B')$ is the set of all *LOCC* protocols from $A\!:\!B$ to $A'\!:\!B'$.

Another important example is the resource theory of quantum thermodynamics [29, 62, 63]. Just as that of entanglement, it can be constructed for finite-dimensional systems as well. However, in accordance with the spirit of this work, we will focus on the continuous variable case from now on.

#### Definition 13

The resource theory of **quantum thermodynamics** is defined by setting:

- ${\mathscr {S}}$ to be the family of all (possibly infinite-dimensional) quantum systems *A* equipped with a Hamiltonian $H_A$ satisfying the Gibbs hypothesis, i.e., ${{\,\textrm{Tr}\,}}\left[ \exp ^{-\beta H_A}\right] <\infty$ for any inverse temperature $\beta >0$; we also assume $H_{AB}=H_A+H_B$ for any systems $A,B\in {\mathscr {S}}$;
- Once an inverse temperature $\beta$ has been fixed for all systems, ${\mathscr {F}}_S(A) = \{\gamma _A\}$, with
  $$\begin{aligned} \gamma _A:=\frac{\exp ^{-\beta H_A}}{{{\,\textrm{Tr}\,}}\left[ \exp ^{-\beta H_A}\right] } \end{aligned}$$
  being the **thermal state**, for any system $A\in {\mathscr {S}}$ with Hamiltonian $H_A$;
- ${\mathscr {F}}(A\rightarrow B)$ to encompass all quantum channels $\Lambda :\mathcal {T}_{\textrm{sa}}(\mathcal {H}_{A})\rightarrow \mathcal {T}_{\textrm{sa}}(\mathcal {H}_{B})$ such that $\Lambda (\gamma _A)=\gamma _B$ for systems $A,B\in {\mathscr {S}}$ with thermal states $\gamma _A$ and $\gamma _B$ respectively (**Gibbs-preserving operations**).

In the case where the family ${\mathscr {S}}$ contains only continuous variable quantum systems, other specific resource theories emerge naturally, as a result of operational or technological constraints. For example, the resource theory of optical nonclassicality [23–28] is based on the premise that statistical mixtures of coherent states are easy to synthesize, hence free, and “classical”, as they most closely approximate classical electromagnetic waves. On the other hand, operationally, nonclassical states, such as Fock states [64, 65], squeezed states [66–70], cat states [71–78], or NOON states [79, 80], play an increasingly central role in applications. A formal definition of this resource theory is as follows.

#### Definition 14

The resource theory of (**optical**) **nonclassicality** is defined by setting:

- ${\mathscr {S}}$ to be the family of all continuous variable quantum systems;
- ${\mathscr {F}}_S(A) = \mathcal {C}_m :={\overline{{{\,\textrm{conv}\,}}}}\left\{ \vert \alpha \rangle \!\langle \alpha \vert :\, \alpha \in \mathbb {C}^m\right\}$ for any *m*-mode system $A\in {\mathscr {S}}$;
- ${\mathscr {F}}(A\rightarrow B)$, with $A,B\in {\mathscr {S}}$ being *m* and $m'$-mode systems respectively, to encompass all quantum channels $\Lambda :\mathcal {T}_{\textrm{sa}}(\mathcal {H}_{m})\rightarrow \mathcal {T}_{\textrm{sa}}(\mathcal {H}_{m'})$ such that $\Lambda (\mathcal {C}_m)\subseteq \mathcal {C}_{m'}$ (**classical operations**).

The so-called classical operations comprise, but are possibly not limited to, channels that can be obtained through passive linear optics, destructive measurements, and feed-forward of measurement outcomes [24, 25]. Note that the set of classical states and that of classical channels are both convex.

## Main Results

In the present section we state the main results of our work. The starting point is the general bound on asymptotic transformation rates in Theorem 15. We then explore its consequences for the resource theories of entanglement and quantum thermodynamics in Corollaries 16 and 17, respectively. To apply it to the resource theory of nonclassicality, instead, we need to introduce and study two new monotones, the measured relative entropy of nonclassicality $N^M_r\!$ and its regularized version (Definition 18). One of our main technical contributions is the proof of a powerful variational expression for $N^M_r\!$ (Theorem 19), from which we deduce the lower semicontinuity and — most importantly — the strong superadditivity of the measured relative entropy of nonclassicality $N^M_r\!$ (Theorem 23).

### General bound on asymptotic rates

Our first result is a general bound on (maximal) transformation rates that works for all quantum resource theories, including infinite-dimensional ones. Formally, it is a generalization of a well-known bound holding for many monotones in finite dimensions [3, Section VI.A.5]. In this simpler context, it is possible to prove that if a resource monotone *G* is weakly additive and moreover asymptotically continuous (Definition 6, (j) and (c)) then the asymptotic transformation rate $R(\rho \!\rightarrow \!\sigma )$ between any two states $\rho ,\sigma$ (Definition 10) satisfies that

15 $$\begin{aligned} R(\rho \!\rightarrow \!\sigma )\le \frac{G(\rho )}{G(\sigma )}\, , \end{aligned}$$

provided that the right-hand side is well defined. This bound has been proved in [12, Theorem 4] (see also [3, Section VI.A.5]) using techniques developed in [10] and [11] — see especially [11, Propositions 19, 20, and 22] for a thorough discussion of many possible variations of the underlying hypotheses. In fact, the proof is so simple and enlightening that it is worth summarising it here. For any achievable rate *r*, i.e., for any element of the set in (13), calling *A*, *B* the systems to which $\rho ,\sigma$ pertain, we can construct the sequence of maps $\Lambda _n\in {\mathscr {F}}\left( A^n\rightarrow B^{\left\lfloor rn\right\rfloor }\right)$ such that $\epsilon _n :=\frac{1}{2} \left\| \Lambda _n\left( \rho ^{\otimes n}\right) - \sigma ^{\otimes \left\lfloor r n\right\rfloor } \right\| _1$ satisfies $\lim _{n\rightarrow \infty } \epsilon _n = 0$. Leveraging properties (j) and (c) in Definition 6, one then obtains that

16 $$\begin{aligned} \begin{aligned} n\, G(\rho )\,&{\mathop {=}\limits ^{{\text {(j) }}}}\, G\left( \rho ^{\otimes n}\right) \\&\!\ge {} G\left( \Lambda _n\left( \rho ^{\otimes n}\right) \right) \\&{\mathop {\ge }\limits ^{{\text {(c) }}}} G\big (\sigma ^{\otimes \left\lfloor rn\right\rfloor }\big ) - f(\epsilon _n) \log \big ( d^{\left\lfloor rn\right\rfloor } \big ) - g(\epsilon _n) \\&{\mathop {=}\limits ^{{\text {(j) }}}} \left\lfloor rn\right\rfloor \left( G(\sigma ) - f(\epsilon _n) \log d \right) - g(\epsilon _n)\, , \end{aligned}\end{aligned}$$

where we called $d:=\dim \mathcal {H}_B$. Dividing by *n* and taking the limit $n\rightarrow \infty$ yields $r\le G(\rho )/G(\sigma )$, and in turn (15) once one takes the supremum over *r*.

Inequality (15), applied to different weakly additive and asymptotically continuous monotones, yields many of the commonly employed bounds on rates as far as finite-dimensional resources are concerned.^3^ Unfortunately, for infinite-dimensional systems the notion of asymptotic continuity becomes empty, and consequently the argument in (16) breaks down at the step marked as (c). In fact, prior to our work there seemed to be a lack of technical tools to address the approximation error allowed in the transformation (13) in the infinite-dimensional case. Due to this “asymptotic continuity catastrophe”, in the existing literature prior to our work we could not locate *any* upper bound on asymptotic transformation rates that holds for infinite-dimensional resource theories. The theorem below, whose proof is remarkably simple (and yet very different from that in (16) but whose applicability is surprisingly wide, remedies this regrettable state of affairs at least in the case where the employed monotone *G* is strongly superadditive. An alternative but ultimately less satisfactory approach to circumvent the asymptotic continuity catastrophe is sketched out in Appendix A, to which we refer the reader interested in more details on this point.

#### Theorem 15

For a given QRT, not necessarily finite dimensional, let *G* be a monotone that is strongly superadditive, weakly additive, and lower semicontinuous. Then, for all states $\rho _A,\sigma _B$, it holds that

17 $$\begin{aligned} R(\rho _A\!\rightarrow \!\sigma _B)\le {\widetilde{R}}(\rho _A\!\rightarrow \!\sigma _B) \le \frac{G(\rho _A)}{G(\sigma _B)} \,, \end{aligned}$$

whenever the rightmost side is well defined.

With the above result at hand, we can now establish rigorous bounds on transformation rates in operationally important examples of infinite-dimensional quantum resource theories.

### First consequences: resource theories of entanglement and quantum thermodynamics

We start with the QRT of entanglement. To the extent of our knowledge, there is no available technique to derive upper bounds on the transformation rate $R(\rho _{AB}\rightarrow \sigma _{AB})$ in terms of known monotones. Even the energy-constrained version of asymptotic continuity established by Shirokov [17–19] for many entanglement monotones does not suffice to this purpose. This is because we need continuity estimates on the output system, and — while the input, consisting of many copies of a known state, is naturally energy constrained — the output, being produced by a general unconstrained free channel, is not. We could of course impose such an energy constraint artificially, by enforcing the parties to operate with LOCCs that are *uniformly* energy-constrained; however, the operational motivation behind this assumption is somewhat dubious; this is especially so if energy is much cheaper than entanglement, which is often the case in experimental practice. We refer the reader to Appendix A for a more in-depth discussion of these points.

To apply Theorem 15 to the case at hand, we need an entanglement monotone that obeys strong superadditivity. The *squashed entanglement*, denoted $E_{sq}$, is a natural candidate [31–34]. Shirokov [35] has shown how to extend its definition to infinite-dimensional systems [35, Eq. (17)]. We report the definition of squashed entanglement later in Sect. 6.2. Applying Theorem 15 to it we deduce the following corollary.

#### Corollary 16

Let $\rho _{AB}$ and $\sigma _{A'B'}$ be two bipartite states such that

18 $$\begin{aligned} \min \left\{ S(\rho _A),\, S(\rho _B),\, S(\rho _{AB})\right\}<\infty \, ,\quad \min \left\{ S(\sigma _{A'}),\, S(\sigma _{B'}),\, S(\sigma _{A'B'})\right\} <\infty \, . \end{aligned}$$

Then, in the QRT of entanglement it holds that

19 $$\begin{aligned} R(\rho _{AB}\!\rightarrow \!\sigma _{A'B'})\le {\widetilde{R}}(\rho _{AB}\!\rightarrow \!\sigma _{A'B'}) \le \frac{E_{sq}(\rho _{AB})}{E_{sq}(\sigma _{A'B'})}\, . \end{aligned}$$

Another possible application of Theorem 15 is to the QRT of thermodynamics. The quantity $G(\rho _A):=\frac{1}{\beta } D(\rho _A\Vert \gamma _A)$, which coincides with the free energy difference between $\rho _A$ and $\gamma _A$ when ${{\,\textrm{Tr}\,}}\left[ \rho _A H_A\right] <\infty$, can be seen to be strongly superadditive, additive and lower semicontinuous. We deduce the following.

#### Corollary 17

In the QRT of thermodynamics, for all states $\rho _{A}, \sigma _{B}$ it holds that

20 $$\begin{aligned} R(\rho _{A}\!\rightarrow \!\sigma _{B})\le {\widetilde{R}}(\rho _{A}\!\rightarrow \!\sigma _{B}) \le \frac{D(\rho _A\Vert \gamma _A)}{D(\sigma _B\Vert \gamma _B)} \,. \end{aligned}$$

Let us stress that Corollary 17 extends the results of Brandão et al. [29], which are valid in finite-dimensional systems, to *all* quantum systems where a QRT of thermodynamics can be constructed. This is clearly a crucial improvement because of the ubiquity of harmonic oscillators in physical applications.

### Further consequences: resource theory of nonclassicality

Over the past decades, there have been proposals to quantify the nonclassicality of quantum states of light, e.g., by their distance from the set of classical states [84–87], by the amount of noise needed in order to make them classical [88, 89], by their potential for entanglement generation [90–92] or for metrological advantage [93], by the negativity [94, 95], the variances [25] or other features [96–101] of their phase-space distributions, or by the minimum number of superposed coherent states needed to reproduce the target state [102]. Unfortunately, none of these monotones appears to yield bounds on asymptotic transformation rates, for they fail to satisfy asymptotic continuity. In fact, to the extent of our knowledge, no rigorous bounds on those rates are known for the resource theory of optical nonclassicality. Indeed, the transformations considered in Yadin et al. [25, Theorems 2 and 3] are probabilistic but exact, and moreover single-shot rather than asymptotic. One could argue that especially their zero-error nature somewhat limits their operational relevance in applications.

We therefore pursue a different approach. In analogy to what was previously done for entanglement [103, 104], we use the relative entropies introduced in Sect. 2.3 to construct nonclassicality measures.

#### Definition 18

Let $\rho \in \mathcal {D}(\mathcal {H}_{m})$ be an *m*-mode state. The **relative entropy of nonclassicality** and the **measured relative entropy of nonclassicality** of $\rho$ are defined respectively as:

21 $$\begin{aligned} N_r(\rho ) :=\inf _{\sigma \in \mathcal {C}_m}D(\rho \Vert \sigma )\, ,\quad N^M_r\!(\rho ) :=\inf _{\sigma \in \mathcal {C}_m}D^M\!(\rho \Vert \sigma )\, . \end{aligned}$$

Note that our definition of $N_r$ differs from that of Marian et al. [86], in that $\sigma$ is allowed to be an arbitrary classical state, not necessarily Gaussian. It is not difficult to see that $N_r$ and $N^M_r\!$ are faithful and convex nonclassicality monotones (Lemma 28). Since $N_r$ is also subadditive (again, Lemma 28), its regularization $N_r^\infty (\rho ) :=\lim _{n\rightarrow \infty }\! \frac{N_r(\rho ^{\otimes n})}{n}$ is well defined by Fekete’s lemma [105] and also subadditive (Corollary 29). We will show that both $N_r$ and $N_r^\infty$ are always finite on bounded-energy states (Proposition 30), but that there exist infinite-energy states $\rho$ such that $N_r(\rho )=N_r^\infty (\rho )=\infty$ (Proposition 39). Explicit computations or tight estimates for the measured relative entropy of nonclassicality for Fock-diagonal states, squeezed states, and cat states are reported in Sects. 9.1–9.2.

It might not be clear at this point why to introduce $N^M_r\!$ alongside with $N_r$, given that the former quantity involves one more nested optimization than the latter. However, we now show that its computation can be notably simplified.

#### Theorem 19

For all *m*-mode finite-entropy states $\rho$, it holds that

22 $$\begin{aligned} N^M_r\!(\rho ) = \sup _{L>0} \left\{ {{\,\textrm{Tr}\,}}\left[ \rho \log _2 L\right] - \log _2 \sup _{\alpha \in \mathbb {C}^m} \langle \alpha | L | \alpha \rangle \right\} \,, \end{aligned}$$

where *L* ranges over all positive trace class operators on $\mathcal {H}_m$ (equivalently, on all positive normalized states).

The proof of Theorem 19 involves two main ingredients. This first one is a generalization of the variational program for $D^M\!$ put forth by Berta et al. [53, Lemma 1] to the infinite-dimensional case, which may be of independent interest.

#### Lemma 20

Let $\rho \in \mathcal {D}(\mathcal {H}_{})$ be a density operators on a (possibly infinite-dimensional) Hilbert space $\mathcal {H}$, and let $\sigma \in \mathcal {T}_{\textrm{sa}}^+(\mathcal {H}_{})$ be positive semidefinite and nonzero. Then

23 $$\begin{aligned} D^M\!(\rho \Vert \sigma )&= \sup _{h\in \mathcal {B}_{\textrm{sa}}(\mathcal {H}_{})} \left\{ {{\,\textrm{Tr}\,}}\left[ \rho h\right] - \log _2 {{\,\textrm{Tr}\,}}\left[ \sigma 2^h\right] \right\} \end{aligned}$$

24 $$\begin{aligned}&= \sup _{h\in \mathcal {B}_{\textrm{sa}}(\mathcal {H}_{})} \left\{ {{\,\textrm{Tr}\,}}\left[ \rho h\right] + \log _2 (e) \left( 1 - {{\,\textrm{Tr}\,}}\left[ \sigma 2^h\right] \right) \right\} \end{aligned}$$

25 $$\begin{aligned}&= \sup _{0<\delta \mathbb {1}<L\in \mathcal {B}_{\textrm{sa}}(\mathcal {H}_{})} \left\{ {{\,\textrm{Tr}\,}}\left[ \rho \log _2 L\right] - \log _2 {{\,\textrm{Tr}\,}}\left[ \sigma L\right] \right\} \end{aligned}$$

26 $$\begin{aligned}&= \sup _{0<\delta \mathbb {1}<L\in \mathcal {B}_{\textrm{sa}}(\mathcal {H}_{})} \left\{ {{\,\textrm{Tr}\,}}\left[ \rho \log _2 L\right] + \log _2 (e) \left( 1 - {{\,\textrm{Tr}\,}}\left[ \sigma L\right] \right) \right\} \end{aligned}$$

27 $$\begin{aligned}&= \sup _{0<L\in \mathcal {B}_{\textrm{sa}}(\mathcal {H}_{})} \left\{ {{\,\textrm{Tr}\,}}\left[ \rho \log _2 L\right] - \log _2 {{\,\textrm{Tr}\,}}\left[ \sigma L\right] \right\} \end{aligned}$$

28 $$\begin{aligned}&= \sup _{0<L\in \mathcal {B}_{\textrm{sa}}(\mathcal {H}_{})} \left\{ {{\,\textrm{Tr}\,}}\left[ \rho \log _2 L\right] + \log _2 (e) \left( 1 - {{\,\textrm{Tr}\,}}\left[ \sigma L\right] \right) \right\} . \end{aligned}$$

The notation $L>\delta \mathbb {1}$ in the supremum in equations (25) and (26) means that *L* is required to have eigenvalues bounded from below by a positive quantity, i.e., to satisfy $L>\delta \mathbb {1}$ for some $\delta >0$, depending on *L*.

#### Remark 21

For the case where the measurements in (10) are restricted to be projective (i.e., $\mathcal {M}=\{E_x\}_{x\in \mathcal {X}}$ with $E_x$ a projector for all *x*, and $\sum \nolimits _x E_x=\mathbb {1}$), the expression in (23) has been obtained already by Petz [45, Proposition 7.13]).

The above Lemma 20 is proved in Sect. 7.1. By applying it to the program in (21) we are left with a nested optimization of the form $\sup \inf$. Then, the second critical ingredient that is needed to arrive at a proof of Theorem 19 is an application of Sion’s minimax theorem [106] that allows us to exchange infimum and supremum in this resulting expression. This is technically challenging, as meeting the compactness hypothesis in Sion’s theorem requires a careful choice of topology on the domain of optimization. The crucial technical contribution here is Lemma 38, which establishes the compactness of the set of subnormalized classical states with respect to the weak*-topology (see Sect. 2.1). Along the way, we introduce and study an auxiliary quantity $\Gamma$ (Definition 33 and Proposition 35).

An immediate consequence of Theorem 19 is the superadditivity of $N^M_r\!$ on finite-entropy states. This fact allows us to successfully construct the regularization $N^{M,\infty }_r\!$.

#### Corollary 22

When computed on finite-entropy states, $N^M_r\!$ is lower semicontinuous and strongly superadditive, meaning that

29 $$\begin{aligned} N^M_r\!(\rho _{AB}) \ge N^M_r\!(\rho _A) + N^M_r\!(\rho _B)\qquad \forall \ \rho _{AB}:\ S(\rho _A), S(\rho _B) < \infty \, . \end{aligned}$$

Therefore, for any finite-entropy state $\rho$ its regularization

30 $$\begin{aligned} N^{M,\infty }_r\!(\rho )\!:=\!\lim _{n\rightarrow \infty } \!\frac{N^M_r\!(\rho ^{\otimes n})}{n} \end{aligned}$$

is a well defined nonclassicality monotone. It is lower semicontinuous, strongly superadditive, and weakly additive. Furthermore,

31 $$\begin{aligned} N^M_r\!(\rho ) \le N^{M,\infty }_r\!(\rho ) \le N_r^\infty (\rho ) \le N_r(\rho ) \end{aligned}$$

holds whenever $S(\rho )<\infty$. In particular, both $N_r^\infty$ and $N^{M,\infty }_r\!$ are also faithful, at least on finite-entropy states.

The variational expression in Theorem 19 has many more consequences. For example, we use it to establish upper and lower bounds on $N^M_r\!$ and its regularization $N^{M,\infty }_r\!$ based on the Wehrl entropy (Proposition 40), which translate to tight estimates of these quantifiers for Gaussian states (Corollary 41). The most important application is however the following.

#### Theorem 23

Let $\rho ,\sigma$ be two CV states with finite entropy, i.e., such that $S(\rho ),S(\sigma )<\infty$. Then the transformation rates in the resource theory of nonclassicality obey the inequalities

32 $$\begin{aligned} R(\rho \!\rightarrow \!\sigma )\le {\widetilde{R}}(\rho \!\rightarrow \!\sigma ) \le \frac{N^{M,\infty }_r\!(\rho )}{N^{M,\infty }_r\!(\sigma )} \le \frac{N_r(\rho )}{N^M_r\!(\sigma )} \,, \end{aligned}$$

provided that the ratios on the right-hand sides are well defined.

To the best of our knowledge, (32) is the first explicit bound on asymptotic transformation rates in the context of CV nonclassicality. However, it would amount to a rather futile theoretical statement if not complemented with a systematic way of upper bounding the ratio $N_r(\rho )/N^M_r\!(\sigma )$. Note that $N_r$ can be estimated from above by simply making suitable ansatzes in (21). The a priori less trivial task of lower bounding $N^M_r\!$ can be carried out thanks to Theorem 19.

**Fig. 1:**
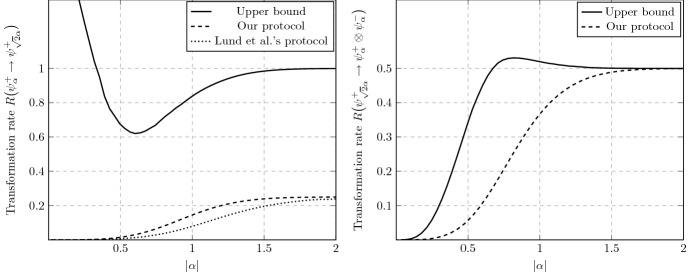
Upper and lower bounds on asymptotic transformation rates of Schrödinger cat states

As an immediate application of Theorem 23, we consider the paradigmatic example of (Schrödinger) cat state manipulation [78, 107–111]. For $\alpha \in \mathbb {C}$, cat states are defined by [71]

33 $$\begin{aligned} \vert \psi _{\alpha }^\pm \rangle :=\frac{1}{\sqrt{2\left( 1 \pm e^{-2|\alpha |^2}\right) }} \left( \vert \alpha \rangle \pm \vert -\alpha \rangle \right) , \end{aligned}$$

where $\vert \pm \alpha \rangle$ are coherent states (2). The transformations we look at are $\psi _\alpha ^+\rightarrow \psi _{\sqrt{2} \alpha }^+$ (amplification) and $\psi _{\sqrt{2}\alpha }^+\rightarrow \psi _{\alpha }^+\otimes \psi _{\alpha }^-$ (sign-randomized dilution). A protocol for amplification using linear optical elements and quadrature measurements has been designed by Lund et al. [107]. We present an ameliorated version of it (Proposition 48), together with a simple protocol for sign-randomized dilution (Proposition 49). The lower bounds on rates given by these explicit protocols are shown in Fig. 1. The upper bound derived via Theorem 23 is asymptotically tight for the dilution task, but not in the case of amplification. This is due to the fact that our quantifiers all saturate to 1 for cat states with $|\alpha |\rightarrow \infty$.

## Preliminary Results

Throughout this section we lay the ground for the proof of our main results, studying general properties of monotone regularization (Sect. 5.1) and investigating in more detail nonclassicality monotones (Sect. 5.2).

### Generalities about monotone regularization

It turns out that any monotone *G* can be made weakly additive by a procedure known as “regularization”.

#### Definition 24

Let $\left( {\mathscr {S}}, {\mathscr {F}} \right)$ be a QRT equipped with a monotone *G*. Then the functions

34 $$\begin{aligned} G^{\downarrow ,\infty }(\rho )&:=\liminf _{n\rightarrow \infty } \frac{1}{n}\, G\left( \rho ^{\otimes n}\right) , \end{aligned}$$

35 $$\begin{aligned} G^{\uparrow ,\infty }(\rho )&:=\limsup _{n\rightarrow \infty } \frac{1}{n}\, G\left( \rho ^{\otimes n}\right) \end{aligned}$$

are called the **lower and upper regularizations** of *G*. On the domain of states $\rho$ such that $G^{\downarrow ,\infty }(\rho ) = G^{\uparrow ,\infty }(\rho )=:G^{\infty }(\rho )$ one can speak of a unique **regularization** $G^\infty$.

The following result is immediate from the definition.

#### Lemma 25

Let $\left( {\mathscr {S}}, {\mathscr {F}} \right)$ be a QRT equipped with a monotone *G*. Then the lower and upper regularizations $G^{\downarrow ,\infty }$ and $G^{\uparrow ,\infty }$ given by Definition 24 are also monotones. Moreover, $G^\infty$ is weakly additive on its domain, i.e., $G^{\downarrow ,\infty }(\rho ) = G^{\uparrow ,\infty }(\rho )$ for a state $\rho$ implies that $G^{\infty }(\rho ^{\otimes n})\equiv n\, G(\rho )$ for all $n\in \mathbb {N}_+$.

#### Proof

Let us start by showing that, e.g., $G^{\downarrow ,\infty }$ is a monotone. Since parallel composition of free operations is free, for all $\rho \in \mathcal {D}(\mathcal {H}_{A})$ and for all $\Lambda \in {\mathscr {F}}\left( A\rightarrow B\right)$, with $A,B\in {\mathscr {S}}$, we obtain that

$$\begin{aligned} G^{\downarrow ,\infty }\left( \Lambda (\rho ) \right)= & {} \liminf _{n\rightarrow \infty } \frac{1}{n}\, G\left( \Lambda (\rho )^{\otimes n}\right) = \liminf _{n\rightarrow \infty } \frac{1}{n}\, G\left( \Lambda ^{\otimes n} \left( \rho ^{\otimes n}\right) \right) \\\le & {} \liminf _{n\rightarrow \infty } \frac{1}{n}\, G\left( \rho ^{\otimes n} \right) = G^{\downarrow ,\infty } (\rho )\, . \end{aligned}$$

Moreover, if $\rho$ is free, also $\rho ^{\otimes n}$ is so, and hence $G^{\downarrow , \infty }(\rho )=0$ as well. This proves the first claim.

Now, by definition $G^{\downarrow ,\infty }(\rho ) = G^{\uparrow ,\infty }(\rho )$ implies that the sequence $\left( {\frac{1}{k}} G(\rho ^{\otimes k}) \right) _{k\in \mathbb {N}_+}$ has a limit. If that is the case, then clearly $G^{\infty }(\rho ^{\otimes n}) = \lim _{k\rightarrow \infty } \frac{1}{k}\, G\left( \rho ^{\otimes kn}\right) = n \lim _{n\rightarrow \infty } \frac{1}{kn}\, G\left( \rho ^{\otimes kn}\right) = n \, G^\infty (\rho )$ for all $n\in \mathbb {N}_+$. $\square$

A useful fact that is slightly less obvious is as follows.

#### Lemma 26

Let $\left( {\mathscr {S}}, {\mathscr {F}} \right)$ be a QRT equipped with a monotone *G* that is weakly superadditive. Then:

(i) The regularization $G^\infty$ in Definition 24 exists for all states $\rho$, i.e., $G^{\downarrow ,\infty }(\rho ) = G^{\uparrow ,\infty }(\rho )=:G^\infty (\rho )$ for all $\rho \in \mathcal {D}(\mathcal {H}_{A})$ with $A\in {\mathscr {S}}$; it is also weakly additive and satisfies $G^\infty {\ge }G$;
(ii) If *G* is also (strongly) superadditive, then $G^{\infty }$ is (strongly) superadditive as well;
(iii) If *G* is lower semicontinuous, then so is $G^\infty$.

#### Remark 27

The above result is still valid if we replace superadditivity with subadditivity, lower semicontinuity with upper semicontinuity, and reverse all inequalities.

#### Proof of Lemma 26

Due to weak superadditivity, for all states $\rho$ the sequence $\left( a_n\right) _{n\in \mathbb {N}_+}$ defined by $a_n :=G(\rho ^{\otimes n})$ is superadditive, meaning that $a_{n+m}\ge a_n+a_m$. Therefore, by Fekete’s lemma [105] $\lim _{n\rightarrow \infty }\frac{a_n}{n}$ exists, and it satisfies that $\lim _{n\rightarrow \infty }\frac{a_n}{n} = \sup _{n\in \mathbb {N}_+} \frac{a_n}{n}$. Therefore,

$$\begin{aligned} G^\infty (\rho ) = \lim _{n\rightarrow \infty } \frac{1}{n}\, G\left( \rho ^{\otimes n} \right) = \sup _{n\in \mathbb {N}_+} \frac{1}{n}\, G\left( \rho ^{\otimes n} \right) \end{aligned}$$

is well defined for all $\rho$, and satisfies $G^\infty (\rho )\ge G(\rho )$. This proves (i).

Now we proceed to prove points (ii) and (iii). We already saw in Lemma 25 that $G^\infty$ is a weakly additive monotone, so it suffices to show that it is (strongly) superadditive if *G* was such. This is immediate to establish (we prove it only for strong superadditivity, as the superadditivity case is completely analogous):

$$\begin{aligned} G^\infty (\rho _{AB})= & {} \lim _{n\rightarrow \infty } \frac{1}{n}\, G\left( \rho _{AB}^{\otimes n} \right) \ge \lim _{n\rightarrow \infty } \frac{1}{n}\, \left( G\left( \rho _{A}^{\otimes n} \right) + G\left( \rho _{B}^{\otimes n} \right) \right) = \lim _{n\rightarrow \infty } \frac{1}{n}\, G\left( \rho _{A}^{\otimes n} \right) \\{} & {} + \lim _{n\rightarrow \infty } \frac{1}{n}\, G\left( \rho _{B}^{\otimes n} \right) . \end{aligned}$$

To see that $G^\infty$ is lower semicontinuous if so was *G*, just notice that $G^\infty (\rho ) = \sup _{n\in \mathbb {N}_+} \frac{1}{n}\, G\left( \rho ^{\otimes n} \right)$ is the pointwise supremum of lower semicontinuous functions and thus must itself be lower semicontinuous. $\square$

### Nonclassicality monotones

If the reader is worried by the proliferation of regularized measures in Definition 18, they should not be. In fact, we will show that the regularizations are unique in all physically interesting cases. We are able to readily prove the equality between $N_r^{\downarrow ,\infty }$ and $N_r^{\uparrow ,\infty }$, while a proof for $N_r^{M,\downarrow ,\infty }$ and $N_r^{M,\uparrow ,\infty }$ will be given at the end of Sect. 7.4. The first step is to prove that the quantity we just defined are actually good resource monotones.

#### Lemma 28

The quantities $N_r$ and $N^M_r\!$ are faithful and convex nonclassicality monotones. They obey the inequality $N_r\ge N^M_r\!$. Moreover, $N_r$ is subadditive.

#### Proof

The argument is completely standard. The inequality $N_r\ge N^M_r\!$ is obvious, and follows from the same relation between the relative entropy and its measured version. Since both $D(\cdot \Vert \cdot )$ and $D^M\!(\cdot \Vert \cdot )$ obey the data processing inequality, for every classical channel $\Lambda :\mathcal {T}_{\textrm{sa}}(\mathcal {H}_{m})\rightarrow \mathcal {T}_{\textrm{sa}}(\mathcal {H}_{m'})$ we obtain that

$$\begin{aligned} N_r\left( \Lambda (\rho )\right)= & {} \inf _{\sigma '\in \mathcal {C}_{m'}} D\left( \Lambda (\rho ) \big \Vert \sigma ' \right) \le \inf _{\sigma '\in \Lambda \left( \mathcal {C}_{m'}\right) } D\left( \Lambda (\rho ) \big \Vert \sigma ' \right) \\= & {} \inf _{\sigma \in \mathcal {C}_m} D\left( \Lambda (\rho ) \big \Vert \Lambda (\sigma ) \right) \le \inf _{\sigma \in \mathcal {C}_m} D\left( \rho \Vert \sigma \right) = N_r(\rho )\, , \end{aligned}$$

and analogously for $N^M_r\!$. This proves monotonicity.

Convexity descends from the fact that both $N_r$ and $N^M_r\!$ are defined as the infimum of a jointly convex function on a convex domain. For example,

$$\begin{aligned}&N_r\left( \sum \nolimits _i p_i \rho _i\right) = \inf _{\sigma \in \mathcal {C}_m} D\left( \sum \nolimits _i p_i \rho _i\, \big \Vert \, \sigma \right) \\&= \inf _{\{\sigma _i\}_i\subseteq \mathcal {C}_m} D\left( \sum \nolimits _i p_i \rho _i\, \big \Vert \, \sum \nolimits _i p_i \sigma _i \right) \\&\le \inf _{\{\sigma _i\}_i\in \mathcal {C}_m} \sum _i p_i\, D\left( \rho _i \Vert \sigma _i \right) \\&= \sum _i p_i\, \inf _{\sigma _i \in \mathcal {C}_m}D\left( \rho _i \Vert \sigma _i \right) \\&= \sum _i p_i\, N_r(\rho _i)\, . \end{aligned}$$

The proof for $N^M_r\!$ is entirely analogous.

Faithfulness follows, e.g., from Pinsker’s inequality $D_{K\! L}\!(p\Vert q)\ge \frac{1}{2} \log _2(e) \Vert p-q\Vert _1^2$ [112], which implies that

$$\begin{aligned} D^M\!(\rho \Vert \sigma )= & {} \sup _{\mathcal {M}} D_{K\! L}\!\left( P_\rho ^\mathcal {M}\big \Vert P_\sigma ^\mathcal {M}\right) \ge \frac{1}{2} \log _2(e) \sup _{\mathcal {M}} \left\| P_\rho ^\mathcal {M}- P_\sigma ^\mathcal {M}\right\| _1^2 \\{} & {} = \frac{1}{2} \log _2(e)\, \Vert \rho -\sigma \Vert _1^2\, , \end{aligned}$$

where in the last line we used the elementary fact that the trace distance is achieved by the (binary) measurement $\{\Pi ,\mathbb {1}-\Pi \}$, with $\Pi$ being the projector onto the positive subspace of $\rho -\sigma$.

To prove the subadditivity of $N_r$, just notice that for all $(m+n)$-mode CV systems *AB* it holds that

$$\begin{aligned} N_r(\rho _A\otimes \sigma _B)&= \inf _{\sigma _{AB} \in \mathcal {C}_{m+n}} D\left( \rho _A\otimes \sigma _B \Vert \sigma _{AB}\right) \\&\le \inf _{\sigma _A\otimes \sigma _B\in \mathcal {C}_{m+n}} D\left( \rho _A\otimes \sigma _B \Vert \sigma _A \otimes \sigma _B \right) \\&= \inf _{\sigma _A\in \mathcal {C}_m,\, \sigma _B\in \mathcal {C}_n} \left\{ D\left( \rho _{A} \Vert \sigma _A \right) + D\left( \rho _{B} \Vert \sigma _B \right) \right\} \\&= N_r(\rho _A) + N_r(\rho _B)\, , \end{aligned}$$

where in the third line we used the identity [45, Eq. (5.22)]. $\square$

#### Corollary 29

The functions $N_r^{M,\downarrow ,\infty }, N_r^{M,\uparrow ,\infty }$ are nonclassicality monotones. The regularization $N_r^{\downarrow ,\infty } = N_r^{\uparrow , \infty }=:N_r^\infty$ is unique and is a weakly additive nonclassicality monotone; it satisfies that $N_r^\infty \le N_r$.

#### Proof

Follows directly from Lemmata 28 and 26. $\square$

We now argue that the monotones $N_r, N^M_r\!$ behave like useful resource quantifiers on states of physical interest. An essential basic feature is finiteness on bounded-energy states, where the energy is measured by the total photon number Hamiltonian.

#### Proposition 30

Let $\rho$ be an *m*-mode state with finite mean photon number $E:={{\,\textrm{Tr}\,}}\left[ \rho \left( \sum \nolimits _{j=1}^m a_j^\dag a_j \right) \right] < \infty$. Then

36 $$\begin{aligned} N^M_r\!(\rho ) \le N_r(\rho ) \le m\, g(E/m)\, , \end{aligned}$$

where $$g(x):=(x+1)\log _2 (x+1) - x\log _2 x$$.

#### Proof

It is well known that the entropy of an *m*-mode state with finite mean photon number *E* is at most *mg*(*E*/*m*), which indeed corresponds to the entropy of the thermal state with the same energy. Hence, $\rho$ has finite entropy, so that (31) holds. Thus, we only have to show that $N_r(\rho )\le m g(E/m)$. For an arbitrary $\nu \ge 0$, let

37 $$\begin{aligned} \tau _\nu :=\frac{1}{1+\nu } \sum _{n=0}^\infty \left( \frac{\nu }{1+\nu }\right) ^n \vert n\rangle \!\langle n\vert = \frac{1}{1+\nu } \left( \frac{\nu }{1+\nu }\right) ^{a^\dag a} \end{aligned}$$

be the single-mode thermal state of mean photon number $\nu$. It is well known that $\tau _\nu \in \mathcal {C}_1$, and hence $\tau _\nu ^{\otimes m} \in \mathcal {C}_m$, for all $\nu \in [0,\infty )$. Therefore,

$$\begin{aligned} N_r(\rho )&\le \inf _{\nu \ge 0} D\left( \rho \, \big \Vert \, \tau _\nu ^{\otimes m} \right) \\&= \inf _{\nu \ge 0} \left\{ - S(\rho ) + m \log _2 (1+\nu ) - E \log _2 \left( \frac{\nu }{1+\nu } \right) \right\} \\&= - S(\rho ) + m\, g\left( E/m \right) \, , \end{aligned}$$

where we used the variational representation

$$\begin{aligned} g(x) = \inf _{\nu \ge 0} \left\{ \log _2(1+\nu ) - x \log _2 \left( \frac{\nu }{1+\nu }\right) \right\} , \end{aligned}$$

whose proof is elementary. $\square$

Further results on our nonclassicality monotones will be given in Sect. 8.

## Proof of Theorem 15 and of Corollaries 16 and 17

### Proof of Theorem 15

In this section we prove our first main result, Theorem 15. We start with a simple lemma, which justifies the name of maximal asymptotic transformation rate given to the quantity in Definition 11 (cf. Definition 11).

#### Lemma 31

Let $({\mathscr {S}},{\mathscr {F}})$ be a QRT. For any two systems $A,B\in {\mathscr {S}}$ and any two states $\rho _A\in \mathcal {D}(\mathcal {H}_{A})$ and $\sigma _B\in \mathcal {D}(\mathcal {H}_{B})$, it holds that

38 $$\begin{aligned} R(\rho _{A} \rightarrow \sigma _{B}) \le {\widetilde{R}}(\rho _{A} \rightarrow \sigma _{B})\, . \end{aligned}$$

#### Proof

For all *n* and all free operations $\Lambda _n \in {\mathscr {F}}\left( A^n\rightarrow B^{\left\lfloor rn\right\rfloor }\right)$, the data processing inequality for the trace norm [113] implies that

$$\begin{aligned} \max _{j=1,\ldots , \left\lfloor rn\right\rfloor } \left\| \left( \Lambda _n \left( \rho _{A}^{\otimes n}\right) \right) _{j} - \sigma _{B} \right\| _1 \le \left\| \Lambda _n\left( \rho _{A}^{\otimes n}\right) - \sigma _{B}^{\otimes \left\lfloor r n\right\rfloor } \right\| _1\, . \end{aligned}$$

Therefore, a sequence of protocols that achieves a rate *r* in (13) (i.e., that makes the global error vanish) achieves the same rate in (14) (because the maximum local error will also vanish). The claim follows. $\square$

We are now ready to present the proof of Theorem 15.

#### Proof of Theorem 15

It suffices to show that ${\widetilde{R}}(\rho _A\!\rightarrow \!\sigma _B) \le \frac{G(\rho _A)}{G(\sigma _B)}$. For any sequence of free operations $\Lambda _n\in {\mathscr {F}}\left( A^n \rightarrow B^{\left\lfloor rn\right\rfloor }\right)$ satisfying , for all *j*, $\liminf _{n\rightarrow \infty }\left\| \left( \Lambda _n \left( \rho _{A}^{\otimes n}\right) \right) _{j} - \sigma _{B} \right\| _1=0$ it holds that

$$\begin{aligned} G(\rho _A)&{\mathop {=}\limits ^{{\text{1 }}}} \liminf _{n\rightarrow \infty } \frac{1}{n}\, G\big ( \rho _A^{\otimes n} \big ) \\&{\mathop {\ge }\limits ^{{\text{2 }}}} \liminf _{n\rightarrow \infty } \frac{1}{n}\, G\left( \Lambda _n\big (\rho _A^{\otimes n}\big )\right) \\&{\mathop {\ge }\limits ^{{\text{3 }}}} \liminf _{n\rightarrow \infty } \frac{1}{n}\, \sum _{j=1}^{\left\lfloor rn\right\rfloor } G\left( \left( \Lambda _n\big (\rho _A^{\otimes n}\big )\right) _j\right) \\&\ge \liminf _{n\rightarrow \infty } \frac{\left\lfloor rn\right\rfloor }{n} \min _j G\left( \left( \Lambda _n\big (\rho _A^{\otimes n}\big )\right) _j\right) \\&{\mathop {=}\limits ^{{\text{4 }}}} \liminf _{n\rightarrow \infty } \frac{\left\lfloor rn\right\rfloor }{n} G\left( \left( \Lambda _n\big (\rho _A^{\otimes n}\big )\right) _{j_n}\right) \\&= r \liminf _{n\rightarrow \infty } G\left( \left( \Lambda _n\big (\rho _A^{\otimes n}\big )\right) _{j_n}\right) \\&{\mathop {\ge }\limits ^{{\text{5 }}}} r\, G(\sigma _B)\, . \end{aligned}$$

Here, 1 holds due to weak additivity, even without the lim inf and for every *n*; 2 comes from monotonicity; 3 from strong superadditivity; in 4 we constructed a sequence of indices $j_n$ achieving the minimum; finally, 5 descends from lower semicontinuity and the assumption on $\Lambda _n$. Then a supremum over *r* yields the claim. $\square$

Before moving on to the study of the applications, it is perhaps instructive to compare the above argument with the one we saw in (16), where the same bound on rates was proved (under different assumptions) in the finite-dimensional setting. The main difference lies in step 3, in which we exploit strong superadditivity to move the error analysis to the single-copy level, where it is ultimately tackled by means of lower semicontinuity (step 5). In (16), instead, asymptotic continuity was leveraged to carry out an error analysis directly at the many-copy level. This type of ideas had been previously exploited in [114, Theorem 4 and Remark 10].

### Proof of Corollary 16

We now apply Theorem 15 to the resource theory of entanglement. Let us start by fixing some terminology. The **squashed entanglement** of a bipartite state $\rho _{AB}$ of a finite-dimensional bipartite system *AB* is defined by [31–34]

39 $$\begin{aligned} E_{sq}(\rho _{AB}) :=\frac{1}{2} \inf _{\rho _{ABE}} I(A:B|E)_\rho \, , \end{aligned}$$

where the infimum is over all extensions $\rho _{ABE}$ of the state $\rho _{AB}$, i.e., over all tripartite states $\rho _{ABE}$ satisfying that ${{\,\textrm{Tr}\,}}_E \left[ \rho _{ABE}\right] =\rho _{AB}$, and

40 $$\begin{aligned} I(A:B|E)_\rho :=S(\rho _{AE}) + S(\rho _{BE}) - S(\rho _E) - S(\rho _{ABE}) \end{aligned}$$

is the conditional mutual information. The problem with the above definition is that it cannot be extended directly to the infinite-dimensional case, because the right-hand side of (40) may contain the undefined expression $\infty -\infty$ [35, 56].

Fortunately, Shirokov has found a way out of this impasse. The first step is to construct the conditional mutual information via an alternative expression to (40), namely,

41 $$\begin{aligned} I(A:B|E)_\rho = \sup _{\Pi _A} \left\{ I(A:BE)_{\Pi _A \rho \Pi _A} - I(A:E)_{\Pi _A \rho \Pi _A} \right\} , \end{aligned}$$

where the supremum is over all finite-dimensional projectors $\Pi _A$ on *A*. An equivalent expression is obtained by exchanging *A* and *B* in (41). Clearly, (41) reduces to (40) when *A* is finite dimensional.

With (41) at hand, (39) can be extended without difficulty to the infinite-dimensional case [35, Eq. (17)]. In order for this to work, we have to keep in mind that the system *E* could and in general will be infinite-dimensional.

An alternative strategy to generalize the squashed entanglement to infinite-dimensional systems could be that of truncating the state directly by means of local finite-dimensional projectors. This results in a different function $\hat{E}_{sq}$, defined by [35, Eq. (37)]

42 $$\begin{aligned} \hat{E}_{sq}(\rho _{AB}) :=\sup _{\Pi _A, \Pi _B} E_{sq}\left( (\Pi _A\otimes \Pi _B)\, \rho _{AB}\, (\Pi _A\otimes \Pi _B) \right) , \end{aligned}$$

where the infimum runs over all finite-dimensional projectors $\Pi _A$ and $\Pi _B$. The nested optimizations hidden in (42) make $\hat{E}_{sq}$ a slightly less desirable quantity than $E_{sq}$. Nevertheless, we will find it useful in intermediate computations.

The main properties of the two functions $E_{sq}$ and $\hat{E}_{sq}$ that we will use are as follows:

- Both $E_{sq}$ and $\hat{E}_{sq}$ are strongly superadditive [35, Propositions 2B and 3B];
- Both $E_{sq}$ and $\hat{E}_{sq}$ are additive, and hence also weakly additive [35, Propositions 2B and 3B];
- $\hat{E}_{sq}$ is lower semicontinuous everywhere [35, Proposition 3A];
- $E_{sq}(\rho _{AB}) \equiv \hat{E}_{sq}(\rho _{AB})$ on all states with $\min \left\{ S(\rho _A),\, S(\rho _B),\, S(\rho _{AB}) \right\} <\infty$ [35, Proposition 3C].

#### Proof of Corollary 16

It suffices to write that

$$\begin{aligned} R(\rho _{AB}\!\rightarrow \!\sigma _{A'B'})\le {\widetilde{R}} (\rho _{AB}\!\rightarrow \!\sigma _{A'B'}) {\mathop {\le }\limits ^{{\text{1 }}}} \frac{\hat{E}_{sq}(\rho _{AB})}{\hat{E}_{sq}(\sigma _{A'B'})} {\mathop {=}\limits ^{{\text{2 }}}} \frac{E_{sq}(\rho _{AB})}{E_{sq}(\sigma _{A'B'})}\, , \end{aligned}$$

where 1 is just an application of Theorem 15, made possible by properties (a), (b), and (c) above, while 2 follows from (18) and property (d). $\square$

### Proof of Corollary 17

We now move on to the case of quantum thermodynamics at some (fixed) inverse temperature $\beta >0$. The monotone [29]

43 $$\begin{aligned} G(\rho _A):=\frac{1}{\beta }D(\rho _A \Vert \gamma _A) \end{aligned}$$

is easily seen to be:

- Strongly superadditive, because
  $$\begin{aligned} G(\rho _{AB}) = \frac{1}{\beta }D(\rho _{AB} \Vert \gamma _{AB}) = \frac{1}{\beta }D(\rho _{AB} \Vert \gamma _A \otimes \gamma _B) \ge \frac{1}{\beta }D(\rho _A \Vert \gamma _A) + \frac{1}{\beta }D(\rho _B \Vert \gamma _B)\, , \end{aligned}$$
  where the first identity is a consequence of the fact that $H_{AB}=H_A+H_B$, while the inequality follows from [45, Corollary 5.21];
- Additive and hence weakly additive, since
  $$\begin{aligned} G(\rho _A \otimes \sigma _B)= & {} \frac{1}{\beta }D\left( \rho _A \otimes \sigma _B \Vert \gamma _{AB} \right) = \frac{1}{\beta }D\left( \rho _A \otimes \sigma _B \Vert \gamma _A \otimes \gamma _B \right) = \frac{1}{\beta }D\left( \rho _A \Vert \gamma _A \right) \\{} & {} + \frac{1}{\beta }D\left( \sigma _B \Vert \gamma _B \right) ; \end{aligned}$$
  finally,
- Lower semicontinuous, as follows, e.g., from [45, Proposition 5.23].

#### Proof of Corollary 17

Thanks to properties (a), (b), and (c) above, the claim follows directly from Theorem 15. $\square$

## The Long March Towards Theorems 19 and 23

Throughout this section, we introduce all the necessary technical tools to arrive at a proof of Theorems 19 and 23. Along the way, we prove also Lemma 20 (Sect. 7.1) and Corollary 22 (Sect. 7.4)

### Proof of the variational expression for the measured relative entropy (Lemma 20)

The main goal of this subsection is to prove Lemma 20, which extends to the infinite-dimensional case the variational expressions for the measured relative entropy introduced in [53].

Let us start by highlighting the main differences and similarities between the six variational expressions reported in Lemma 20, reported here for the reader’s convenience: 
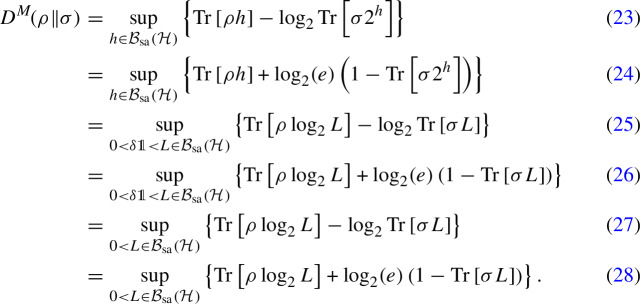

- We see immediately that they can be grouped in pairs: (23) and (24); (25) and (26); finally, (27) and (28). The two expressions in each pair involve an optimization over exactly the same set, and differ only by the objective function, which contains a $-\log _2 x$ in (23), (25), and (27), and its linearized version $\log _2(e) (1-x)$ in (24), (26), and (28).
- The programs in (25) and (26) contain an optimization over all bounded operators *L* that are also bounded away from 0, i.e., such that $L\ge \delta \mathbb {1}$ for some $\delta >0$, where $\mathbb {1}$ is the identity on $\mathcal {H}$.
- In the programs (27) and (28) we instead removed this latter constraint, and optimized only on positive operators $L>0$. Of course, this is a priori not the same: in infinite dimensions, it can happen — e.g., for any strictly positive density operator — that $L>0$ but there is no uniform bound $L\ge \delta \mathbb {1}>0$.
- Since in (27) and (28) the operator $\log _2 L$ is possibly unbounded from below, it may happen that ${{\,\textrm{Tr}\,}}[\rho \log _2 L]=-\infty$. This is not a problem, because we always have that ${{\,\textrm{Tr}\,}}[\sigma L]>0$ and hence $-\log _2 {{\,\textrm{Tr}\,}}[\sigma L]<+\infty$; therefore, the first addend is the only one that may diverge, and no uncertainties of the form $-\infty +\infty$ can arise in the objective function.

#### Proof of Lemma 20

Following the above observations, we divide the proof in several smaller steps.

1. Let us start by showing that (23) is equivalent to (24), (25) to (26), and (27) to (28). We only present the argument for the equivalence between (23) and (24), as the others are entirely analogous. First, from the inequality $\log _2 x\le \log _2(e) (x-1)$ we see that
  $$\begin{aligned} {{\,\textrm{Tr}\,}}[\rho h]-\log _2{{\,\textrm{Tr}\,}}\left[ \sigma 2^h\right] \ge {{\,\textrm{Tr}\,}}[\rho h] + \log _2(e) \left( 1-{{\,\textrm{Tr}\,}}\left[ \sigma 2^h\right] \right) \end{aligned}$$
  for any *h*. At the same time, the expression (23) is manifestly invariant under transformations of the type $h\mapsto h+\lambda I$ for any $\lambda \in \mathbb {R}$. So, we can always choose a $\lambda$ in both expressions such that ${{\,\textrm{Tr}\,}}\left[ \sigma 2^h\right] =1$, thus saturating the aforementioned inequality.
2. Now, observe that (23) is equivalent to (25), upon a change in parametrization $h=\log _2 L$. In fact, $\log _2 L$ is bounded if and only if *L* itself is bounded and moreover $L\ge \delta \mathbb {1}>0$. This implies that the variational expressions in (23), (24), (25), and (26) all coincide.
3. We now show that they also coincide with those in (27) and (28). Clearly, since the optimization in (27) is over a larger set than that in (25), its value cannot decrease. Therefore, to prove equality we only have to prove that
  $$\begin{aligned}{} & {} \sup _{0<\delta \mathbb {1}<L\in \mathcal {B}_{\textrm{sa}}(\mathcal {H}_{})} \left\{ {{\,\textrm{Tr}\,}}\left[ \rho \log _2 L\right] - \log _2 {{\,\textrm{Tr}\,}}\left[ \sigma L \right] \right\} \\{} & {} \quad \ge \sup _{0<L\in \mathcal {B}_{\textrm{sa}}(\mathcal {H}_{})} \left\{ {{\,\textrm{Tr}\,}}\left[ \rho \log _2 L\right] - \log _2 {{\,\textrm{Tr}\,}}[\sigma L] \right\} . \end{aligned}$$
  To this end, pick a bounded $L>0$, and let us show how to construct a family of bounded $L_\delta \ge \delta \mathbb {1}>0$ such that
  44 $$\begin{aligned} \lim _{\delta \rightarrow 0^+}\left\{ {{\,\textrm{Tr}\,}}\left[ \rho \log _2 L_\delta \right] - \log _2{{\,\textrm{Tr}\,}}[\sigma L_\delta ] \right\} = {{\,\textrm{Tr}\,}}[\rho \log _2 L] - \log _2{{\,\textrm{Tr}\,}}[\sigma L]\, . \end{aligned}$$
  Since the expression ${{\,\textrm{Tr}\,}}[\rho \log _2 L]-\log _2{{\,\textrm{Tr}\,}}[\sigma L]$ is clearly scale-invariant in *L*, i.e., it takes the same value for *L* and $\lambda L$, for all $\lambda >0$, we can assume without loss of generality that $L\le \mathbb {1}/2$. For $0<\delta \le 1/2$, set $L_\delta :=L + \delta \mathbb {1}\ge \delta \mathbb {1}$.
  Using the spectral theorem for bounded operators [115, Theorem 7.12], we can find a projection-valued measure $\mu$ on [0, 1/2] such that $L=\int _0^{1/2} \lambda d\mu (\lambda )$ and therefore $L_\delta = \int _0^{1/2} (\lambda +\delta ) d\mu (\lambda )$. Defining the real-valued measure $\mu _\rho$ on [0, 1/2] such that $\mu _\rho (X) = {{\,\textrm{Tr}\,}}[\rho \mu (X)]$ for all measurable sets $X\subseteq [0,1/2]$, we have that
  $$\begin{aligned} {{\,\textrm{Tr}\,}}\left[ \rho \left( -\log _2 L\right) \right]= & {} \int _0^{1/2} (-\log _2 \lambda ) d\mu _\rho (\lambda )\, ,\qquad \\ {{\,\textrm{Tr}\,}}\left[ \rho \left( -\log _2 L_\delta \right) \right]= & {} \int _0^{1/2} \left( -\log _2 (\lambda +\delta )\right) d\mu _\rho (\lambda )\, . \end{aligned}$$
  Since the functions $\lambda \mapsto -\log _2 (\lambda +\delta )$ are pointwise monotonically decreasing in $\delta$, converge pointwise to $\lambda \mapsto -\log _2 \lambda$, and all the functions involved are nonnegative, we can apply Beppo Levi’s monotone convergence theorem [116] (see also [117, Theorem 11.28]) and conclude that
  $$\begin{aligned} \lim _{\delta \rightarrow 0^+} {{\,\textrm{Tr}\,}}\left[ \rho \left( -\log _2 L_\delta \right) \right]= & {} \lim _{\delta \rightarrow 0^+} \int _0^{1/2} \left( -\log _2 (\lambda +\delta )\right) d\mu _\rho (\lambda ) \\= & {} \int _0^{1/2} \left( -\log _2 \lambda \right) d\mu _\rho (\lambda ) = {{\,\textrm{Tr}\,}}\left[ \rho \left( -\log _2 L\right) \right] . \end{aligned}$$
  On the other hand, clearly ${{\,\textrm{Tr}\,}}[\sigma L_\delta ] = {{\,\textrm{Tr}\,}}[\sigma L] + \delta$ converges to ${{\,\textrm{Tr}\,}}[\sigma L]>0$ as $\delta \rightarrow 0^+$. This proves (44), and thus allows us to conclude that the optimizations in (23)–(28) all coincide.
4. We now show that the variational program in (26) actually yields the measured relative entropy $D^M\!(\rho \Vert \sigma )$. To begin, we prove that in (26) we can restrict *L* to be of the form $L=I+R$, with ${{\,\textrm{rk}\,}}R<\infty$, without changing the value of the supremum. To this end, pick *L* such that $1/m\le L\le m$ for some $m>0$, and consider an arbitrary $\epsilon >0$. Construct a finite-dimensional projector *P* such that $\left\| \rho - P\rho P\right\| _1,\, \left\| \sigma - P \sigma P\right\| _1 \le \epsilon$. Then,
  $$\begin{aligned}&{{\,\textrm{Tr}\,}}\left[ \rho \log _2 L\right] + \log _2(e) \left( 1 - {{\,\textrm{Tr}\,}}\left[ \sigma L\right] \right) \\&\quad {\mathop {\le }\limits ^{{\text{1 }}}} {{\,\textrm{Tr}\,}}\left[ P\rho P \log _2 L\right] + \log _2(e) \left( 1 - {{\,\textrm{Tr}\,}}\left[ P \sigma P L\right] \right) + \epsilon (\log _2 m + m \log _2(e)) \\&\quad {\mathop {\le }\limits ^{{\text{2 }}}} {{\,\textrm{Tr}\,}}\left[ \rho \log _2 (PLP + \mathbb {1}- P)\right] + \log _2(e) \left( 1 - {{\,\textrm{Tr}\,}}\left[ P \sigma P L\right] \right) + \epsilon (\log _2 m + m \log _2(e)) \\&\quad {\mathop {\le }\limits ^{{\text{3 }}}} {{\,\textrm{Tr}\,}}\left[ \rho \log _2 (PLP + \mathbb {1}- P)\right] + \log _2(e) \left( 1 - {{\,\textrm{Tr}\,}}\left[ \sigma (PLP + \mathbb {1}- P) \right] \right) \\&\qquad + \epsilon (\log _2 m + (m+1) \log _2(e))\, . \end{aligned}$$
  Here, 1 follows because $\Vert \log _2 L\Vert _\infty \le \log _2 m$ and $\Vert L\Vert _\infty \le m$ (where $\Vert \cdot \Vert _\infty$ is the operator norm), in 2 we applied the operator Jensen inequality [118] to the operator-concave function $\log _2$, and 3 is an application of the estimate ${{\,\textrm{Tr}\,}}[\sigma (\mathbb {1}- P)] = {{\,\textrm{Tr}\,}}[\sigma - P\sigma P]\le \left\| \sigma - P \sigma P\right\| _1\le \epsilon$. We see that up to introducing an arbitrarily small error we can substitute $L\mapsto PLP + \mathbb {1}- P = \mathbb {1}+R$, where ${{\,\textrm{rk}\,}}R \le {{\,\textrm{rk}\,}}P<\infty$.
  Now, let *R* be of finite rank, and denote with $R=\sum _{n=1}^N \lambda _n P_n$ its spectral decomposition. Then $L= \mathbb {1}+ R = \sum _{n=0}^N (1+\lambda _n) P_n$, where $P_0 :=\mathbb {1}- \sum _{n=1}^N P_n$ and $\lambda _0=0$, and consequently
  $$\begin{aligned}&{{\,\textrm{Tr}\,}}[\rho \log _2 L] + \log _2(e) \left( 1 - {{\,\textrm{Tr}\,}}[\sigma L]\right) \\&\quad = \log _2(e) (1-{{\,\textrm{Tr}\,}}[\sigma ]) + \sum _{n=0}^N \left( \log _2(1+\lambda _n){{\,\textrm{Tr}\,}}[\rho P_n] - \log _2(e) \lambda _n{{\,\textrm{Tr}\,}}[\sigma P_n]\right) \\&\quad {\mathop {\le }\limits ^{{\text{4 }}}} \log _2(e) (1-{{\,\textrm{Tr}\,}}[\sigma ]) + \sum _{n=1}^N \left( {{\,\textrm{Tr}\,}}[\rho P_n] \log _2 \frac{{{\,\textrm{Tr}\,}}[\rho P_n]}{{{\,\textrm{Tr}\,}}[\sigma P_n]} - \log _2(e)\left( {{\,\textrm{Tr}\,}}[\rho P_n] - {{\,\textrm{Tr}\,}}[\sigma P_n]\right) \right) \\&\quad {\mathop {\le }\limits ^{{\text{5 }}}} \log _2(e) (1-{{\,\textrm{Tr}\,}}[\sigma ]) + \sum _{n=0}^N \left( {{\,\textrm{Tr}\,}}[\rho P_n] \log _2 \frac{{{\,\textrm{Tr}\,}}[\rho P_n]}{{{\,\textrm{Tr}\,}}[\sigma P_n]} - \log _2(e)\left( {{\,\textrm{Tr}\,}}[\rho P_n] - {{\,\textrm{Tr}\,}}[\sigma P_n]\right) \right) \\&\quad = \sum _{n=0}^N {{\,\textrm{Tr}\,}}[\rho P_n] \log _2 \frac{{{\,\textrm{Tr}\,}}[\rho P_n]}{{{\,\textrm{Tr}\,}}[\sigma P_n]} \\&\quad {\mathop {=}\limits ^{{\text{6 }}}} D_{K\! L}\!\left( P^\mathcal {M}_\rho \big \Vert P^\mathcal {M}_\sigma \right) \\&\quad \le D^M\!(\rho \Vert \sigma ) . \end{aligned}$$
  Here, the inequality in 4 comes from the estimate $a \log _2 (1+x) - \log _2(e) b x \le a \log _2 \frac{a}{b} - \log _2(e) (a - b)$, (which can be proven simply by maximisation in *x*), while 5 is a consequence of the fact that $a \log _2 \frac{a}{b} - \log _2(e) (a - b)\ge 0$ for all $a,b\ge 0$.
  In 6, we introduced the measurement $\mathcal {M}:=\{P_x\}_{x\in \{0,\ldots , N\}}$.
  The converse is proved with exactly the same argument put forth by Berta et al. in the proof of [53, Lemma 1]. Namely, let $\mathcal {M}=\{E_x\}_{x\in \mathcal {X}}$ be a quantum measurement. If there exists $x\in \mathcal {X}$ such that ${{\,\textrm{Tr}\,}}[\sigma E_x]=0< {{\,\textrm{Tr}\,}}[\rho E_x]$, then on the one hand clearly $D^M\!(\rho \Vert \sigma ) \ge D_{K\! L}\!\left( P^\mathcal {M}_\rho \big \Vert P^\mathcal {M}_\sigma \right) = +\infty$. On the other, we see that the kernels of $\rho$ and $\sigma$ obey $\ker (\sigma )\nsubseteq \ker (\rho )$, i.e., there exists a pure state $\vert \psi \rangle \in \ker (\sigma )\setminus \ker (\rho )$. Setting $L=\lambda \psi + \mathbb {1}-\psi$ and letting $\lambda \rightarrow +\infty$ proves that the variational program in (26) is unbounded from above, as it should be.
  We now consider the case where ${{\,\textrm{Tr}\,}}[\sigma E_x]=0$ only when also ${{\,\textrm{Tr}\,}}[\rho E_x]=0$. Introduce the set
  $$\begin{aligned} {\widetilde{\mathcal {X}}}:=\{x\in \mathcal {X}: {{\,\textrm{Tr}\,}}[\rho E_x]{{\,\textrm{Tr}\,}}[\sigma E_x]>0 \}\,, \end{aligned}$$
  and write:
  $$\begin{aligned} D_{K\! L}\!\left( P^\mathcal {M}_\rho \big \Vert P^\mathcal {M}_\sigma \right) =&\sum _{x\in {\widetilde{\mathcal {X}}}} {{\,\textrm{Tr}\,}}[\rho E_x] \left( \log _2 {{\,\textrm{Tr}\,}}[\rho E_x] - \log _2 {{\,\textrm{Tr}\,}}[\sigma E_x] \right) \\ =&{{\,\textrm{Tr}\,}}\left[ \rho \sum \nolimits _{x\in {\widetilde{\mathcal {X}}}} \sqrt{E_x}\, \log _2 \left( \frac{{{\,\textrm{Tr}\,}}[\rho E_x]}{{{\,\textrm{Tr}\,}}[\sigma E_x]}\cdot \mathbb {1}\right) \sqrt{E_x} \right] \\ {\mathop {\le }\limits ^{{\text{7 }}}}&{{\,\textrm{Tr}\,}}\left[ \rho \log _2 \left( \sum \nolimits _{x\in {\widetilde{\mathcal {X}}}} \frac{{{\,\textrm{Tr}\,}}[\rho E_x]}{{{\,\textrm{Tr}\,}}[\sigma E_x]}\, E_x\right) \right] \\ {\mathop {=}\limits ^{{\text{8 }}}}&{{\,\textrm{Tr}\,}}\left[ \rho \log _2 L \right] + \log _2(e)\left( 1 - {{\,\textrm{Tr}\,}}[\sigma L]\right) , \end{aligned}$$
  where 7 is again an application of the operator Jensen inequality [118] to the operator-concave function $\log _2$, and in 8 we defined $L:=\sum _x \frac{{{\,\textrm{Tr}\,}}[\rho E_x]}{{{\,\textrm{Tr}\,}}[\sigma E_x]}\, E_x$, so that ${{\,\textrm{Tr}\,}}[\sigma L]=1$.

$$\square $$

#### Remark 32

The programs (24), (26), and (28) are all well defined also for $\sigma =0$. They yield $D^M\!(\rho \Vert 0)=+\infty$, as it should be.

### The monotone ${\Gamma }$

In order to arrive at a proof of Theorem 19, we first formalize the definition of the quantity that appears on the right-hand side of (22).

#### Definition 33

For an arbitrary *m*-mode state $\rho$, let us construct the quantity

45 $$\begin{aligned} \Gamma (\rho ) :=&\ \sup _{h\in \mathcal {B}_{\textrm{sa}}(\mathcal {H}_{m})} \left\{ {{\,\textrm{Tr}\,}}[\rho h] - \log _2 \sup _{\alpha \in \mathbb {C}^m} \langle \alpha |2^h|\alpha \rangle \right\} \end{aligned}$$

46 $$\begin{aligned} =&\sup _{h\in \mathcal {B}_{\textrm{sa}}(\mathcal {H}_{m})} \left\{ {{\,\textrm{Tr}\,}}[\rho h] + \log _2(e) \left( 1- \sup _{\alpha \in \mathbb {C}^m} \langle \alpha |2^h|\alpha \rangle \right) \right\} \end{aligned}$$

Note that since $2^h>0$, there must exist some $\alpha \in \mathbb {C}^m$ such that $\langle \alpha |2^h|\alpha \rangle >0$. Moreover, the two programs in (45) and (46) are equivalent, as can be verified by following the same strategy as in step 1 of the proof of Lemma 20. This ensures that $\Gamma$ is indeed well defined. Let us now establish some of its basic properties.

#### Lemma 34

For an *m*-mode state $\rho$, we have that

47 $$\begin{aligned} \Gamma (\rho )&= \sup _{0<\delta \mathbb {1}<L\in \mathcal {B}_{\textrm{sa}}(\mathcal {H}_{m})} \left\{ {{\,\textrm{Tr}\,}}\left[ \rho \log _2 L\right] - \log _2 \sup _{\alpha \in \mathbb {C}^m} \langle \alpha |L|\alpha \rangle \right\} \end{aligned}$$

48 $$\begin{aligned}&= \sup _{0<\delta \mathbb {1}<L\in \mathcal {B}_{\textrm{sa}}(\mathcal {H}_{m})} \left\{ {{\,\textrm{Tr}\,}}\left[ \rho \log _2 L\right] + \log _2 (e) \left( 1 - \sup _{\alpha \in \mathbb {C}^m} \langle \alpha |L|\alpha \rangle \right) \right\} \end{aligned}$$

49 $$\begin{aligned}&= \sup _{0<L\in \mathcal {B}_{\textrm{sa}}(\mathcal {H}_{m})} \left\{ {{\,\textrm{Tr}\,}}\left[ \rho \log _2 L\right] - \log _2 \sup _{\alpha \in \mathbb {C}^m} \langle \alpha |L|\alpha \rangle \right\} \end{aligned}$$

50 $$\begin{aligned}&= \sup _{0<L\in \mathcal {B}_{\textrm{sa}}(\mathcal {H}_{m})} \left\{ {{\,\textrm{Tr}\,}}\left[ \rho \log _2 L\right] + \log _2 (e) \left( 1 - \sup _{\alpha \in \mathbb {C}^m} \langle \alpha |L|\alpha \rangle \right) \right\} . \end{aligned}$$

#### Proof

The argument proceeds exactly as in steps 1–3 of the proof of Lemma 20. $\square$

We deduce the following elementary but important properties of the function $\Gamma$.

#### Proposition 35

The function $\Gamma$ in Definition 33 is a convex, lower semicontinuous, strongly superadditive nonclassicality monotone. It holds that $\Gamma (\rho ) \le N^M_r\!(\rho )$ for all states $\rho$.

#### Proof

First of all, $\Gamma$ is convex and lower semicontinuous because it is the pointwise supremum of convex-linear and lower semicontinuous functions $\rho \mapsto {{\,\textrm{Tr}\,}}[\rho h] - \log _2 \sup _{\alpha \in \mathbb {C}^m} \langle \alpha |2^h|\alpha \rangle$ (cf. Definition 33). To see that it is a nonclassicality monotone, consider $\rho \in \mathcal {D}(\mathcal {H}_{m})$ and a classical channel $\Lambda :\mathcal {T}_{\textrm{sa}}(\mathcal {H}_{m})\rightarrow \mathcal {T}_{\textrm{sa}}(\mathcal {H}_{m'})$, and write

51 $$\begin{aligned} \Gamma \left( \Lambda (\rho )\right)&= \sup _{0<L'\in \mathcal {B}_{\textrm{sa}}(\mathcal {H}_{m'})} \left\{ {{\,\textrm{Tr}\,}}\left[ \Lambda (\rho ) \log _2 L'\right] - \log _2 \sup _{\alpha \in \mathbb {C}^{m'}} \langle \alpha |L'|\alpha \rangle \right\} \nonumber \\&{\mathop {=}\limits ^{{\text{1 }}}} \sup _{0<L'\in \mathcal {B}_{\textrm{sa}}(\mathcal {H}_{m'})} \left\{ {{\,\textrm{Tr}\,}}\left[ \rho \, \Lambda ^\dag \left( \log _2 L'\right) \right] - \log _2 \sup _{\sigma '\in \mathcal {C}_{m'}} {{\,\textrm{Tr}\,}}[\sigma ' L'] \right\} \nonumber \\&{\mathop {\le }\limits ^{{\text{2 }}}} \sup _{0<L'\in \mathcal {B}_{\textrm{sa}}(\mathcal {H}_{m'})} \left\{ {{\,\textrm{Tr}\,}}\left[ \rho \log _2 \Lambda ^\dag \left( L'\right) \right] - \log _2 \sup _{\sigma '\in \mathcal {C}_{m'}} {{\,\textrm{Tr}\,}}[\sigma ' L'] \right\} \nonumber \\&{\mathop {\le }\limits ^{{\text{3 }}}} \sup _{0<L'\in \mathcal {B}_{\textrm{sa}}(\mathcal {H}_{m'})} \left\{ {{\,\textrm{Tr}\,}}\left[ \rho \log _2 \Lambda ^\dag \left( L'\right) \right] - \log _2 \sup _{\sigma \in \mathcal {C}_{m}} {{\,\textrm{Tr}\,}}[\Lambda (\sigma ) L'] \right\} \nonumber \\&= \sup _{0<L'\in \mathcal {B}_{\textrm{sa}}(\mathcal {H}_{m'})} \left\{ {{\,\textrm{Tr}\,}}\left[ \rho \log _2 \Lambda ^\dag \left( L'\right) \right] - \log _2 \sup _{\sigma \in \mathcal {C}_{m}} {{\,\textrm{Tr}\,}}[\sigma \Lambda ^\dag (L')] \right\} \nonumber \\&{\mathop {\le }\limits ^{{\text{4 }}}} \sup _{0<L\in \mathcal {B}_{\textrm{sa}}(\mathcal {H}_{m})} \left\{ {{\,\textrm{Tr}\,}}\left[ \rho \log _2 L \right] - \log _2 \sup _{\sigma \in \mathcal {C}_{m}} {{\,\textrm{Tr}\,}}[\sigma L] \right\} \nonumber \\&= \Gamma (\rho )\, . \end{aligned}$$

The justification of the above derivation is as follows. 1: We used the definition of adjoint map, and observed that since $L'$ is bounded and $\mathcal {C}_{m'}={\overline{{{\,\textrm{conv}\,}}}}\left\{ \vert \alpha \rangle \!\langle \alpha \vert :\, \alpha \in \mathbb {C}^{m'}\right\}$, it holds that $\sup _{\sigma '\in \mathcal {C}_{m'}} {{\,\textrm{Tr}\,}}[\sigma ' L'] = \sup _{\alpha \in \mathbb {C}^{m'}} \langle \alpha |L'|\alpha \rangle$. 2: We applied the operator Jensen inequality [118] to the operator-concave function $\log _2$. 3: We restricted the inner supremum over $\sigma '$ to classical states of the form $\sigma '=\Lambda (\sigma )$, with $\sigma \in \mathcal {C}_m$. 4: We observed that if $0<L'\in \mathcal {B}_{\textrm{sa}}(\mathcal {H}_{m'})$ then also $0<\Lambda ^\dag (L')\in \mathcal {B}_{\textrm{sa}}(\mathcal {H}_{m})$, which can be seen by noticing that ${{\,\textrm{Tr}\,}}\left[ \omega \, \Lambda ^\dag (L')\right] ={{\,\textrm{Tr}\,}}\left[ \Lambda (\omega ) L'\right] >0$ for all states $\omega \in \mathcal {D}(\mathcal {H}_{m})$.

We now prove that $\Gamma$ is strongly superadditive. To this end, we take an arbitrary $(m+n)$-mode state $\rho _{AB}$ and write

$$\begin{aligned}&\Gamma \left( \rho _{AB}\right) \\&\quad = \sup _{0<L_{AB}\in \mathcal {B}_{\textrm{sa}}(\mathcal {H}_{m+n})} \left\{ {{\,\textrm{Tr}\,}}\left[ \rho _{AB} \log _2 L_{AB}\right] - \log _2 \sup _{\alpha _A \in \mathbb {C}^m\!\!,\; \alpha _B \in \mathbb {C}^n } \left( \langle \alpha _A\vert \otimes \langle \alpha _B\vert \right) {L_{AB}}\left( \vert \alpha _A\rangle \otimes \vert \alpha _B\rangle \right) \right\} \\&\quad {\mathop {\ge }\limits ^{{\text{5 }}}} \sup _{\begin{array}{c} 0<L_A\in \mathcal {B}_{\textrm{sa}}(\mathcal {H}_{m}),\\ 0<L_B\in \mathcal {B}_{\textrm{sa}}(\mathcal {H}_{n}) \end{array}} \left\{ {{\,\textrm{Tr}\,}}\left[ \rho _{AB} \log _2 (L_A \otimes L_B)\right] - \log _2 \sup _{\alpha _A \in \mathbb {C}^m\!\!,\; \alpha _B \in \mathbb {C}^n} \left( \langle \alpha _A\vert \otimes \langle \alpha _B\vert \right) {(L_A\otimes L_B)}\left( \vert \alpha _A\rangle \otimes \vert \alpha _B\rangle \right) \right\} \\&\quad = \sup _{\begin{array}{c} 0<L_A\in \mathcal {B}_{\textrm{sa}}(\mathcal {H}_{m}),\\ 0<L_B\in \mathcal {B}_{\textrm{sa}}(\mathcal {H}_{n}) \end{array}} \left\{ {{\,\textrm{Tr}\,}}\left[ \rho _A \log _2 L_A\right] + {{\,\textrm{Tr}\,}}\left[ \rho _B \log _2 L_B\right] - \log _2 \left[ \left( \sup _{\alpha _A\in \mathbb {C}^{m}} \langle \alpha _A|L_A|\alpha _A\rangle \right) \left( \sup _{\alpha _B\in \mathbb {C}^{n}} \langle \alpha _B|L_B|\alpha _B\rangle \right) \right] \right\} \\&\quad = \sup _{0<L_{A}\in \mathcal {B}_{\textrm{sa}}(\mathcal {H}_{m})} \left\{ {{\,\textrm{Tr}\,}}\left[ \rho _{A} \log _2 L_{A}\right] - \log _2 \sup _{\alpha _{\!A}\in \mathbb {C}^{m}} \langle \alpha _{A}|L_{A}|\alpha _{A}\rangle \right\} \\&\qquad + \sup _{0<L_{B}\in \mathcal {B}_{\textrm{sa}}(\mathcal {H}_{n})} \left\{ {{\,\textrm{Tr}\,}}\left[ \rho _{B} \log _2 L_{B}\right] - \log _2 \sup _{\alpha _{B}\in \mathbb {C}^{n}} \langle \alpha _{B}|L_{B}|\alpha _{B}\rangle \right\} \\&\quad = \Gamma (\rho _A)+\Gamma (\rho _B)\, , \end{aligned}$$

where in 5 we restricted the supremum to product operators $L_{AB}=L_A \otimes L_B$. It remains to establish the inequality $\Gamma \le N^M_r\!$. This is done as follows:

$$\begin{aligned} N^M_r\!(\rho )&= \inf _{\sigma \in \mathcal {C}_m} D^M\!(\rho \Vert \sigma ) \\&{\mathop {=}\limits ^{{\text{6 }}}} \inf _{\sigma \in \mathcal {C}_m} \sup _{0<L\in \mathcal {B}_{\textrm{sa}}(\mathcal {H}_{m})} \left\{ {{\,\textrm{Tr}\,}}\left[ \rho \log _2 L\right] - \log _2 {{\,\textrm{Tr}\,}}\left[ \sigma L\right] \right\} \\&{\mathop {\ge }\limits ^{{\text{7 }}}} \sup _{0<L\in \mathcal {B}_{\textrm{sa}}(\mathcal {H}_{m})} \inf _{\sigma \in \mathcal {C}_m} \left\{ {{\,\textrm{Tr}\,}}[\rho \log _2 L] - \log _2 {{\,\textrm{Tr}\,}}[\sigma L] \right\} \\&= \sup _{0<L\in \mathcal {B}_{\textrm{sa}}(\mathcal {H}_{m})} \left\{ {{\,\textrm{Tr}\,}}[\rho \log _2 L] - \log _2 \sup _{\sigma \in \mathcal {C}_m} {{\,\textrm{Tr}\,}}[\sigma L] \right\} \\&{\mathop {=}\limits ^{{\text{8 }}}} \sup _{0<L\in \mathcal {B}_{\textrm{sa}}(\mathcal {H}_{m})} \left\{ {{\,\textrm{Tr}\,}}[\rho \log _2 L] - \log _2 \sup _{\alpha \in \mathbb {C}^m} \langle \alpha |L|\alpha \rangle \right\} \,. \end{aligned}$$

Here, in 6 we employed the variational representation (27) for the measured relative entropy, in 7 we remembered that

$$\begin{aligned} \inf _{x\in X} \sup _{y\in Y} f(x,y) \ge \sup _{y\in Y} \inf _{x\in X} f(x,y) \end{aligned}$$

holds for an arbitrary function $f:X\times Y\rightarrow \mathbb {R}$ on any product set $X\times Y$, and finally in 8 we noted that since $\mathcal {C}_m={\overline{{{\,\textrm{conv}\,}}}}\left\{ \vert \alpha \rangle \!\langle \alpha \vert :\, \alpha \in \mathbb {C}^m\right\}$ and the function $\sigma \mapsto {{\,\textrm{Tr}\,}}[\sigma L]$ is linear and trace-norm continuous (because *L* is bounded), it achieves the maximum on the extreme points of $\mathcal {C}_m$, i.e., on coherent states. $\square$

#### Corollary 36

The regularization $\Gamma ^\infty (\rho ):=\lim _{n\rightarrow \infty } \frac{1}{n}\, \Gamma (\rho ^{\otimes n})$ exists and is unique for all states $\rho$. It is a lower semicontinuous, weakly additive, and strongly superadditive nonclassicality monotone, and it satisfies that $\Gamma ^\infty \ge \Gamma$.

#### Proof

Follows by combining Lemma 26 and Proposition 35. $\square$

### Two more technical lemmata

In order to prove Theorem 19, and from there deduce Theorem 23, we need two more technical lemmata. The first one tells us that provided a state $\rho$ has finite entropy, which will most definitely be the case in all situations of physical interest, we can take the operator *L* in the variational program for $N^M_r\!$ to be not only bounded but also trace class.

#### Lemma 37

On an *m*-mode system, let

52 $$\begin{aligned} {\widetilde{\mathcal {C}}}_m :={{\,\textrm{conv}\,}}\left( \mathcal {C}_m \cup \{0\}\right) \end{aligned}$$

denote the set of subnormalized classical states. Then, the measured relative entropy of nonclassicality admits the variational expressions

53 $$\begin{aligned} N^M_r\!(\rho )&= \inf _{\sigma \in \mathcal {C}_m}\sup _{\begin{array}{c} 0<L\in \mathcal {B}_{\textrm{sa}}(\mathcal {H}_{m}) \end{array}}\left\{ {{\,\textrm{Tr}\,}}[\rho \log _2 L] + \log _2(e) \left( 1 - {{\,\textrm{Tr}\,}}[\sigma L] \right) \right\} \end{aligned}$$

54 $$\begin{aligned}&=\inf _{\sigma \in {\widetilde{\mathcal {C}}}_m}\sup _{\begin{array}{c} 0<L\in \mathcal {B}_{\textrm{sa}}(\mathcal {H}_{m}) \end{array}}\left\{ {{\,\textrm{Tr}\,}}[\rho \log _2 L] + \log _2(e) \left( 1 - {{\,\textrm{Tr}\,}}[\sigma L] \right) \right\} \end{aligned}$$

for all *m*-mode states $\rho$. Moreover, in both (53) and (54):

(i) If $S(\rho )<\infty$, we can assume that $L\in \mathcal {T}_{\textrm{sa}}(\mathcal {H}_{m})$ is of trace class, and that $-{{\,\textrm{Tr}\,}}\rho \log _2 L <\infty$;
(ii) If ${{\,\textrm{rk}\,}}\rho <\infty$, we can assume that ${{\,\textrm{supp}\,}}L = {{\,\textrm{supp}\,}}\rho$ and hence ${{\,\textrm{rk}\,}}L<\infty$, with the convention that $-{{\,\textrm{Tr}\,}}\rho \log _2 L$ is computed on the common support of $\rho$ and *L*.

#### Proof

As we have already seen, the expression (53) is obtained by plugging (28) into the definition (21) of measured relative entropy of nonclassicality. To see that also (54) holds, just notice that

$$\begin{aligned} N^M_r\!(\rho )&= \inf _{\sigma \in \mathcal {C}_m} D^M\!(\rho \Vert \sigma ) \\&= \inf _{\sigma \in \mathcal {C}_m,\, \lambda \in [0,1]} \left\{ D^M\!(\rho \Vert \sigma ) - \log _2 \lambda \right\} \\&= \inf _{\sigma \in \mathcal {C}_m,\, \lambda \in [0,1]} D^M\!(\rho \Vert \lambda \sigma ) \\&= \inf _{\sigma \in {\widetilde{\mathcal {C}}}_m} D^M\!(\rho \Vert \sigma ) \\&= \inf _{\sigma \in {\widetilde{\mathcal {C}}}_m} \sup _{0<L\in \mathcal {B}_{\textrm{sa}}(\mathcal {H}_{m})} \left\{ {{\,\textrm{Tr}\,}}[\rho \log _2 L] + \log _2(e) \left( 1 - {{\,\textrm{Tr}\,}}[\sigma L] \right) \right\} , \end{aligned}$$

where the last step is once again (28). We now prove claims (i) and (ii) for (53).

We start by observing that restricting the set of operators *L* over which we optimize can only decrease the final value of the program. Thus, it suffices to establish the opposite inequality. We start from claim (i). Let $\rho$ be a finite-entropy *m*-mode state with spectral decomposition $\rho =\sum _{k=0}^\infty p_k \vert e_k\rangle \!\langle e_k\vert$. We can assume without loss of generality that ${\overline{{{\,\textrm{span}\,}}}}\{e_k\}_{k\in \mathbb {N}}=\mathcal {H}_m$, i.e., that $\{e_k\}_{k\in \mathbb {N}}$ forms a basis of the entire Hilbert space. Pick a bounded but not necessarily trace class operator *L* that can enter the expression (53). Without loss of generality, we can assume that

55 $$\begin{aligned} -\infty< {{\,\textrm{Tr}\,}}[\rho \log _2 L] = \sum _{k=0}^\infty p_k \langle e_k|\log _2 L|e_k\rangle < +\infty \, . \end{aligned}$$

In fact, if this is not the case the objective function evaluates to $-\infty$.

For a certain $n\in \mathbb {N}$, construct the completely positive unital map $\Pi _n:\mathcal {B}_{\textrm{sa}}(\mathcal {H}_{m})\rightarrow \mathcal {B}_{\textrm{sa}}(\mathcal {H}_{m})$ given by $\Pi (X):=P_nXP_n+Q_nXQ_n$, where $P_n:=\sum _{k=0}^{n-1}\vert e_k\rangle \!\langle e_k\vert$ is the projector onto the the linear span ${{\,\textrm{span}\,}}\{\vert e_k\rangle \}_{k=0,\ldots , n-1}$ of the first *n* eigenvectors of $\rho$, and $Q_n:=\mathbb {1}-P_n = \sum _{k=n}^\infty \vert e_k\rangle \!\langle e_k\vert$. Set $\rho = \rho _n + \delta _n$, with $\rho _n:=P_n \rho P_n$ and $\delta _n :=Q_n \rho Q_n$, and define the trace class operator $L_n:=P_n L P_n + \delta _n$. Then, we have that

56 $$\begin{aligned} {{\,\textrm{Tr}\,}}[\rho \log _2 L]&= {{\,\textrm{Tr}\,}}[\rho _n \log _2 L] + {{\,\textrm{Tr}\,}}[\delta _n \log _2 L] \nonumber \\&{\mathop {=}\limits ^{{\text{1 }}}} {{\,\textrm{Tr}\,}}[\Pi (\rho _n) \log _2 L] + {{\,\textrm{Tr}\,}}[\delta _n \log _2 L] \nonumber \\&{\mathop {=}\limits ^{{\text{2 }}}} {{\,\textrm{Tr}\,}}[\rho _n\, \Pi \left( \log _2 L\right) ] + {{\,\textrm{Tr}\,}}[\delta _n \log _2 L] \nonumber \\&{\mathop {\le }\limits ^{{\text{3 }}}} {{\,\textrm{Tr}\,}}[\rho _n \log _2 \Pi (L)] + {{\,\textrm{Tr}\,}}[\delta _n \log _2 L] \nonumber \\&{\mathop {=}\limits ^{{\text{4 }}}} {{\,\textrm{Tr}\,}}[\rho _n \log _2 (P_n L P_n + \delta _n)] + {{\,\textrm{Tr}\,}}[\delta _n \log _2 L] \nonumber \\&= {{\,\textrm{Tr}\,}}[\rho \log _2 L_n] - {{\,\textrm{Tr}\,}}[\delta _n \log _2 \delta _n] + {{\,\textrm{Tr}\,}}[\delta _n \log _2 L]\, . \end{aligned}$$

Here, in 1 we observed that $\rho _n=\Pi (\rho _n)$, in 2 we used the easily verified fact that $\Pi =\Pi ^\dag$, in 3 we applied the operator Jensen inequality [118], and finally in 4 we changed the component of the argument of the first logarithm on the subspace ${{\,\textrm{supp}\,}}Q_n$, which is irrelevant because the trace is against $\rho _n$, whose support is orthogonal to that of $Q_n$. Now, since $S(\rho ) = -{{\,\textrm{Tr}\,}}[\rho \log _2 \rho ] = \sum _{k=0}^\infty p_k \log _2\frac{1}{p_k} < \infty$, we see that

57 $$\begin{aligned} \lim _{n\rightarrow \infty } \left( - {{\,\textrm{Tr}\,}}[\delta _n \log _2 \delta _n] \right) = \lim _{n\rightarrow \infty } \sum _{k=n}^\infty p_k \log _2\frac{1}{p_k} = 0\, . \end{aligned}$$

Moreover, (55) implies that

58 $$\begin{aligned} \lim _{n\rightarrow \infty } {{\,\textrm{Tr}\,}}[\delta _n \log _2 L] = \lim _{n\rightarrow \infty } \sum _{k=n}^\infty p_k \langle e_k|\log _2 L|e_k\rangle = 0\, . \end{aligned}$$

Putting (56)–(58) together, we see that

59 $$\begin{aligned} \liminf _{n\rightarrow \infty } {{\,\textrm{Tr}\,}}[\rho \log _2 L_n] \ge {{\,\textrm{Tr}\,}}[\rho \log _2 L]\, . \end{aligned}$$

On the other hand, since ${\overline{{{\,\textrm{span}\,}}}}\{\vert e_k\rangle \}_{k\in \mathbb {N}} = \mathcal {H}_m$, we have that $\lim _{n\rightarrow \infty } {{\,\textrm{Tr}\,}}[\sigma P_n] = 1$ and therefore, by the gentle measurement lemma [37, 38] (see also [119, Lemma 9.4.2]),

60 $$\begin{aligned} \lim _{n\rightarrow \infty } \left\| \sigma - P_n \sigma P_n\right\| _1 = 0\, . \end{aligned}$$

This immediately implies that

61 $$\begin{aligned} \liminf _{n\rightarrow \infty } \left( 1 - {{\,\textrm{Tr}\,}}[\sigma L_n] \right)&= \liminf _{n\rightarrow \infty } \left( 1 - {{\,\textrm{Tr}\,}}[P_n\sigma P_n L] - {{\,\textrm{Tr}\,}}[\sigma \delta _n] \right) \nonumber \\&{\mathop {\ge }\limits ^{{\text{5 }}}} \liminf _{n\rightarrow \infty } \left( 1 - {{\,\textrm{Tr}\,}}[\sigma L] - \left\| \sigma - P_n\sigma P_n\right\| _1 \Vert L\Vert _\infty - {{\,\textrm{Tr}\,}}[\delta _n] \right) \nonumber \\&{\mathop {=}\limits ^{{\text{6 }}}} 1 - {{\,\textrm{Tr}\,}}[\sigma L]\, . \end{aligned}$$

Here, 5 comes from the fact that *L* is bounded and also that $\sigma \le \mathbb {1}$, while 6 descends from (60) and from the elementary observation that since ${{\,\textrm{Tr}\,}}[\rho ] = \sum _{k=0}^\infty p_k = 1$ it follows that $\lim _{n\rightarrow \infty } {{\,\textrm{Tr}\,}}[\delta _n] = \lim _{n\rightarrow \infty } \sum _{k=n}^\infty p_k = 0$.

Finally, combining (59) and (61) we deduce that

$$\begin{aligned}&\liminf _{n\rightarrow \infty } \left( {{\,\textrm{Tr}\,}}[\rho \log _2 L_n] + \log _2(e) \left( 1 - {{\,\textrm{Tr}\,}}[\sigma L_n] \right) \right) \\&\quad \ge \liminf _{n\rightarrow \infty } {{\,\textrm{Tr}\,}}[\rho \log _2 L_n] + \log _2(e) \liminf _{n\rightarrow \infty } \left( 1 - {{\,\textrm{Tr}\,}}[\sigma L_n] \right) \\&\quad \ge {{\,\textrm{Tr}\,}}[\rho \log _2 L] + \log _2(e) \left( 1- {{\,\textrm{Tr}\,}}[\sigma L]\right) . \end{aligned}$$

Remembering that $L_n$ is a trace class operator, this in turn implies that

$$\begin{aligned}{} & {} \sup _{\begin{array}{c} 0<L\in \mathcal {B}_{\textrm{sa}}(\mathcal {H}_{m}) \end{array}}\left\{ {{\,\textrm{Tr}\,}}[\rho \log _2 L] + \log _2(e) \left( 1- {{\,\textrm{Tr}\,}}[\sigma L]\right) \right\} \\{} & {} \qquad \quad \qquad \le \sup _{\begin{array}{c} 0<L\in \mathcal {T}_{\textrm{sa}}(\mathcal {H}_{m}) \end{array}}\left\{ {{\,\textrm{Tr}\,}}[\rho \log _2 L] + \log _2(e) \left( 1- {{\,\textrm{Tr}\,}}[\sigma L]\right) \right\} , \end{aligned}$$

thus showing that in fact equality holds. The proof of claim (i) is now complete.

As for claim (ii), it suffices to repeat the above reasoning and observe that if ${{\,\textrm{rk}\,}}\rho < \infty$ then $\delta _n=0$ for sufficiently large *n*, thus entailing that ${{\,\textrm{rk}\,}}L_n < \infty$. $\square$

Our second preliminary lemma presents a technical result whose topological content will be indispensable for a careful application of Sion’s minimax theorem to the variational program (53).

#### Lemma 38

The cone

62 $$\begin{aligned} \mathcal {C}_m^+ :=\left\{ \lambda \sigma : \lambda \ge 0,\, \sigma \in \mathcal {C}_m\right\} \subset \mathcal {T}_{\textrm{sa}}^+(\mathcal {H}_{m}) \end{aligned}$$

generated by the set of classical states is closed with respect to the weak* topology on $\mathcal {T}_{\textrm{sa}}(\mathcal {H}_{m})$. Therefore, the set ${\widetilde{\mathcal {C}}}_m = {{\,\textrm{conv}\,}}\left( \mathcal {C}_m \cup \{0\} \right)$ of subnormalized classical states, defined in (52), is weak*-compact.

#### Proof

Remember by Remark 1 that we can think of $\mathcal {T}_{\textrm{sa}}(\mathcal {H}_{m})$ as the dual space to $\mathcal {K}_{\textrm{sa}}(\mathcal {H}_{m})$, the set of compact operators on $\mathcal {H}_m$. We now show that $\mathcal {C}_m^+$ is in fact the dual of a set $\mathcal {S}\subseteq \mathcal {K}_{\textrm{sa}}(\mathcal {H}_{m})$ of compact operators, i.e.,

$$\begin{aligned} \mathcal {C}_m^+=\mathcal {S}^*:=\left\{ T\in \mathcal {T}_{\textrm{sa}}(\mathcal {H}_{m}):\, {{\,\textrm{Tr}\,}}[TK]\ge 0\ \forall \ K\in \mathcal {S}\right\} . \end{aligned}$$

Dual sets turn out to be automatically weak*-closed. This can be seen, e.g., in the case of $\mathcal {S}^*$, by noting that it can be written as the intersection

$$\begin{aligned} \mathcal {S}^* = \bigcap _{K\in \mathcal {S}} \left\{ T\in \mathcal {T}_{\textrm{sa}}(\mathcal {H}_{m}):\, {{\,\textrm{Tr}\,}}[TK]\ge 0\right\} = \bigcap _{K\in \mathcal {S}} \varphi _K^{-1}\left( [0,\infty ) \right) , \end{aligned}$$

where $\varphi _K:\mathcal {T}_{\textrm{sa}}(\mathcal {H}_{m})\rightarrow \mathbb {R}$ is defined by $\varphi _K(T):={{\,\textrm{Tr}\,}}[TK]$. Since the maps $\varphi _K$ are weak*-continuous by definition, each set $\varphi _K^{-1}\left( [0,\infty ) \right)$ is weak*-closed, and therefore so is their intersection $\mathcal {S}^*$.

From now on, for the sake of readability we write everything for single-mode systems only. Set

$$\begin{aligned} \mathcal {S} :=\left\{ \sum _{\mu ,\nu =1}^n\! \psi _\mu ^* \psi _\nu \, e^{\frac{1}{2} |\alpha _\mu -\alpha _\nu |^2}\, \lambda ^{a^\dag \! a} {\mathscr {D}}(\alpha _\mu - \alpha _\nu ) \lambda ^{a^\dag \! a}\! :\, n\in \mathbb {N}_+,\, \psi \in \mathbb {C}^n,\, \alpha \in \mathbb {C}^n,\, \lambda \in [0,1) \right\} , \end{aligned}$$

where ${\mathscr {D}}$ is the displacement operator (3). Note that every operator in $\mathcal {S}$ is a finite linear combination of operators of the form $\lambda ^{a^\dag \! a} {\mathscr {D}}(\alpha _\mu - \alpha _\nu ) \lambda ^{a^\dag \! a}$, which are clearly compact (in fact, even trace class) as long as $\lambda \in [0,1)$. It is also elementary to see that $\vert \beta \rangle \!\langle \beta \vert \in \mathcal {S}^*$ for every $\beta \in \mathbb {C}$, because

$$\begin{aligned}&\langle \beta | \left( \sum _{\mu ,\nu =1}^n\! \psi _\mu ^* \psi _\nu \, e^{\frac{1}{2} |\alpha _\mu -\alpha _\nu |^2}\, \lambda ^{a^\dag \! a} {\mathscr {D}}(\alpha _\mu - \alpha _\nu ) \lambda ^{a^\dag \! a}\right) |\beta \rangle \\&\quad = \sum _{\mu ,\nu =1}^n\! \psi _\mu ^* \psi _\nu \, e^{\frac{1}{2} |\alpha _\mu -\alpha _\nu |^2} \langle \beta | \lambda ^{a^\dag \! a} {\mathscr {D}}(\alpha _\mu - \alpha _\nu ) \lambda ^{a^\dag \! a} |\beta \rangle \\&\quad {\mathop {=}\limits ^{{\text{1 }}}} \sum _{\mu ,\nu =1}^n\! \psi _\mu ^* \psi _\nu \, e^{\frac{1}{2} |\alpha _\mu -\alpha _\nu |^2} e^{-(1-\lambda ^2)|\beta |^2} \langle \lambda \beta | {\mathscr {D}}(\alpha _\mu - \alpha _\nu ) |\lambda \beta \rangle \\&\quad {\mathop {=}\limits ^{{\text{2 }}}} e^{-(1-\lambda ^2)|\beta |^2} \sum _{\mu ,\nu =1}^n\! \psi _\mu ^* \psi _\nu \, e^{\lambda \left( (\alpha _\mu -\alpha _\nu )\beta ^* - (\alpha _\mu -\alpha _\nu )^* \beta \right) } \\&\quad = e^{-(1-\lambda ^2)|\beta |^2} \sum _{\mu ,\nu =1}^n\! \psi _\mu ^*\, e^{\lambda (\alpha _\mu \beta ^* - \alpha _\mu ^* \beta )}\, \psi _\nu \, e^{\lambda (\alpha _\nu ^* \beta - \alpha _\nu \beta ^*)} \\&\quad = e^{-(1-\lambda ^2)|\beta |^2} \left| \sum \nolimits _{\mu =1}^n \psi _\mu ^*\, e^{\lambda (\alpha _\mu \beta ^* - \alpha _\mu ^* \beta )}\right| ^2 \\&\quad \ge 0\, , \end{aligned}$$

where in 1 we used (5) and in 2 the Weyl form (4) of the canonical commutation relations multiple times. Since $\mathcal {S}^*$ is convex and weak*-closed, and hence in particular closed with respect to the trace norm topology, we see that $\mathcal {C}_1 = {\overline{{{\,\textrm{conv}\,}}}}\{\vert \beta \rangle \!\langle \beta \vert :\beta \in \mathbb {C}\}\subseteq \mathcal {S}^*$. Noting that $\mathcal {S}^*$ is a cone, i.e., it is closed under multiplication by nonnegative scalars, we conclude that in fact $\mathcal {C}_1^+ \subseteq \mathcal {S}^*$.

Let us now prove the opposite inclusion, again in the single-mode case. Pick $T\in \mathcal {T}_{\textrm{sa}}(\mathcal {H}_{1})$ such that ${{\,\textrm{Tr}\,}}[TK]\ge 0$ for all $K\in \mathcal {S}$; then

$$\begin{aligned} 0&\le \liminf _{\lambda \rightarrow 1^-} \sum _{\mu ,\nu =1}^n\! \psi _\mu ^* \psi _\nu \, e^{\frac{1}{2} |\alpha _\mu -\alpha _\nu |^2}\, {{\,\textrm{Tr}\,}}\left[ T\, \lambda ^{a^\dag \! a} {\mathscr {D}}(\alpha _\mu - \alpha _\nu )\lambda ^{a^\dag \! a}\right] \\&\le \sum _{\mu ,\nu =1}^n\! \psi _\mu ^* \psi _\nu \, e^{\frac{1}{2} |\alpha _\mu -\alpha _\nu |^2}\, \lim _{\lambda \rightarrow 1^-} {{\,\textrm{Tr}\,}}\left[ T\, \lambda ^{a^\dag \! a} {\mathscr {D}}(\alpha _\mu - \alpha _\nu )\lambda ^{a^\dag \! a}\right] \\&{\mathop {=}\limits ^{{\text{3 }}}} \sum _{\mu ,\nu =1}^n\! \psi _\mu ^* \psi _\nu \, e^{\frac{1}{2} |\alpha _\mu -\alpha _\nu |^2}\, {{\,\textrm{Tr}\,}}\left[ T\, {\mathscr {D}}(\alpha _\mu - \alpha _\nu )\right] \\&= \sum _{\mu ,\nu =1}^n\! \psi _\mu ^* \psi _\nu \, e^{\frac{1}{2} |\alpha _\mu -\alpha _\nu |^2}\, \chi _T(\alpha _\mu - \alpha _\nu ) \end{aligned}$$

for all $\alpha \in \mathbb {C}^n$ and $\psi \in \mathbb {C}^n$, where the function $\chi _T:\mathbb {C}\rightarrow \mathbb {C}$ defined by $\chi _T(\alpha )= {{\,\textrm{Tr}\,}}[T {\mathscr {D}}(\alpha )]$ is the characteristic function (6) of *T*. To prove 3, since ${\mathscr {D}}(\alpha _\mu -\alpha _\nu )$ is bounded (actually, unitary) it suffices to show that $\lim _{\lambda \rightarrow 1^-} \left\| \lambda ^{a^\dag \! a} T \lambda ^{a^\dag \! a} - T\right\| _1=0$ for all trace class *T*. To see this, we decompose $T=T_+ - T_-$ into its positive and negative parts $T_\pm \ge 0$, which are also trace class operators. Note that

$$\begin{aligned} \lim _{\lambda \rightarrow 1^-} {{\,\textrm{Tr}\,}}\left[ \lambda ^{2 a^\dag \! a} T_\pm \right] = \lim _{\lambda \rightarrow 1^-} \sum _{n=0}^\infty \lambda ^{2n} \langle n|T_\pm |n\rangle = \sum _{n=0}^\infty \langle n|T_\pm |n\rangle = {{\,\textrm{Tr}\,}}[T_\pm ] \end{aligned}$$

thanks to Abel’s theorem, and therefore, by the gentle measurement lemma [37, 38] (see also [119, Lemma 9.4.2]),

$$\begin{aligned} \lim _{\lambda \rightarrow 1^-} \left\| T_\pm - \lambda ^{a^\dag \! a}T_\pm \lambda ^{a^\dag \! a}\right\| _1=0\, , \end{aligned}$$

in turn implying that

$$\begin{aligned} \lim _{\lambda \rightarrow 1^-} \left\| \lambda ^{a^\dag \! a} T \lambda ^{a^\dag \! a} - T\right\| _1{} & {} \le \lim _{\lambda \rightarrow 1^-} \left\| \lambda ^{a^\dag \! a} T_+ \lambda ^{a^\dag \! a} - T_+\right\| _1 + \lim _{\lambda \rightarrow 1^-} \left\| \lambda ^{a^\dag \! a} T_- \lambda ^{a^\dag \! a} - T_-\right\| _1 \\{} & {} = 0\, . \end{aligned}$$

We have just established that, for all $\alpha \in \mathbb {C}^n$, the matrix $\left( e^{\frac{1}{2} |\alpha _\mu -\alpha _\nu |^2}\, \chi _T(\alpha _\mu - \alpha _\nu )\right) _{\mu ,\nu =1,\ldots , n}$ is positive semidefinite. This is known [99] to imply that $T=\lambda \sigma$ for some $\lambda \ge 0$ and some classical state $\sigma$, i.e., $T\in \mathcal {C}_m^+$.

This latter claim can be also verified as follows. Applying the classical Bochner theorem, we see that the function $\mathbb {C}\ni \alpha \mapsto \varphi _T(\alpha ):=\chi _T(\alpha )\, e^{\frac{1}{2} |\alpha |^2}$ is the Fourier transform of a positive measure. Since $\varphi _T$ is well-known to be the Fourier transform of the *P*-function [120, Lemma 1], we conclude that the *P*-function of *T* is non-negative, i.e., *T* is a non-negative multiple of a classical state.

We conclude that $\mathcal {C}_1^+=\mathcal {S}^*$, and hence that $\mathcal {C}_1^+$ is weak*-closed. The exact same argument in fact shows that $\mathcal {C}_m^+$ is weak*-closed for any finite number of modes *m*. Since the unit ball $B_m:=\left\{ T\in \mathcal {T}_{\textrm{sa}}(\mathcal {H}_{m}): \Vert T\Vert _1\le 1\right\}$ of $\mathcal {T}_{\textrm{sa}}(\mathcal {H}_{m})=\mathcal {K}_{\textrm{sa}}(\mathcal {H}_{m})^*$ is weak*-compact by the Banach–Alaoglu theorem [121, Thm. 2.6.18],

$$\begin{aligned} {\widetilde{\mathcal {C}}}_m = {{\,\textrm{conv}\,}}\left( \mathcal {C}_m \cup \{0\} \right) = \mathcal {C}_m^+ \cap B_m \end{aligned}$$

is the intersection of a weak*-closed and a weak*-compact set, and hence it is itself weak*-compact. $\square$

### Proof of Theorems 19 and 23

We are finally ready to present our main result about the measured relative entropy of nonclassicality.

#### Proof of Theorem 19

Let us use Lemma 37(i) to write an improved form of (54) as

$$\begin{aligned} N^M_r\!(\rho ) =&\inf _{\sigma \in {\widetilde{\mathcal {C}}}_m} \sup _{\begin{array}{c} 0<L\in \mathcal {T}_{\textrm{sa}}(\mathcal {H}_{m}),\\ -{{\,\textrm{Tr}\,}}\rho \log _2 L <\infty \end{array}} F_\rho (\sigma , L)\, , \\ F_\rho (\sigma , L) :=&\ {{\,\textrm{Tr}\,}}[\rho \log _2 L] + \log _2(e) \left( 1 - {{\,\textrm{Tr}\,}}[\sigma L] \right) . \end{aligned}$$

Now:

(i) ${\widetilde{\mathcal {C}}}_m$ is weak*-compact by Lemma 38, and manifestly convex;
(ii) $\{L\in \mathcal {T}_{\textrm{sa}}(\mathcal {H}_{m}):\, L>0{,\ -{{\,\textrm{Tr}\,}}\rho \log _2 L<\infty }\}$ is convex thanks to the operator concavity of the logarithm;
(iii) $F_\rho (\cdot ,L)$ is a convex (actually, convex-linear) function on ${\widetilde{\mathcal {C}}}_m$ for every fixed $L>0$; by definition of weak* topology it is also weak*-continuous (because *L* is also compact);
(iv) $F_\rho (\sigma ,\cdot )$ is a concave function on $\{L\in \mathcal {T}_{\textrm{sa}}(\mathcal {H}_{m}):\, L>0{,\ -{{\,\textrm{Tr}\,}}\rho \log _2 L<\infty }\}$ for all $\sigma \in {\widetilde{\mathcal {C}}}_m$, because $\log _2$ is operator concave; it is also upper semicontinuous with respect to the trace norm topology, because ${{\,\textrm{Tr}\,}}[\rho \log _2 L]=-S(\rho )-D(\rho \Vert L)$, and $L\mapsto D(\rho \Vert L)$ is lower semicontinuous with respect to the weak topology [45, Corollary 5.12(i)] and hence (Corollary 3) with respect to the trace norm topology, too.

Since all assumptions of Sion’s minimax theorem [106] are satisfied, we can exchange infimum and supremum, and write

$$\begin{aligned} N^M_r\!(\rho )&{\mathop {=}\limits ^{{\text{1 }}}} \sup _{{\begin{array}{c} 0<L\in \mathcal {T}_{\textrm{sa}}(\mathcal {H}_{m}),\\ [.2ex] -{{\,\textrm{Tr}\,}}\rho \log _2 L<\infty \end{array}}} \inf _{\sigma \in {\widetilde{\mathcal {C}}}_m} F_\rho (\sigma , L) \\&{\ {\mathop {\le }\limits ^{{\text{2 }}}} \sup _{0<L\in \mathcal {T}_{\textrm{sa}}(\mathcal {H}_{m})} \inf _{\sigma \in {\widetilde{\mathcal {C}}}_m} F_\rho (\sigma , L)} \\&= \sup _{0<L\in \mathcal {T}_{\textrm{sa}}(\mathcal {H}_{m})} \inf _{\sigma \in {\widetilde{\mathcal {C}}}_m} \left\{ {{\,\textrm{Tr}\,}}[\rho \log _2 L] + \log _2(e) \left( 1 - {{\,\textrm{Tr}\,}}[\sigma L ]\right) \right\} \\&= \sup _{0<L\in \mathcal {T}_{\textrm{sa}}(\mathcal {H}_{m})} \left\{ {{\,\textrm{Tr}\,}}[\rho \log _2 L] + \log _2(e) \left( 1 - \sup _{\sigma \in {\widetilde{\mathcal {C}}}_m} {{\,\textrm{Tr}\,}}[\sigma L]\right) \right\} \\&{\mathop {=}\limits ^{{\text{3 }}}} \sup _{0<L\in \mathcal {T}_{\textrm{sa}}(\mathcal {H}_{m})} \left\{ {{\,\textrm{Tr}\,}}[\rho \log _2 L] + \log _2(e) \left( 1 - \max \left\{ \sup _{\alpha \in \mathbb {C}^m} \langle \alpha | L|\alpha \rangle ,\, 0\right\} \right) \right\} \\&{\mathop {=}\limits ^{{\text{4 }}}} \sup _{0<L\in \mathcal {T}_{\textrm{sa}}(\mathcal {H}_{m})} \left\{ {{\,\textrm{Tr}\,}}[\rho \log _2 L] + \log _2(e) \left( 1 - \sup _{\alpha \in \mathbb {C}^m} \langle \alpha | L|\alpha \rangle \right) \right\} \\&{\mathop {=}\limits ^{{\text{5 }}}} \sup _{0<L\in \mathcal {T}_{\textrm{sa}}(\mathcal {H}_{m})} \left\{ {{\,\textrm{Tr}\,}}[\rho \log _2 L] - \log _2 \sup _{\alpha \in \mathbb {C}^m} \langle \alpha |L|\alpha \rangle \right\} \\&{\mathop {\le }\limits ^{{\text{6 }}}} \sup _{0<L\in \mathcal {B}_{\textrm{sa}}(\mathcal {H}_{m})} \left\{ {{\,\textrm{Tr}\,}}[\rho \log _2 L] - \log _2 \sup _{\alpha \in \mathbb {C}^m} \langle \alpha |L|\alpha \rangle \right\} \\&{\mathop {=}\limits ^{{\text{7 }}}} \Gamma (\rho )\, . \end{aligned}$$

Here, 1 is Sion’s theorem [106], in 2 we simply extended the supremum, 3 comes from the fact that the extreme points of ${\widetilde{\mathcal {C}}}_m$ are either coherent states or 0, as it follows from (52), 4 holds because $L>0$, 5 is proved by scale invariance of the expression on the sixth line exactly as in step 1 of the proof of Lemma 20, in 6 we extended the supremum to all $0<L\in \mathcal {B}_{\textrm{sa}}(\mathcal {H}_{m})$, and finally 7 holds thanks to Lemma 34. Since Proposition 35 establishes that $\Gamma \le N^M_r\!$ on all states, we have actually proved that

63 $$\begin{aligned} N^M_r\!(\rho ) = \sup _{0<L\in \mathcal {T}_{\textrm{sa}}(\mathcal {H}_{m})} \left\{ {{\,\textrm{Tr}\,}}[\rho \log _2 L] - \log _2 \sup _{\alpha \in \mathbb {C}^m} \langle \alpha | L|\alpha \rangle \right\} = \Gamma (\rho )\, . \end{aligned}$$

The fact that *L* can be taken to be a state follows by scale invariance. $\square$

#### Proof of Corollary 22

Thanks to Theorem 19, the function $N^M_r\!$ inherits all properties of $\Gamma$, as established in Proposition 35 and Corollary 36, on the whole set of finite-entropy states. Given such a state $\rho$, the same Corollary 36 also shows that $N^{M,\infty }_r\!(\rho )\ge N^M_r\!(\rho )$. On the other hand, regularizing the inequality $N^M_r\!(\rho ) \le N_r(\rho )$ (Lemma 28) we see that $N^{M,\infty }_r\!(\rho )\le N_r^\infty (\rho )$. Remembering that $N_r^\infty (\rho ) \le N_r(\rho )$ by Corollary 29 concludes the proof of (31). Faithfulness of $N^{M,\infty }_r\!$ and hence of $N_r^\infty$ on finite-entropy states follows from the fact that $N^M_r\!$ itself is faithful (Lemma 28). $\square$

We conclude this section with the proof of Theorem 23.

#### Proof of Theorem 23

To establish (32), we apply Theorem 15 to the lower semicontinuous, weakly additive, and strongly superadditive nonclassicality monotone $\Gamma ^\infty$ (Corollary 36):

$$\begin{aligned} R(\rho \rightarrow \sigma )\le {\widetilde{R}}(\rho \rightarrow \sigma ) \le \frac{\Gamma ^\infty (\rho )}{\Gamma ^\infty (\sigma )} = \frac{N^{M,\infty }_r\!(\rho )}{N^{M,\infty }_r\!(\sigma )}\, , \end{aligned}$$

where the last equality is just (63), which is applicable because $S(\rho ), S(\sigma )<\infty$. Finally, the last estimate in (32) is a simple application of (31). $\square$

## Further Properties of Our Nonclassicality Monotones

We now present some additional results which can be useful in actual applications of Theorem 15. In particular, we present two different and independent bounds on $N_r$ and $N^M_r\!$, and a technique for approximating them in the case of infinite rank states, where analytical methods or even numerical simulations might not be enough.

### Bounds on nonclassicality monotones

We start however with a little — hopefully instructive — detour. In light of Proposition 30, one may wonder whether $N_r$ and $N^M_r\!$ can take the value $+\infty$ at all. We now set out to show that this may indeed be the case. Clearly, Proposition 30 implies that any state with this property must have infinite mean photon number.

#### Proposition 39

There exists a single-mode (infinite-energy) state $\rho \in \mathcal {D}(\mathcal {H}_{1})$ such that $N^M_r\!(\rho )=N_r(\rho )=+\infty$, i.e., $D(\rho \Vert \sigma )= D^M\!(\rho \Vert \sigma ) = +\infty$ for all classical states $\sigma \in \mathcal {C}_m$ — including those of infinite energy!

#### Proof

Let

64 $$\begin{aligned} \rho :=\frac{6}{\pi ^2} \sum _n \frac{1}{(n+1)^2}\, \vert 2^n\rangle \!\langle 2^n\vert \, \end{aligned}$$

be a modified “Basel-type state”, where the $\vert 2^n\rangle$ are Fock states. It is easy to see that $\rho$ has finite entropy. Then, because of Theorem 19 and Lemma 28, we see that

$$\begin{aligned} N_r(\rho ) \ge N^M_r\!(\rho ) {=} \Gamma (\rho ) = \sup _{h\in \mathcal {B}_{\textrm{sa}}(\mathcal {H}_{m})} \left\{ {{\,\textrm{Tr}\,}}[\rho h] - \log _2 \sup _{\alpha \in \mathbb {C}} \langle \alpha | 2^h | \alpha \rangle \right\} . \end{aligned}$$

Now, set $h_N:=\frac{1}{3} \sum _{n=0}^N n \vert 2^n\rangle \!\langle 2^n\vert$. Observe thatwhilewhere the evaluation of the limit is made possible by the fact that

$$\begin{aligned} 2^{n/3} \frac{2^{n 2^n}}{e^{2^n} (2^n)!} \sim \frac{2^{n/3}}{\sqrt{2\pi }\, 2^{n/2}} = \frac{1}{\sqrt{2\pi }\, 2^{n/6}} \end{aligned}$$

by Stirling’s formula, in the sense that the ratio between the left-hand and the right-hand sides tends to 1 as $n\rightarrow \infty$. We conclude that

$$\begin{aligned} N_r(\rho ) \ge N^M_r\!(\rho ) \ge \Gamma (\rho ) \ge \lim _{N\rightarrow \infty } \left\{ {{\,\textrm{Tr}\,}}[\rho \, h_N] - \log _2 \sup _{\alpha \in \mathbb {C}} \langle \alpha | 2^{h_N} | \alpha \rangle \right\} = +\infty \, , \end{aligned}$$

as claimed. Clearly, this construction is easily generalized to the multi-mode case, where it leads to the same conclusion. $\square$

### Estimates based on the Wehrl entropy

We now go back to the problem of estimating our nonclassicality monotones $N^M_r\!$ and $N^{M,\infty }_r\!$, already tackled in Proposition 30. The next result gives another independent upper bound for $N^M_r\!$ and $N^{M,\infty }_r\!$ in terms of the Wehrl entropy (8).

#### Proposition 40

For any finite-entropy *m*-mode state $\rho$, it holds that

65 $$\begin{aligned} -\log _2\left\| Q_\rho \right\| _\infty - S(\rho ) - m \log _2\pi \le N^M_r\!(\rho ) \le N^{M,\infty }_r\!(\rho ) \le S_W(\rho ) - S(\rho )\, , \end{aligned}$$

where $\Vert Q_\rho \Vert _\infty :=\sup _{\alpha \in \mathbb {C}^m} |Q_\rho (\alpha )|$ is the sup norm of $Q_\rho$. If instead $S(\rho )=+\infty$, then $N_r^M(\rho )=N_r^{M,\infty }(\rho )=+\infty$ as well.

#### Proof

Let us start by proving that $N^M_r\!(\rho ) \le S_W(\rho ) - S(\rho )$ whenever $S(\rho )<\infty$. We are in the situation of Theorem 19, so that we can write

66 $$\begin{aligned}{} & {} N^M_r\!(\rho ) {\mathop {=}\limits ^{{\text{1 }}}} \sup _{\omega \in \mathcal {D}(\mathcal {H}_{m})}\left\{ {{\,\textrm{Tr}\,}}[\rho \log _2\omega ] - \log _2 \sup _{\alpha \in \mathbb {C}^m} \langle \alpha |\omega |\alpha \rangle \right\} \nonumber \\{} & {} \qquad \qquad = \sup _{\omega \in \mathcal {D}(\mathcal {H}_{m})}\left\{ - S(\rho ) - D(\rho \Vert \omega ) - \log _2 \sup _{\alpha \in \mathbb {C}^m} \langle \alpha |\omega |\alpha \rangle \right\} \nonumber \\{} & {} \qquad \qquad = \sup _{\omega \in \mathcal {D}(\mathcal {H}_{m})}\left\{ - S(\rho ) - D(\rho \Vert \omega ) - \log _2 \left( \pi ^m \Vert Q_\omega \Vert _\infty \right) \right\} \nonumber \\{} & {} \qquad \qquad {\mathop {\le }\limits ^{{\text{2 }}}} \sup _{\omega \in \mathcal {D}(\mathcal {H}_{m})}\left\{ - S(\rho ) - D_{K\! L}\!\left( Q_\rho \Vert Q_\omega \right) - \log _2 \left( \pi ^m \Vert Q_\omega \Vert _\infty \right) \right\} \nonumber \\{} & {} \qquad \qquad = \sup _{\omega \in \mathcal {D}(\mathcal {H}_{m})}\left\{ - S(\rho ) + S_W(\rho ) + \int d^{2m}\alpha \, Q_\rho (\alpha ) \log _2 Q_\omega (\alpha ) - \log _2 \Vert Q_\omega \Vert _\infty \right\} \nonumber \\{} & {} \qquad \qquad {\mathop {\le }\limits ^{{\text{3 }}}} S_W(\rho ) - S(\rho )\, . \end{aligned}$$

Here, 1 is just Theorem 19, in 2 we applied the data processing inequality [122–125] (see also [45, Proposition 5.23(iv)]) to the quantum-to-classical channel $\rho \mapsto Q_\rho$, which physically corresponds to a heterodyne detection [126, 5.4.2] (for an independent proof, see Lemma B59), and finally in 3 we noted that $Q_\omega (\alpha )\le \Vert Q_\omega \Vert _\infty$ and remembered that $Q_\rho$ is a probability density function.

Since $N^M_r\!(\omega )\le S_W(\omega ) - S(\omega )$ whenever $\omega$ has finite entropy, setting $\omega =\rho ^{\otimes n}$ yields

$$\begin{aligned} N^{M,\infty }_r\!(\rho ) = \lim _{n\rightarrow \infty } \frac{1}{n} N^M_r\!(\rho ^{\otimes n}) \le \lim _{n\rightarrow \infty } \frac{1}{n} \left( S_W(\rho ^{\otimes n}) - S(\rho ^{\otimes n})\right) = S_W(\rho ) - S(\rho )\, , \end{aligned}$$

where in the last step we used the additivity of both the von Neumann entropy and the Wehrl entropy.

To prove the lower bound on $N^M_r\!$, we use Proposition 35 together with the expression (49) for $\Gamma$. Start by denoting with $\Pi$ the orthogonal projector onto the kernel of $\rho$. Then for all $\epsilon >0$ we have that $\rho +\epsilon \Pi >0$ and moreover ${{\,\textrm{Tr}\,}}\left[ \rho \log _2 (\rho +\epsilon \Pi ) \right] = {{\,\textrm{Tr}\,}}\left[ \rho \log _2 \rho \right]$, and hence

$$\begin{aligned} N^M_r\!(\rho )&\ge \sup _{0<L\in \mathcal {B}_{\textrm{sa}}(\mathcal {H}_{m})} \left\{ {{\,\textrm{Tr}\,}}\left[ \rho \log _2 L\right] - \log _2 \sup _{\alpha \in \mathbb {C}^m} \langle \alpha |L|\alpha \rangle \right\} \\&\ge \limsup _{\epsilon \rightarrow 0^+} \left\{ {{\,\textrm{Tr}\,}}\left[ \rho \log _2 (\rho +\epsilon {\Pi }) \right] - \log _2 \sup _{\alpha \in \mathbb {C}^m} \langle \alpha |(\rho +\epsilon \Pi )|\alpha \rangle \right\} \\&\ge \limsup _{\epsilon \rightarrow 0^+} \left\{ {{\,\textrm{Tr}\,}}\left[ \rho \log _2 \rho \right] - \log _2 \left( \sup _{\alpha \in \mathbb {C}^m} \langle \alpha |\rho |\alpha \rangle + \epsilon \right) \right\} \\&= {{\,\textrm{Tr}\,}}\left[ \rho \log _2 \rho \right] - \log _2 \sup _{\alpha \in \mathbb {C}^m} \langle \alpha |\rho |\alpha \rangle \\&= - S(\rho ) - m \log _2\pi - \log _2\Vert Q_\rho \Vert _\infty \end{aligned}$$

Since it relies only on Proposition 35, this lower bound holds even if $S(\rho )=+\infty$, in which case it implies that $N_r^M(\rho )=N_r^{M,\infty }(\rho )=+\infty$. This completes the proof. $\square$

We can immediately draw some interesting consequences concerning Gaussian states. Following the conventions of the excellent monograph by Serafini [126], for an *m*-mode state $\rho$ we set $s_j:={{\,\textrm{Tr}\,}}[\rho R_j]$, with $j=1,\ldots , 2m$ and $R:=(x_1,p_1\ldots , x_m,p_m)^\intercal$, and define the quantum covariance matrix by $V_{jk}:={{\,\textrm{Tr}\,}}\left[ \rho \left\{ R_j,R_k\right\} \right] - 2s_j s_k$. Gaussian states are those whose characteristic function (6) is a multivariate Gaussian, and are uniquely characterized by the vector *s* and the quantum covariance matrix *V*.

#### Corollary 41

Let $\rho$ be an arbitrary *m*-mode Gaussian state with quantum covariance matrix *V*. Then

$$\begin{aligned} \frac{1}{2} \log _2\det (V+\mathbb {1}) - S(\rho ) - m\le & {} N^M_r\!(\rho ) \le N^{M,\infty }_r\!(\rho ) \\{} & {} \le \frac{1}{2} \log _2 \det (V+\mathbb {1}) - S(\rho ) + m \log _2 (e)\, . \end{aligned}$$

#### Proof

One just needs to remember that the Husimi function $Q_\rho$ of a Gaussian state $\rho$ with quantum covariance matrix *V* is a Gaussian with (classical) covariance matrix $(V+\mathbb {1})/2$ (to see this, just set $\sigma =V$ and $\sigma _m=\mathbb {1}$ in [126, Eq. (5.139)]). This implies immediately that $\Vert Q_\rho \Vert _\infty = \pi ^{-m} \left( \det \left( \frac{V+\mathbb {1}}{2}\right) \right) ^{-1/2}$, and that the Wehrl entropy of $\rho$ satisfies

67 $$\begin{aligned} S_W(\rho ) = - \int d^{2m}\alpha \, Q_\rho (\alpha ) \log _2 \left( \pi ^m Q_\rho (\alpha )\right) = \frac{1}{2} \log _2 \det (V+\mathbb {1}) + m \log _2 (e)\, . \end{aligned}$$

This concludes the proof. $\square$

### Symmetries

A notion that we will often exploit is that of symmetry. Its implications for the variational program in Theorem 19 are as follows.

#### Proposition 42

Let $\Lambda :\mathcal {T}_{\textrm{sa}}(\mathcal {H}_{m})\rightarrow \mathcal {T}_{\textrm{sa}}(\mathcal {H}_{m})$ be a classical operation on an *m*-mode system, and let $\rho \in \mathcal {D}(\mathcal {H}_{m})$ be an invariant state, in formula $\Lambda (\rho )=\rho$. Then we have that

68 $$\begin{aligned} N^M_r\!(\rho ) = \inf _{\sigma \in \Lambda (\mathcal {C}_m)}D^M\!(\rho \Vert \sigma )\,. \end{aligned}$$

If $S(\rho )<\infty$, then it also holds that

69 $$\begin{aligned} N^M_r\!(\rho ) = \sup _{0<L \in \Lambda ^\dag (\mathcal {B}_{\textrm{sa}}(\mathcal {H}_{m}))}\left\{ {{\,\textrm{Tr}\,}}[\rho \log _2 L] - \log _2 \sup _{\alpha \in \mathbb {C}^m} \langle \alpha |L|\alpha \rangle \right\} \,. \end{aligned}$$

#### Proof

We start with (68), which follows from general and well-known arguments. We have that

$$\begin{aligned} N^M_r\!(\rho )&= \inf _{\sigma \in \mathcal {C}_m}D^M\!(\rho \Vert \sigma ) {\mathop {\ge }\limits ^{{\text{1 }}}} \inf _{\sigma \in \mathcal {C}_m} D^M\!\left( \Lambda (\rho )\Vert \Lambda (\sigma )\right) = \inf _{\sigma \in \mathcal {C}_m} D^M\!\left( \rho \Vert \Lambda (\sigma )\right) \\&=\inf _{\sigma \in \Lambda (\mathcal {C}_m)} D^M\!(\rho \Vert \sigma )\,, \end{aligned}$$

where 1 holds because of the monotonicity under channels of $D^M$. Clearly, since restricting the infimum can only increase the value of the program, it also holds that $N^M_r\!(\rho )\le \inf _{\sigma \in \Lambda (\mathcal {C}_m)} D^M\!(\rho \Vert \sigma )$. This proves (68)

To prove (69), we go back to (51). Assuming that $\Gamma ((\Lambda (\rho )) = \Gamma (\rho ) = N^M_r\!(\rho )$, as implied by Theorem 19, the derivation in (51) also shows that we can in fact restrict *L* to belong to $\Lambda ^\dag (\mathcal {B}_{\textrm{sa}}(\mathcal {H}_{m}))$. $\square$

The above result is particularly useful when the state $\rho$ under examination is invariant under a group action.

#### Corollary 43

Let $U:G\rightarrow \mathcal {B}_{\textrm{sa}}(\mathcal {H}_{m})$ be a unitary representation of a compact group *G* on the Hilbert space $\mathcal {H}_m$. Assume that *U*(*g*) maps coherent states to coherent states for all $g\in G$. Let $\rho \in \mathcal {D}(\mathcal {H}_{m})$ be a finite-entropy state such that is invariant under *G*, i.e., such that $U(g) \rho U(g)^\dag \equiv \rho$ for all $g\in G$. Then

70 $$\begin{aligned} N^M_r\!(\rho )&= \inf _{\sigma \in \mathcal {C}_m^G} D^M\!(\rho \Vert \sigma ) \end{aligned}$$

71 $$\begin{aligned}&= \sup _{0<L \in \mathcal {B}_{\textrm{sa}}^{G}(\mathcal {H}_m)} \left\{ {{\,\textrm{Tr}\,}}[\rho \log _2 L] - \log _2 \sup _{\alpha \in \mathbb {C}^m} \langle \alpha |L|\alpha \rangle \right\} , \end{aligned}$$

where a superscript *G* denotes that we restrict to *G*-invariant operators.

#### Proof

It suffices to apply Proposition 42 to the totally symmetrizing map

72 $$\begin{aligned} \Lambda _G:T\longmapsto \Lambda _G \left( T\right) :=\int _G U(g) T U^\dag (g)\, d\mu (g)\, , \end{aligned}$$

where $\mu$ denotes the left Haar measure on *G*, and the integral on the right-hand side is to be underdstood in the Bochner sense. Note that $\Lambda _G$ is a classical channel, because each *U*(*g*) maps coherent states to coherent states, and the set of classical channels is convex. $\square$

## Applications

To get a feeling of how tight the estimates in Theorem 23 for asymptotic transformation rates in the QRT of nonclassicality really are, we need to design distillation protocols that can provide lower bounds on those rates. To do so, we have to first compute or bound the resource content of the states we work with. Before going on, we fix some notation. Consider a two-mode CV quantum system with annihilation operators *a*, *b*, and pick $\lambda \in [0,1]$. The **beam splitter** with transmissivity $\lambda$ is represented by the unitary

73 $$\begin{aligned} U_\lambda :=e^{\arccos \sqrt{\lambda }\, ( a^\dag b - ab^\dag )}\, . \end{aligned}$$

Its action on operators and vectors is given by7475Therefore, thanks to (5) we see that

76 $$\begin{aligned} U_\lambda \vert \alpha \rangle \vert \beta \rangle = \vert \sqrt{\lambda }\alpha + \sqrt{1-\lambda }\beta \rangle \vert -\sqrt{1-\lambda }\alpha + \sqrt{\lambda }\beta \rangle \, . \end{aligned}$$

### Fock diagonal states

We now compute or estimate our nonclassicality monotones for some multimode Fock-diagonal states. Denoting with $\{\vert n\rangle \}_{n\in \mathbb {N}^m}$ the Fock basis, as usual, define the **totally dephasing map** $\Delta$ by

77 $$\begin{aligned} \Delta (\rho ) :=\sum _{n\in \mathbb {N}^m} \vert n\rangle \!\langle n\vert \rho \vert n\rangle \!\langle n\vert \, . \end{aligned}$$

This is a classical channel because it is of the form (72), for $G=(S^1)^{\times m}\simeq [0,2\pi )^m$ and $U(\varphi ) = e^{i \sum _j \varphi _j a_j^\dag \! a_j}$. In other words,

$$\begin{aligned} \Delta (\rho )=\frac{1}{2\pi }\int _0^{2\pi } d^m \varphi \, e^{i \sum _j \varphi _j a_j^\dag \! a_j} \rho e^{-i \sum _j \varphi _j a_j^\dag \! a_j}\, . \end{aligned}$$

Clearly, the unitary $e^{i \sum _j \varphi _j a_j^\dag \! a_j}$, which is nothing but a phase space rotation, sends coherent states to coherent states.

Applying Corollary 43 to any finite-entropy Fock-diagonal state $\rho \in \mathcal {C}_1^{\textrm{FD}}$ then yields

78 $$\begin{aligned} N^M_r\!(\rho )= & {} N^{M,\infty }_r\!(\rho ) = N_r^\infty (\rho ) = N_r(\rho ) = \inf _{\sigma \in \mathcal {C}_m^{\textrm{FD}}} D(\rho \Vert \sigma ) \nonumber \\= & {} \sup _{0<L \in \Delta (\mathcal {B}_{\textrm{sa}}(\mathcal {H}_{1}))}\left\{ {{\,\textrm{Tr}\,}}[\rho \log _2 L] - \log _2 \sup _{\alpha \in \mathbb {C}^m} \langle \alpha |L|\alpha \rangle \right\} , \end{aligned}$$

where the equalities $N^M_r\!(\rho ) = N^{M,\infty }_r\!(\rho ) = N_r^\infty (\rho ) = N_r(\rho )$ comes from the fact that the optimal state $\sigma$, being Fock-diagonal, commutes with $\rho$, and $D^M\!(\rho \Vert \sigma )=D(\rho \Vert \sigma )$ whenever $[\rho ,\sigma ]=0$; thus, $N^M_r\!(\rho ) = N_r(\rho )$, which in turn makes the whole hierarchy (31) collapse.

We now look at single-mode Fock-diagonal states with finite rank, since these will commonly be encountered in experimental applications.

#### Proposition 44

Let $\rho$ be a single-mode Fock-diagonal state with finite rank. Let $M:=\max \{n:\langle n|\rho |n\rangle \ne 0\}$. Then in (78) we can also take *L* to have the same support as that of $\rho$ (and to be positive there only). In formula,

79 $$\begin{aligned} N^M_r\!(\rho ) = \sup _{L\in {\widetilde{B}}_{\textrm{sa}}^{\textrm{FD}}(\mathcal {H}_1)} \left\{ {{\,\textrm{Tr}\,}}[\rho \log _2 L] - \log _2 \sup _{\alpha \in \left[ 0,\sqrt{M}\right] }\langle \alpha |L|\alpha \rangle \right\} \,, \end{aligned}$$

where ${\widetilde{B}}_{\textrm{sa}}^{\textrm{FD}}(\mathcal {H}_1) :=\left\{ L\in \mathcal {B}_{\textrm{sa}}(\mathcal {H}_{1}):\ L=\Delta (L),\ {{\,\textrm{supp}\,}}L = {{\,\textrm{supp}\,}}\rho ,\ P_\rho L P_\rho > 0\right\}$, and $P_\rho :\mathcal {H}_1\rightarrow {{\,\textrm{supp}\,}}\rho$ is the projector onto the finite-dimensional space ${{\,\textrm{supp}\,}}\rho$.

#### Proof

We have that

$$\begin{aligned} N^M_r\!(\rho )&= \sup _{0< L\in \Delta (\mathcal {B}_{\textrm{sa}}(\mathcal {H}_{1}))} \left\{ {{\,\textrm{Tr}\,}}[\rho \log _2 L] - \log _2 \sup _{\alpha \in \mathbb {C}} \langle \alpha |L|\alpha \rangle \right\} \\&{\mathop {=}\limits ^{{\text{1 }}}} \sup _{0< L\in \Delta (\mathcal {B}_{\textrm{sa}}(\mathcal {H}_{1}))} \left\{ {{\,\textrm{Tr}\,}}\left[ \rho \log _2 \left( P_\rho L P_\rho \right) \right] - \log _2 \sup _{\alpha \in \mathbb {C}} \langle \alpha |L|\alpha \rangle \right\} \\&{\mathop {\le }\limits ^{{\text{2 }}}} \sup _{0 < L\in \Delta (\mathcal {B}_{\textrm{sa}}(\mathcal {H}_{1}))} \left\{ {{\,\textrm{Tr}\,}}\left[ \rho \log _2 \left( P_\rho L P_\rho \right) \right] - \log _2 \sup _{\alpha \in \mathbb {C}} \langle \alpha |P_\rho L P_\rho |\alpha \rangle \right\} \\&= \sup _{L\in {\widetilde{B}}_{\textrm{sa}}^{\textrm{FD}}(\mathcal {H}_1)} \left\{ {{\,\textrm{Tr}\,}}[\rho \log _2 L] - \log _2 \sup _{\alpha \in \mathbb {C}} \langle \alpha |L|\alpha \rangle \right\} \\&{\mathop {=}\limits ^{{\text{3 }}}} \sup _{L\in {\widetilde{B}}_{\textrm{sa}}^{\textrm{FD}}(\mathcal {H}_1)} \left\{ {{\,\textrm{Tr}\,}}[\rho \log _2 L] - \log _2 \sup _{\alpha \in \left[ 0,\sqrt{M}\right] } \langle \alpha |L|\alpha \rangle \right\} . \end{aligned}$$

Here: 1 follows because $[\rho ,L]=0$ and hence ${{\,\textrm{Tr}\,}}[\rho \log _2 L]={{\,\textrm{Tr}\,}}[\rho P_\rho (\log _2 L) P_\rho ]={{\,\textrm{Tr}\,}}[\rho \log _2 (P_\rho L P_\rho )]$ (with a slight abuse of notation, we thought of $P_\rho$ as having the entire $\mathcal {H}_1$ as codomain); 2 holds thanks to the fact that $L\ge P_\rho L P_\rho$ as both *L* and $P_\rho$ are Fock-diagonal and hence commute; finally, in 3 we noticed that for $|\alpha |^2>M$ and for $L=\sum _{n=0}^M \ell _n \vert n\rangle \!\langle n\vert$ the function

$$\begin{aligned} \langle \alpha |L|\alpha \rangle = e^{-|\alpha |^2} \sum _{n=0}^M \frac{|\alpha |^{2n} \ell _n}{n!} \end{aligned}$$

becomes monotonically decreasing in $|\alpha |$, essentially because it is a sum of monotonically decreasing functions. $\square$

#### Remark 45

From Corollary B58 we know that80where $\rho _n$ is the spectral truncation of the Fock-diagonal state $\rho$. Therefore, in principle we can use Proposition 44 to approximate numerically $N^M_r\!(\rho )$ for any Fock-diagonal state $\rho$ with arbitrary precision. Explicit estimates of the error associated with each truncation can be deduced from Corollary B58.

The simplest example of Fock diagonal states is naturally given by Fock states themselves.^4^

#### Lemma 46

For a Fock state $\vert n\rangle$ we have that

81 $$\begin{aligned} N^M_r\!(\vert n\rangle \!\langle n\vert )= & {} N^{M,\infty }_r\!(\vert n\rangle \!\langle n\vert ) = N_r^\infty (\vert n\rangle \!\langle n\vert ) = N_r(\vert n\rangle \!\langle n\vert ) = \log _2 \left( \frac{n!e^n}{n^n}\right) \nonumber \\= & {} \frac{1}{2} \log _2 (2\pi n) + O(n^{-1})\, . \end{aligned}$$

#### Proof

The optimization in (79) involves a single parameter and is thus elementary. To deduce the asymptotic expansion on the righmost side, it suffices to apply Stirling’s formula. $\square$

Another example of Fock diagonal state is a noisy Fock state, e.g., a Fock state mixed with a certain amount of thermal noise. These states, herafter called **noisy Fock states**, are defined by

82 $$\begin{aligned} \rho _{n,\nu }(p):=p\vert n\rangle \!\langle n\vert +(1-p)\tau _\nu \, , \end{aligned}$$

where the thermal state $\tau _\nu$ is given in (37). In principle, we can approximate the exact value of $N^M_r\!(\rho _{n,\nu }(p))$ with arbitrary precision for any *n* and $\nu$, as pointed out in Remark 45. Let us first consider the simpler case $\nu =0$, which is a good approximation in certain regimes, e.g., optical frequencies at room temperature. The state then becomes $\rho _{n,0}(p) = p\vert n\rangle \!\langle n\vert +(1-p)\vert 0\rangle \!\langle 0\vert$, and thanks to Proposition 44 we can assume *L* to be in the form $L = \ell \vert n\rangle \!\langle n\vert + \vert 0\rangle \!\langle 0\vert$ (we already exploited the scale invariance). Now we have to perform just two nested optimizations over one real parameter each, that is,

83 $$\begin{aligned} N^M_r\!(\rho _{n,0}(p)) = \sup _{\ell >0} \left\{ p\log _2 \ell - \log _2 \max _{\alpha \in \left[ 0,\sqrt{n}\right] } e^{-\alpha ^2}\left( 1+\frac{\ell \alpha ^{2n}}{n!}\right) \right\} . \end{aligned}$$

**Fig. 2:**
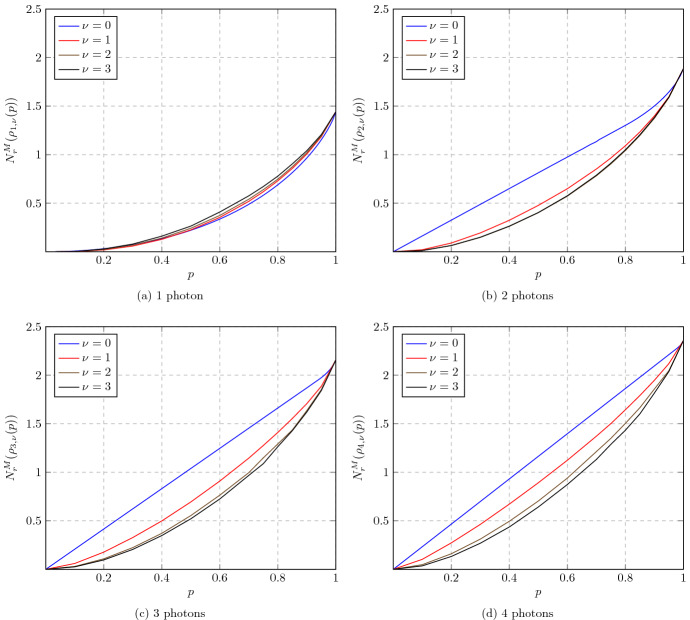
Nonclassicality for noisy Fock states: varying $\nu$ at fixed *n*

**Fig. 3:**
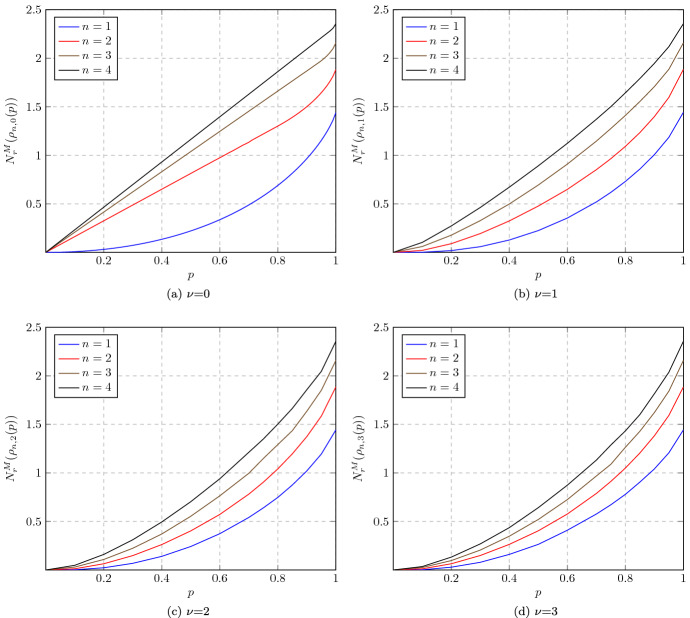
Nonclassicality for noisy Fock states: varying *n* at fixed $\nu$

For $n\le 4$ the above program can even be solved analytically, since the inner maximization reduces to solving a *n*-th order algebraic equation. For example, for $n=1$ one simply finds $\beta =\sqrt{p}$, $\ell =1/(1-p)$ and $N^M_r\!(\rho _{1,0}(p))=p+(1-p)\log _2 (1-p)$. The case of a nonzero temperature can be tackled by considering truncations of $\rho$ and performing numerical optimizations until some tolerance threshold is achieved. The results for different values of $\nu$ and *n* are reported in Figs. 2 and 3.

### Schrödinger cat states

For $\alpha \in \mathbb {C}$, the associated **Schrödinger cat states** (or simply **cat state**) is defined by [71]

84 $$\begin{aligned} \vert \psi _{\alpha }^\pm \rangle :=\frac{1}{\sqrt{2\left( 1\pm e^{-2|\alpha |^2}\right) }} \left( \vert \alpha \rangle \pm \vert -\alpha \rangle \right) . \end{aligned}$$

**Fig. 4:**
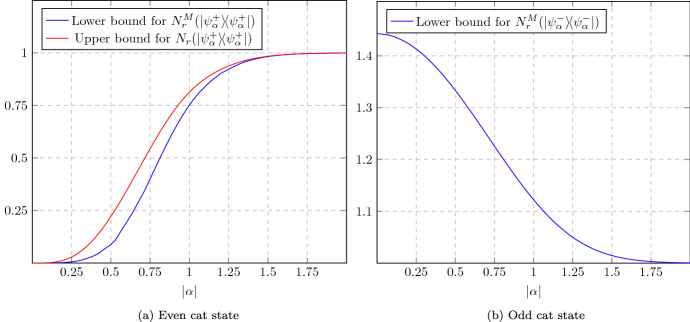
Bounds for the nonclassicality of a cat state, for different values of $|\alpha |$

It is a nonclassical state for all $\alpha \ne 0$. Since a phase space rotation acts as $e^{i\varphi a^\dag a}\vert \psi _\alpha ^\pm \rangle = \vert \psi _{e^{i\varphi }\alpha }^\pm \rangle$, and all of our nonclassicality monotones are left invariant by such transformations, in what follows we can without loss of generality assume that $\alpha \in \mathbb {R}$. Now, for a cat state with real $\alpha$, we can consider the group $G=\mathbb {Z}^2$ and its representation $U:G\rightarrow \mathcal {B}_{\textrm{sa}}(\mathcal {H}_{1})$ given by the reflection with respect to the real and/or imaginary axis. Applying Corollary 43 to this setting (with $m=1$) shows immediately that (70)–(71) hold with $\mathcal {C}_1^G$ and $\mathcal {B}_{\textrm{sa}}^{G}(\mathcal {H}_1)$ being the sets of classical states and bounded operators that are invariant under reflections with respect to the real and/or imaginary axis. A lower bound for $N^M_r\!(\psi _\alpha ^\pm )$ can be easily computed by setting a maximum rank for *L* in the second line of (71) and then optimizing numerically. When ${{\,\textrm{rk}\,}}L\le 3$, in order to preserve the symmetry, *L* must be supported on the subspace $V={{\,\textrm{span}\,}}(\vert \alpha \rangle ,\vert -\alpha \rangle ,\vert 0\rangle )$. Analogously, an upper bound for $N_r(\psi _\alpha ^\pm )$ can be found with a classical $\sigma$ belonging to *V*. In Fig. 4 we report these two bounds for the even cat state $\psi _\alpha ^+$, and an analogous lower bound for $N^M_r(\vert \psi ^-_\alpha \rangle \!\langle \psi ^-_\alpha \vert )$.

### Squeezed states

A single-mode **squeezed vacuum state** is defined by [127, Eq. (3.7.5)]

85 $$\begin{aligned} \vert \zeta _{r,\phi }\rangle =\frac{1}{\sqrt{\cosh (r)}} \sum _{n=0}^\infty \sqrt{\left( {\begin{array}{c}2n\\ n\end{array}}\right) } \left( -\frac{1}{2}\, e^{i\phi } \tanh (r)\right) ^n \vert 2n\rangle \,. \end{aligned}$$

Since changing $\phi$ amounts to a simple rotation in phase space, and this cannot modify the value of any of our nonclassicality monotones, we will assume $\phi =0$ from now on. A squeezed state $\zeta _r:=\zeta _{r,0}$ has always finite energy $E(\psi _r)=\sinh ^2(r)$, and hence we can use Proposition 30 to get the upper bound

$$\begin{aligned} N_r(\zeta _r) \le g(\sinh ^2(r)) = 2\log _2 \cosh r - 2 \sinh ^2(r) \log _2\tanh (r)\,. \end{aligned}$$

**Fig. 5:**
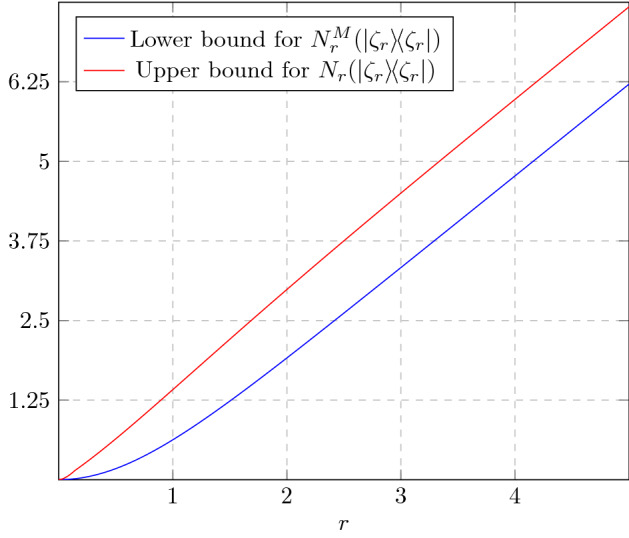
Bounds for the nonclassicality of a squeezed state, for different values of *r*

A second upper bound on $N_r$ can be found by considering a (classical) squeezed thermal state

$$\begin{aligned} \sigma _s = S(s)\tau _{N(s)}S^\dagger (s)=\sqrt{\frac{2}{\pi (e^{4s}-1)}} \int _{-\infty }^{+\infty } dt\, e^{-\frac{2t^2}{e^{4s}-1}}\, \vert it\rangle \!\langle it\vert \, ,\quad s\ge 0\, , \end{aligned}$$

where *S*(*s*) is the usual squeezing unitary with real parameter *s*, and plugging it in the infimum that defines $N_r$ (cf. (21)), i.e.,

$$\begin{aligned} N_r(\zeta _r)\le&\ \inf _{s\ge 0} D(\zeta _r\Vert \sigma _s) = \inf _{s\ge 0} \left( \log _2 (1+N(s)) + 2\sinh ^2(r-s) \log _2 \left( 1+\frac{1}{N(s)}\right) \right) , \end{aligned}$$

where $N(s) :=\frac{e^{2s}-1}{2}$. The rightmost side of the above expression can be easily optimized numerically. A lower bound on $N^M_r\!$ can be found from Corollary 41. All these estimates are plotted in Fig. 5.

### Fock state dilution

Now we are ready to report an example in which the bound in Theorem 23 is (asymptotically) tight.

#### Proposition 47

Let $0<p\le 1$ and $n\ge 2$ be fixed. Consider the transformation $\rho _{n,0}(p)\rightarrow \vert n\!-\!1\rangle \!\langle n\!-\!1\vert$, where the noisy Fock state is defined in (82). It holds that86with the upper bound being given by Theorem 23.

#### Proof

We start with the lower bound. Consider the following protocol, implemented with only linear optics, destructive measurements, and feed forward.

1. We send $\rho _{n,0}(p)$ into a beam splitter with transmissivity $\lambda$ whose second mode’s initial state is the vacuum.
2. We perform photon counting on the ancillary mode.
3. If we measure 0 photons, the output state of the remaining mode is $\rho _{n,0}(p')$, with $p':=\frac{p\lambda ^n}{p\lambda ^n + 1 - p}$. We restart with step (1).
4. If we measure 1 photon, the output state of the remaining mode is $\vert n-1\rangle$, and we have succeeded.
5. If we measure 2 or more photons, the protocol is aborted.

Using the well-known formula [128]

87 $$\begin{aligned} U_\lambda \vert n,0\rangle = \lambda ^{\frac{n}{2}} \sum _{\ell =0}^{n} (-1)^\ell \sqrt{\left( {\begin{array}{c}n\\ \ell \end{array}}\right) } \left( \frac{1-\lambda }{\lambda }\right) ^{\frac{\ell }{2}} \vert n-\ell , \ell \rangle \, , \end{aligned}$$

a lengthy but straightforward calculation shows that the global probability of success of this protocol is

88 $$\begin{aligned} P_{s}(n,p;\lambda ) = \frac{pn (1-\lambda )\lambda ^{2n-1}}{(1-\lambda ^n)(p\lambda ^n + 1-p)}\, . \end{aligned}$$

Since we can take $\lambda$ arbitrarily close to 1, we see that

$$\begin{aligned} R\left( \rho _{n,0}(p)\rightarrow \vert n\!-\!1\rangle \!\langle n\!-\!1\vert \right) \ge \lim _{\lambda \rightarrow 1^-} P_{s}(n,p;\lambda ) = p\, , \end{aligned}$$

which proves the lower bound.

As for the upper bound, using (78) together with convexity and (81), we see that

$$\begin{aligned} N^{M,\infty }_r\!(\rho _{n,0}(p)) = N^{M,\infty }_r\!(\rho _{n,0}(p)) \le p N^M_r\!(\vert n\rangle \!\langle n\vert ) = p \log _2\left( \frac{n!\, e^n}{n^n} \right) . \end{aligned}$$

Leveraging once again (81), this entails thatThis completes the proof. $\square$

### Cat state manipulation

Let us now discuss some protocols to transform cat states, enlarging or reducing their amplitude $\alpha$. Hereafter we take without loss of generality $\alpha$ to be real. The first transformation we consider is amplification: $\psi _\alpha ^+\rightarrow \psi _{\sqrt{2} \alpha }^+$. Lund et al. [107] have provided a protocol that achieves exact conversion of two copies of the initial state with probability

89 $$\begin{aligned} P_{\textrm{Lund}}\left( \psi _\alpha ^+ \otimes \psi _\alpha ^+ \rightarrow \psi _{\sqrt{2} \alpha }^+\right) = \frac{e^{-\alpha ^2} \cosh (2\alpha ^2) \sinh ^2\left( \alpha ^2/2\right) }{\cosh ^2(\alpha ^2)}\, . \end{aligned}$$

Hence,

90 $$\begin{aligned} R\left( \psi _\alpha ^+ \rightarrow \psi _{\sqrt{2} \alpha }^+\right)\ge & {} \frac{1}{2} P_{\textrm{Lund}}\left( \psi _\alpha ^+ \otimes \psi _\alpha ^+ \rightarrow \psi _{\sqrt{2} \alpha }^+\right) \nonumber \\= & {} \frac{e^{-\alpha ^2} \cosh (2\alpha ^2) \sinh ^2\left( \alpha ^2/2\right) }{2 \cosh ^2(\alpha ^2)}\, . \end{aligned}$$

Mimicking the protocol of Lund et al. but employing slightly better (yet less realistic) measurements, we are able to obtain a better bound.

#### Proposition 48

In the QRT of nonclassicality it is possible to achieve exact conversion $\psi _\alpha ^+ \otimes \psi _\alpha ^+ \rightarrow \psi _{\sqrt{2} \alpha }^+$ with probability

91 $$\begin{aligned} P_{\textrm{our}}\left( \psi _\alpha ^+ \otimes \psi _\alpha ^+ \rightarrow \psi _{\sqrt{2} \alpha }^+\right) = \frac{1}{2} \tanh ^2(\alpha ^2)\, . \end{aligned}$$

Therefore,

92 $$\begin{aligned} R\left( \psi _\alpha ^+ \rightarrow \psi _{\sqrt{2} \alpha }^+\right) \ge \frac{1}{4}\tanh ^2(\alpha ^2)\, . \end{aligned}$$

#### Proof

Consider the following protocol. Apply a beam splitter with trasmissivity 1/2 to the initial state $\vert \psi ^+_\alpha \rangle \vert \psi ^+_\alpha \rangle$. Using (76), we obtain that

$$\begin{aligned} U_{1/2} \vert \psi ^+_\alpha \rangle \vert \psi ^+_\alpha \rangle&= \frac{1}{2\left( 1+ e^{-2\alpha ^2}\right) } \left( \vert 0\rangle \vert \sqrt{2} \alpha \rangle + \vert 0\rangle \vert - \sqrt{2} \alpha \rangle + \vert \sqrt{2} \alpha \rangle \vert 0\rangle + \vert -\sqrt{2} \alpha \rangle \vert 0\rangle \right) \\&= \frac{\sqrt{\cosh (2\alpha ^2)}}{2 \cosh (\alpha ^2)} \left( \vert 0\rangle \vert \psi _{\sqrt{2} \alpha }^+\rangle + \vert \psi _{\sqrt{2} \alpha }^+\rangle \vert 0\rangle \right) . \end{aligned}$$

Carrying out on the second mode the measurement $\{\vert \chi \rangle \!\langle \chi \vert , \mathbb {1}-\vert \chi \rangle \!\langle \chi \vert \}$, with

$$\begin{aligned} \vert \chi \rangle :=\frac{1}{\sqrt{2} \sinh (\alpha ^2)}\left( \sqrt{\cosh (2\alpha ^2)} \vert 0\rangle - \vert \psi _{\sqrt{2} \alpha }^+\rangle \right) , \end{aligned}$$

yields

$$\begin{aligned} {}_2{\langle \chi |U_{1/2} |\psi _\alpha ^+ \psi _\alpha ^+\rangle }{_{12}} = \frac{1}{\sqrt{2}}\tanh (\alpha ^2)\vert \psi _{\sqrt{2} \alpha }^+\rangle \, , \end{aligned}$$

where the subscripts identify different modes. Computing the norm of the above vector yields (91) and this in turn (92). $\square$

We now move on to cat state dilution. We consider the slightly simpler task of balanced dilution $\psi _{\sqrt{2} \alpha }^+ \rightarrow \psi ^+_\alpha \otimes \psi _\alpha ^-$.

#### Proposition 49

In the QRT of nonclassicality it holds that

93 $$\begin{aligned} R\left( \psi _{\sqrt{2} \alpha }^+ \rightarrow \psi ^+_\alpha \otimes \psi _\alpha ^- \right) \ge \frac{\sinh ^2(\alpha ^2)}{2 \cosh (2\alpha ^2)}\, . \end{aligned}$$

#### Proof

Consider the following protocol. Apply a beam splitter with trasmissivity 1/2 to the initial state $\vert \psi ^+_{\sqrt{2} \alpha }\rangle \vert 0\rangle$. Using one again (76), we obtain that

$$\begin{aligned} U_{1/2} \vert \psi ^+_{\sqrt{2}\alpha }\rangle \vert 0\rangle&= \frac{1}{\sqrt{2\left( 1 + e^{-4\alpha ^2} \right) }} \left( \left( 1+e^{-2\alpha ^2}\right) \vert \psi _\alpha ^+\rangle \vert \psi _\alpha ^+\rangle + \left( 1-e^{-2\alpha ^2}\right) \vert \psi _\alpha ^-\rangle \vert \psi _\alpha ^-\rangle \right) . \end{aligned}$$

Therefore, measuring the second mode in the orthonormal basis whose first two elements are $\vert \psi _\alpha ^+\rangle$ and $\vert \psi _\alpha ^-\rangle$, we obtain that

$$\begin{aligned} {}_{2}{\langle \psi ^+_\alpha | U_{1/2} |\psi ^+_{\sqrt{2}\alpha }, 0\rangle }{_{12}}&= \frac{\cosh (\alpha ^2)}{\sqrt{\cosh (2\alpha ^2)}}\, \vert \psi ^\pm _\alpha \rangle \, , \\ {}_{2}{\langle \psi ^-_\alpha | U_{1/2} |\psi ^+_{\sqrt{2}\alpha }, 0\rangle }{_{12}}&= \frac{\sinh (\alpha ^2)}{\sqrt{\cosh (2\alpha ^2)}}\, \vert \psi ^\mp _\alpha \rangle \, . \end{aligned}$$

Computing the norms of the vectors on the right-hand side yields the estimates

$$\begin{aligned} P_{\textrm{our}} \left( \psi _{\sqrt{2} \alpha }^+ \rightarrow \psi _{\alpha }^+\right)&= \frac{\cosh ^2(\alpha ^2)}{\cosh (2\alpha ^2)}\, , \\ P_{\textrm{our}} \left( \psi _{\sqrt{2} \alpha }^+ \rightarrow \psi _{\alpha }^-\right)&= \frac{\sinh ^2(\alpha ^2)}{\cosh (2\alpha ^2)}\, . \end{aligned}$$

Applying the above protocol to *n* copies of $\psi _{\sqrt{2} \alpha }^+$ yields, in the limit of large *n*, at least $\frac{n \sinh ^2(\alpha ^2)}{2\cosh (2\alpha ^2)}$ copies of $\psi _{\alpha }^+\otimes \psi _{\alpha }^-$. Hence,

$$\begin{aligned} R\left( \psi _{\sqrt{2} \alpha }^+ \rightarrow \psi ^+_\alpha \otimes \psi _\alpha ^- \right) \ge \frac{\sinh ^2(\alpha ^2)}{2 \cosh (2\alpha ^2)}\, , \end{aligned}$$

which completes the proof. $\square$

Finally, we upper bound the maximal asymptotic transformation rates of both amplification and dilution of cat states by means of the formula 32, and the numerical results reported in Fig. 1.

## Data Availability

No data sets were generated during this study.

## Appendix A: Restricted Asymptotic Continuity in Infinite Dimensions

Among the many properties that a monotone may have, one of the most desirable is, in many scenarios, some form of continuity, since it allows to relate the resource content of a state to that of a “good” approximation of it. The most widely used notion of continuity, when it comes to resource monotones in finite-dimensional QRT, is that of asymptotic continuity [13] (Definition 6(c)). Its popularity is due to its role in proving bounds on asymptotic rates. Of course, the notion of asymptotic continuity becomes empty in infinite dimensions, ultimately leading to the “asymptotic continuity catastrophe” discussed in the Introduction. In this Appendix, we show how asymptotic continuity can be defined in a certain sense for infinite-dimensional systems as well, why this extension presents some substantial limitations, and in which sense Theorem 15 overcomes such limitations.

### Abstract approach

The traditional approach to the problem of bounding asymptotic transformation rates in infinite-dimensional QRTs makes use of the notion of restricted asymptotic continuity [16–19, 35]. Let us give a formal definition, taken from [35, Corollary 7].

#### Definition A50

Let $({\mathscr {S}},{\mathscr {F}})$ be a QRT equipped with a monotone *G*. For some $B\in {\mathscr {S}}$, fix a family of states ${\mathscr {T}} = \left\{ {\mathscr {T}}_{B^n}\right\} _{n\in \mathbb {N}_+}$, with ${\mathscr {T}}_{B^n}\subseteq \mathcal {D}(\mathcal {H}_{B^n}) = {\mathscr {D}}\left( \mathcal {H}^{\otimes n}_B \right)$. We say that *G* is **weakly asymptotically continuous on** $\varvec{{\mathscr {T}}}$ if for all sequence of states $(\rho _n)_{n\in \mathbb {N}_+}$ and $(\sigma _n)_{n\in \mathbb {N}_+}$ with $\rho _n,\sigma _n\in {\mathscr {T}}_{B^n}$ and $\lim _{n\rightarrow \infty } \left\| \rho _n-\sigma _n\right\| _1 = 0$ it holds that

A1 $$\begin{aligned} \lim _{n\rightarrow \infty } \frac{\left| G(\rho _n) - G(\sigma _n) \right| }{n} = 0\, . \end{aligned}$$

Formally, from the above definition the following can be deduced.

#### Proposition A51

Let $({\mathscr {S}},{\mathscr {F}})$ be a QRT equipped with a weakly additive monotone *G*. Consider $A,B\in {\mathscr {S}}$, and assume that there exists a family of states ${\mathscr {T}} = \left\{ {\mathscr {T}}_{B^n}\right\} _{n\in \mathbb {N}_+}$ on which *G* is weakly asymptotically continuous. Pick $\rho _A\in \mathcal {D}(\mathcal {H}_{A})$ and $\sigma _B\in \mathcal {D}(\mathcal {H}_{B})$ with $\sigma _B^{\otimes n}\in {\mathscr {T}}_{B^n}$ for all sufficiently large *n*, and consider the modified asymptotic transformation rate

A2 $$\begin{aligned}{} & {} R_{{\mathscr {T}}}(\rho _{A} \rightarrow \sigma _{B}) :=\sup \left\{ r:\ \lim _{n\rightarrow \infty } \inf _{\begin{array}{c} \Lambda _n\in {\mathscr {F}}\left( A^n\rightarrow B^{\left\lfloor rn\right\rfloor }\right) \\ \Lambda _n(\rho _A^{\otimes n})\in {\mathscr {T}}_{B^n} \end{array}} \left\| \Lambda _n\left( \rho _{A}^{\otimes n}\right) - \sigma _{B}^{\otimes \left\lfloor r n\right\rfloor } \right\| _1 = 0 \right\} . \nonumber \\ \end{aligned}$$

Then it satisfies:

A3 $$\begin{aligned} R_{{\mathscr {T}}}(\rho _{A} \rightarrow \sigma _{B}) \le \frac{G(\rho _A)}{G(\sigma _B)}\, , \end{aligned}$$

whenever the right-hand side is well defined.^5^

#### Proof

For any sequence of free operations $\Lambda _n\in {\mathscr {F}}\left( A^n \rightarrow B^{\left\lfloor rn\right\rfloor }\right) , \Lambda _n(\rho _A^{\otimes n})\in {\mathscr {T}}_{B^n}$ satisfying

$$\begin{aligned} \liminf _{n\rightarrow \infty } \left\| \Lambda _n\!\left( \rho _{\!A}^{\otimes n}\right) - \sigma _{\!B}^{\otimes \left\lfloor r n\right\rfloor } \right\| _1\!\! = 0\,, \end{aligned}$$

it holds that

$$\begin{aligned} G(\rho _A)&{\mathop {=}\limits ^{{\text{1 }}}} \liminf _{n\rightarrow \infty } \frac{1}{n}\, G\left( \rho _A^{\otimes n} \right) \\&{\mathop {\ge }\limits ^{{\text{2 }}}} \liminf _{n\rightarrow \infty } \frac{1}{n}\, G\left( \Lambda _n\left( \rho _A^{\otimes n}\right) \right) \\&\ge \liminf _{n\rightarrow \infty } \left( \frac{1}{n}\, G\left( \sigma _B^{\otimes \left\lfloor rn\right\rfloor } \right) - \frac{1}{n} \left| G\left( \Lambda _n\left( \rho _A^{\otimes n}\right) \right) - G\left( \sigma _B^{\otimes \left\lfloor rn\right\rfloor } \right) \right| \right) \\&{\mathop {=}\limits ^{{\text{3 }}}} \liminf _{n\rightarrow \infty } \frac{\left\lfloor rn\right\rfloor }{n}\, G\left( \sigma _B \right) \\&= r\, G \left( \sigma _B\right) . \end{aligned}$$

Here, 1 follows from weak additivity, 2 from monotonicity, and 3 from weak asymptotic continuity and again weak additivity. $\square$

The main problem with Proposition A51 is that the rate in (A2) only takes into account a restricted set of possible free transformations. We will see what this means in a physically relevant setting below.

### A physically interesting case: energy-constrained asymptotic continuity

The above definition may seem rather abstract. However, there is a physically very natural scenario where it can be applied. Let us assume that a certain system $B\in {\mathscr {S}}$ is equipped with a Hamiltonian (i.e., a self-adjoint operator) $H_B$. Let us assume that the Hamiltonians add up without interaction terms upon taking multiple copies of *B*, in formula $H_{B^n} = H_B \otimes \mathbb {1}_B^{\otimes (n-1)} + \mathbb {1}_B \otimes H_B \otimes \mathbb {1}_B^{\otimes (n-2)} + \ldots + \mathbb {1}_B^{\otimes (n-1)} \otimes H_B$. Now, for a real number *E*, set

A4 $$\begin{aligned} {\mathscr {T}}_{B^n}^E :=\left\{ \rho \in \mathcal {D}(\mathcal {H}_{B^n}):\, {{\,\textrm{Tr}\,}}\left[ \rho \, H_{B^n} \right] \le nE \right\} . \end{aligned}$$

Basically, we are considering states whose energy increases at most linearly in the number of systems *n*. When the set (A4) is chosen in Definition A50, the corresponding notion of weakly asymptotic continuity becomes a physically relevant and indeed fruitful one.

#### Definition A52

Let $({\mathscr {S}},{\mathscr {F}})$ be a QRT endowed with a monotone *G*. Let $B\in {\mathscr {S}}$ be equipped with a Hamiltonian $H_B$. If for all *E* the monotone *G* is weakly asymptotically continuous on the set ${\mathscr {T}}^E$ defined by (A4), then we say that it is **asymptotically continuous in the presence of an energy constraint**, or **EC asymptotically continuous** for short.

In practice, the above definition just means that whenever $(\rho _n)_{n\in \mathbb {N}_+}$ and $(\sigma _n)_{n\in \mathbb {N}_+}$ are sequences of states with $\rho _n, \sigma _n \in \mathcal {D}(\mathcal {H}_{A^n})$, ${{\,\textrm{Tr}\,}}[\rho _n H_{A^n}], {{\,\textrm{Tr}\,}}[\sigma _n H_{A^n}]\le nE$ (for a fixed but arbitrary real number *E*), and moreover $\lim _{n\rightarrow \infty } \left\| \rho _n - \sigma _n\right\| _1=0$, then

A5 $$\begin{aligned} \lim _{n\rightarrow \infty } \frac{\left| G(\rho _n) - G(\sigma _n)\right| }{n} = 0\, . \end{aligned}$$

This definition turns out to encompass a sufficiently wide set of monotones. For example, Shirokov has proved that many important entanglement monotones are EC asymptotically continuous, with respect to several physically relevant Hamiltonians [17–19, 35]. To deduce a useful result from Proposition A51 we need to fix some terminology.

#### Definition A53

([130, p. 9]) Let *A*, *B* be two quantum system equipped with Hamiltonians $H_A,H_B$. A quantum channel $\Lambda :\mathcal {T}_{\textrm{sa}}(\mathcal {H}_{A})\rightarrow \mathcal {T}_{\textrm{sa}}(\mathcal {H}_{B})$ from *A* to *B* is called $\varvec{(\kappa ,\delta )}$**-energy-limited** if $\Lambda ^\dag (H_B) \le \kappa H_A + \delta$, with $\Lambda ^\dag :\mathcal {B}_{\textrm{sa}}(\mathcal {H}_{B})\rightarrow \mathcal {B}_{\textrm{sa}}(\mathcal {H}_{A})$ being the adjoint of $\Lambda$. The set of such channels will be denoted with $\textrm{EL}_{\kappa ,\delta }(A\rightarrow B)$, where the choice of the Hamiltonians is not made explicit and assumed to be clear from the context.

In such a setting, directly from Proposition A51 we deduce the following.

#### Proposition A54

Let $({\mathscr {S}},{\mathscr {F}})$ be a QRT. Let $H_A,H_B$ be two Hamiltonians on $A,B\in {\mathscr {S}}$, and let *G* be a weakly additive monotone that is EC asymptotically continuous. Then for all $\rho _A\in \mathcal {D}(\mathcal {H}_{A})$ and $\sigma _B\in \mathcal {D}(\mathcal {H}_{B})$ with finite energy (i.e., such that ${{\,\textrm{Tr}\,}}[\rho _A H_A], {{\,\textrm{Tr}\,}}[\sigma _B H_B]<\infty$) the uniformly energy-constrained (UEC) asymptotic transformation rate defined by

A6 $$\begin{aligned}{} & {} R_{\text {UEC}}(\rho _{A} \rightarrow \sigma _{B}) \nonumber \\{} & {} \quad :=\sup _{0<\kappa ,\delta <\infty } \sup \left\{ r:\ \lim _{n\rightarrow \infty } \inf _{ \Lambda _n\in {\mathscr {F}}\left( A^n\rightarrow B^{\left\lfloor rn\right\rfloor }\right) \, \cap \, \textrm{EL}_{\kappa ,\delta }\left( A^n\rightarrow B^{\left\lfloor rn\right\rfloor } \right) } \left\| \Lambda _n\left( \rho _{A}^{\otimes n}\right) - \sigma _{B}^{\otimes \left\lfloor r n\right\rfloor } \right\| _1 = 0 \right\} \nonumber \\ \end{aligned}$$

satisfies that

A7 $$\begin{aligned} R_{\text {UEC}}(\rho _{A} \rightarrow \sigma _{B}) \le \frac{G(\rho _A)}{G(\sigma _B)}\, , \end{aligned}$$

whenever the right-hand side is well defined.

#### Proof

Let $E:=\max \left\{ {{\,\textrm{Tr}\,}}[\rho _A H_A],\, {{\,\textrm{Tr}\,}}[\sigma _B H_B]\right\} <\infty$. Fix arbitrary $0<\kappa ,\delta <\infty$, and let the rate *r* be achievable in (A6) by means of a sequence of protocols $\Lambda _n\in {\mathscr {F}}\left( A^n\rightarrow B^{\left\lfloor rn\right\rfloor }\right) \, \cap \, \textrm{EL}_{\kappa ,\delta }\left( A^n\rightarrow B^{\left\lfloor rn\right\rfloor } \right)$. Then for sufficiently large *n* it holds that

$$\begin{aligned} {{\,\textrm{Tr}\,}}\left[ \sigma _B^{\otimes n} H_{B^n}\right]&= n {{\,\textrm{Tr}\,}}[\sigma _B H_B] \le nE \le n E' , \\ {{\,\textrm{Tr}\,}}\left[ \Lambda _n\left( \rho _A^{\otimes n}\right) H_{B^n}\right]&= {{\,\textrm{Tr}\,}}\left[ \rho _A^{\otimes n} \Lambda _n^\dag \left( H_{B^n}\right) \right] \le {{\,\textrm{Tr}\,}}\left[ \rho _A^{\otimes n} (\kappa H_{A^n} + \delta )\right] \\&= n\kappa {{\,\textrm{Tr}\,}}[\rho _A H_A] + \delta \le n\kappa E + \delta \le n E' , \end{aligned}$$

where we set $E':=\max \{\kappa ,1\} (E+1)$. Since *G* is asymptotically continuous on the set ${\mathscr {T}}^{E'}$, and we have just shown that $\sigma _B^{\otimes n},\, \Lambda _n\left( \rho _A^{\otimes n}\right) \in {\mathscr {T}}^{E'}$, then we can apply Proposition A51 and conclude the proof. $\square$

The reason why Proposition A54 is ultimately not satisfactory, and why we on the contrary deem Theorem 15 more compelling, is twofold. First, Proposition A54 only allows us to bound the standard asymptotic transformation rate (Definition 10), while we have seen that in certain settings the relevant quantity is the maximal asymptotic transformation rate (Definition 11). Secondly, the above result only takes into account sequences of protocols $(\Lambda _n)_{n}$ that are *uniformly* energy-constrained, meaning that the output energy is at most $E_{\text {out}}\le \kappa E_{\text {in}} + \delta$, with $\kappa$ and $\delta$ fixed for the whole sequence. If each $\Lambda _n$ is $(\kappa _n,\delta _n)$-energy-limited for each *n*, but $\limsup _{n\rightarrow \infty } \kappa _n = +\infty$ or $\limsup _{n\rightarrow \infty } \frac{\delta _n}{n}{=+\infty }$, the above method does not seem to tell us much about the corresponding rate, even when the initial and final states have a fixed (and finite) energy. Therefore, for instance, a sequence of free operations on CV systems where each $\Lambda _n$ involves either (a) a squeezing whose intensity increases with *n* and tends to $\infty$ in the limit $n\rightarrow \infty$; or (b) a displacement unitary whose parameter $\alpha _n$ is superlinear in *n*, are excluded from the bound in Proposition A54.

As we have seen, our Theorem 15 eliminates the need for both of these requirements, and instead provides ultimate bounds on maximal (instead of standard) asymptotic transformation rates, in a setting where the free protocols employed are otherwise totally unconstrained.

## Appendix B: Approximation by Spectral Truncation

Here we study the problem of approximating $N^M_r\!$ by truncating the input state. The forthcoming Corollary B58 has been used to check the numerical validity of the plots in Sect. 9.1 We state first a useful lemma, whose proof follows closely that of [131, Lemma 7], with some adaptations made to fit our infinite-dimensional case. In what follows, for a trace class operator $X\in \mathcal {T}_{\textrm{sa}}(\mathcal {H}_{})$ with decomposition $X = X_+ - X_-$ into positive and negative parts, we denote with $|X|:=X_+ + X_-$ its absolute value.

### Lemma B55

Let $\rho ,\sigma \in \mathcal {D}(\mathcal {H}_{m})$ be two *m*-mode states, and set $\epsilon :=\frac{1}{2} \left\| \rho -\sigma \right\| _1$. Assume that the operator $|\rho -\sigma |$ has finite mean photon number $E:={{\,\textrm{Tr}\,}}\left[ |\rho -\sigma | \left( \sum \nolimits _{j=1}^n a_j^\dag a_j\right) \right] < \infty$. Then, for $F=N^M_r\!, N_r^\infty , N_r$ it holds that

B1 $$\begin{aligned} \left| F(\rho ) - F(\sigma ) \right| \le m\, \epsilon \, g\left( \frac{E}{m\,\epsilon } \right) + g(\epsilon )\, , \end{aligned}$$

where $$g(x) = (1+x) \log _2(1+x) - x \log _2 x$$.

### Remark B56

Recently, Shirokov [17] has put forward a more general technique that allows to obtain general continuity results for relative entropy distance measures in infinite dimensions, thus removing the need to make any assumption concerning the operator $|\rho -\sigma |$. While theoretically superior, his bounds are less tight and ultimately not suited for our practical purposes.

### Proof of Lemma B55

We start with the case where $F=N_r$. Here we actually prove that

B2 $$\begin{aligned} \left| N_r(\rho \otimes \tau ) - N_r(\sigma \otimes \tau ) \right| \le m\, \epsilon \, g\left( \frac{E}{m\,\epsilon } \right) + g(\epsilon )\, , \end{aligned}$$

for all *n*-mode auxiliary states $\tau$. Call $h_2(p):=- p \log _2 p - (1-p) \log _2(1-p)$ the binary entropy function. Using the convexity of $N_r$ (Lemma 28) together with [45, Proposition 5.24], it is not difficult to observe, as done by Winter [131, Lemma 7], that

B3 $$\begin{aligned} p N_r(\rho _1){} & {} + (1-p) N_r(\rho _2) - h_2(p) \le N_r(p\rho _1 + (1-p) \rho _2) \nonumber \\{} & {} \quad \le p N_r(\rho _1) + (1-p) N_r(\rho _2)\, . \end{aligned}$$

We now construct two states $\delta , \delta '\in \mathcal {D}(\mathcal {H}_{m})$ such that

$$\begin{aligned} \rho - \sigma = \epsilon \left( \delta - \delta '\right) ,\quad |\rho - \sigma | = \epsilon \left( \delta + \delta '\right) . \end{aligned}$$

In particular, the mean photon number of $\delta$ satisfies that

$$\begin{aligned} {{\,\textrm{Tr}\,}}\left[ \delta \left( \sum \nolimits _{j=1}^n a_j^\dag a_j\right) \right] \le \frac{1}{\epsilon } {{\,\textrm{Tr}\,}}\left[ |\rho -\sigma | \left( \sum \nolimits _{j=1}^n a_j^\dag a_j\right) \right] \le \frac{E}{\epsilon }\, . \end{aligned}$$

Now, set

$$\begin{aligned} \omega :=\frac{1}{1+\epsilon }\, \rho + \frac{\epsilon }{1+\epsilon }\, \delta ' = \frac{1}{1+\epsilon }\, \sigma + \frac{\epsilon }{1+\epsilon }\, \delta \, . \end{aligned}$$

Then, on the one hand

$$\begin{aligned} N_r(\omega \otimes \tau )&{\mathop {\le }\limits ^{{\text{1 }}}} \frac{1}{1+\epsilon }\, N_r(\sigma \otimes \tau ) + \frac{\epsilon }{1+\epsilon }\, N_r(\delta \otimes \tau ) \\&{\mathop {\le }\limits ^{{\text{2 }}}} \frac{1}{1+\epsilon }\, N_r(\sigma \otimes \tau ) + \frac{\epsilon }{1+\epsilon } \left( m\, g\left( \frac{E}{m\,\epsilon }\right) + N_r(\tau )\right) . \end{aligned}$$

Here, the estimate in 1 comes from convexity (B3), while that in 2 is an application of the subadditivity of $N_r$ (Lemma 28) together with Proposition 30. On the other hand, we can write

$$\begin{aligned} N_r(\omega \otimes \tau )&= N_r\left( \frac{1}{1+\epsilon }\, \rho \otimes \tau + \frac{\epsilon }{1+\epsilon }\, \delta ' \otimes \tau \right) \\&{\mathop {\ge }\limits ^{{\text{3 }}}} \frac{1}{1+\epsilon }\, N_r(\rho \otimes \tau ) + \frac{\epsilon }{1+\epsilon }\, N_r(\delta ' \otimes \tau ) - h_2\left( \frac{\epsilon }{1+\epsilon } \right) \\&{\mathop {\ge }\limits ^{{\text{4 }}}} \frac{1}{1+\epsilon }\, N_r(\rho \otimes \tau ) + \frac{\epsilon }{1+\epsilon }\, N_r(\tau ) - h_2\left( \frac{\epsilon }{1+\epsilon } \right) , \end{aligned}$$

where the inequality in 3 is the lower bound in (B3), and that in 4 holds because of the monotonicity of $N_r$ under the classical operation of tracing away subsystems. Putting all together we see that

$$\begin{aligned} N_r(\rho \otimes \tau ) - N_r(\sigma \otimes \tau ) \le m\, \epsilon \, g\left( \frac{E}{m\, \epsilon } \right) + (1+\epsilon ) h_2\left( \frac{\epsilon }{1+\epsilon } \right) = m\, \epsilon \, g\left( \frac{E}{m\, \epsilon } \right) + g(\epsilon )\, . \end{aligned}$$

Together with the corresponding inequality with $\rho$ and $\sigma$ exchanged, this yields (B2), and in particular proves (B1) for $F=N_r$.

Now, again borrowing a telescopic argument from [131], for all $n\in \mathbb {N}_+$ we have that

$$\begin{aligned} \left| N_r( \rho ^{\otimes n}) - N_r(\sigma ^{\otimes n}) \right|&= \left| \sum \nolimits _{k=0}^{n-1} \left( N_r\left( \rho ^{\otimes (n-k)}\otimes \sigma ^{\otimes k}\right) - N_r\left( \rho ^{\otimes (n-k-1)}\otimes \sigma ^{\otimes (k+1)}\right) \right) \right| \\&\le \sum \nolimits _{k=0}^{n-1} \left| N_r\left( \rho ^{\otimes (n-k)}\otimes \sigma ^{\otimes k}\right) - N_r\left( \rho ^{\otimes (n-k-1)}\otimes \sigma ^{\otimes (k+1)}\right) \right| \\&= \sum \nolimits _{k=0}^{n-1} \left| N_r\left( \rho \otimes \tau _k \right) - N_r\left( \sigma \otimes \tau _k\right) \right| \\&\le k\, \left( m\, \epsilon \, g\left( \frac{E}{m\,\epsilon } \right) + g(\epsilon ) \right) , \end{aligned}$$

where in the last line we applied (B2). Diving by *k* and taking the limit for $k\rightarrow \infty$ we see that

$$\begin{aligned} \left| N_r^\infty (\rho ) - N_r^\infty (\sigma ) \right| \le m\, \epsilon \, g\left( \frac{E}{m\,\epsilon } \right) + g(\epsilon )\, , \end{aligned}$$

which proves (B1) also when $F=N_r^\infty$.

The case of $F=N^M_r\!$ can be tackled with exactly the same techniques, because $N^M_r\!$ obeys an inequality analogous to (B3). In turn, this is a consequence of the fact that the classical Kullback–Leibler divergence satisfies the estimates in [45, Proposition 5.24]. $\square$

### Remark B57

We have not been able to establish (B1) also for the remaining case of $F=N^{M,\infty }_r\!$, essentially because we lack a statement similar to (B2) for $N^M_r\!$. In turn, this is due to the fact that this latter quantity is not subadditive — in fact, it is strongly superadditive!

The application of Lemma B55 that is of interest to us is as follows.

### Corollary B58

Let $\rho ,\sigma \in \mathcal {D}(\mathcal {H}_{m})$ be two *m*-mode states, and set $\epsilon :=\frac{1}{2} \left\| \rho -\sigma \right\| _1$. Assume that ${{\,\textrm{Tr}\,}}\left[ \rho \left( \sum \nolimits _{j=1}^m a_j^\dag a_j\right) \right] \le E$ and also ${{\,\textrm{Tr}\,}}\left[ \sigma \left( \sum \nolimits _{j=1}^m a_j^\dag a_j\right) \right] \le E$. Then, for $F=N^M_r\!, N_r^\infty , N_r$ it holds that

B4 $$\begin{aligned} \left| F(\rho ) - F(\sigma ) \right| \le m\, \epsilon \, g\left( \frac{2E}{m\,\epsilon } \right) + g(\epsilon )\, , \end{aligned}$$

where again $g(x) = (1+x) \log _2(1+x) - x \log _2 x$. In particular, denoting with $\rho =\sum _k p_k \vert e_k\rangle \!\langle e_k\vert$ the spectral decomposition of $\rho$, the sequence of spectral truncations $\rho _n:=\left( \sum \nolimits _{k\le n} p_k\right) ^{-1} \sum _{k\le n} p_k \vert e_k\rangle \!\langle e_k\vert$ satisfies that

B5 $$\begin{aligned} F(\rho ) = \lim _{n\rightarrow \infty } F(\rho _n)\, . \end{aligned}$$

### Proof

Thanks to Lemma B55, in order to prove (B4) it suffices to show that ${{\,\textrm{Tr}\,}}\left[ |\rho -\sigma | \left( \sum \nolimits _{j=1}^m a_j^\dag a_j\right) \right] \le 2E$. Indeed, if $\rho =\sum _k p_k \vert e_k\rangle \!\langle e_k\vert$ and $\sigma =\sum _k q_k \vert e_k\rangle \!\langle e_k\vert$ then

$$\begin{aligned} |\rho -\sigma | = \sum _k |p_k-q_k| \vert e_k\rangle \!\langle e_k\vert \le \sum _k (p_k+q_k) \vert e_k\rangle \!\langle e_k\vert =\rho +\sigma \, , \end{aligned}$$

so that

$$\begin{aligned} {{\,\textrm{Tr}\,}}\left[ |\rho -\sigma | \left( \sum \nolimits _{j=1}^m a_j^\dag a_j\right) \right] \le {{\,\textrm{Tr}\,}}\left[ (\rho +\sigma ) \left( \sum \nolimits _{j=1}^m a_j^\dag a_j\right) \right] \le 2E\, . \end{aligned}$$

To deduce (B5), note that $[\rho ,\rho _n]=0$, with . Also, for sufficiently large *n* the mean photon number of $\rho _n$ is at most twice that of $\rho$ (call it *E*), so that

where we used the well-known fact that $\lim _{\epsilon \rightarrow 0^+} \epsilon \, g(\delta /\epsilon )=0$ for all $\delta >0$. $\square$

## Appendix C: A Technical Lemma

The following result was invoked in the proof of Proposition 40 (more precisely, in step 3 of (66)) as an alternative to [45, Proposition 5.23(iv)]. For the sake of completeness, we include a proof.

### Lemma B59

Let us consider two *m*-mode states $\rho ,\sigma$, with $S_W(\rho )<\infty$. Then, it holds that

$$\begin{aligned} D^M\!(\rho \Vert \sigma )\ge D_{K\! L}\!(Q_\rho \Vert Q_\omega )\,. \end{aligned}$$

### Proof

At fist sight, the result might seem a trivial application of the definitions of measured relative entropy as given in (10) and of heterodyne detection as given in [126, 5.4.2]. The only obstacle is that, according to our definition, a POVM must be a finite collection of operators; this is also crucial for proving Lemma 20, and hence cannot be simply removed. The idea of the proof is precisely to show that an heterodyne detection, which corresponds to the decomposition of the identity $\mathbb {1}= \int _{\mathbb {C}^m} \frac{d^{2m}\alpha }{\pi ^m} \, \vert \alpha \rangle \!\langle \alpha \vert$, can be approximated, inside the Kullback–Leibler divergence, by a sequence of finite POVMs. Note that the hypothesis $S_W(\rho )<\infty$ is needed to ensure that $D_{K\! L}\!(\rho \Vert \sigma )$ is a well-defined Lebesgue integral for any $\sigma \in \mathcal {D}(\mathcal {H}_{})$ (possibly diverging to $+\infty$).

Let us fix $\epsilon ,\,\eta ,\,r_1,\,r_2,\,\ell >0$ and consider the family of subsets of $\mathbb {C}^m$, $\mathcal {A}=\{A_n\}_{n=0,\ldots ,N+1}$, with *N* a finite positive integer, constructed as follows:

- $$A_0:=\left\{ \alpha \in \mathbb {C}^m:Q_\rho (\alpha )<\eta \text { or }Q_\sigma (\alpha )<\eta \right\} \,.$$
- For $n=1,\ldots ,N$, $A_n$ is a subset of $\mathbb {C}^m$ such that:
  - It is contained in a ball of radius $\ell$;
  - $$A_n\cap A_{n'}=\emptyset $$ if $$n\ne n'$$;
  - $B_m(r_1)\subset \bigcup _{n=0}^{N}A_n$ and $\bigcup _{n=1}^{N}A_n\subset B_m(r_2)$, where $B_m(r)$ is the *m*-dimensional ball with radius *r* and centered at the origin of $\mathbb {C}^m$.
- $$A_{N+1}:=\mathbb {C}^m\setminus \bigcup _{n=0}^{N}A_n$$.

Notice that we do not specify the dependence of $\mathcal {A}$ upon $\epsilon ,\,\eta ,\,r_1,\,r_2$ and $\ell$ for ease of notation. It is clear from the definition that the set $\mathcal {M}=\{E_n\}_{n=1,\ldots ,N}$, with

$$\begin{aligned} E_n= \int _{A_n} \frac{d^{2m}\alpha }{\pi ^m}\, \vert \alpha \rangle \!\langle \alpha \vert \end{aligned}$$

is a proper, finite POVM.

For a fixed state $\rho$ we can compute the classical probability distribution

$$\begin{aligned} \mathcal {P}^{\mathcal {M}}_\rho (n)={{\,\textrm{Tr}\,}}[\rho E_n]= \int _{A_n} \frac{d^{2m}\alpha }{\pi ^m} \,\langle \alpha |\rho |\alpha \rangle =\int _{A_n} \frac{d^{2m}\alpha }{\pi ^m} \,Q_\rho (\alpha )\,. \end{aligned}$$

Since $Q_\rho \in L^1(\mathbb {C}^m)$, we can always take $r_1$ big enough so that $\mathcal {P}^{\mathcal {M}}_\rho (N+1)<\epsilon$. Similarly we can always choose $\eta$ such that $\mathcal {P}^{\mathcal {M}}_\rho (0)<\epsilon$. Now, given another fixed state $\sigma$, we have:

Here, in 1 we have discarded two positive terms, in 2 we have used the fact that $\mathcal {P}_\rho ^{\mathcal {M}}(0),\mathcal {P}_\rho ^{\mathcal {M}}(N+1)<\epsilon$ and $\lim _{x\rightarrow 0}x\log _2x=0$, in 3 we have used the fact that $Q_\rho$ and $Q_\sigma$ are uniformly continuous on any compact set and finally in 4 we have used the fact that also the logarithm is uniformly continuous on $\bigcup _{n=1}^NA_n$. Now, by taking the proper limits, i.e., $\epsilon ,\eta ,\ell \rightarrow 0$ and $r_1,r_2\rightarrow \infty$, we obtain precisely the generalized Riemann integral over $\mathbb {C}^m$ of the function $Q_\rho (\alpha )\left( \log _2Q_\rho (\alpha )-\log _2Q_\sigma (\alpha )\right)$. Since $S_W(\rho )<\infty$, the function is absolutely integrable, and the generalized Riemann integral is well defined and coincides with the Lebesgue one. $\square$

## Footnotes

1 A net $(x_\alpha )_\alpha$ on some set $\mathcal {X}$ is any function of the form $x:A\rightarrow \mathcal {X}$, where *A* is a directed set, i.e., a set *A* equipped with a preorder $\le$ such that for all $a,b\in A$ there exists $c\in A$ with the property that $a\le c$ and $b\le c$.

2 Here, classical registers are thought of as special *d*-dimensional quantum systems *X* ($d<\infty$) with the property that any free operation $\Lambda \in {\mathscr {F}}(A\rightarrow XB)$ satisfies that $(\Delta _X\otimes I_B)\circ \Lambda =\Lambda$, with $\Delta _X(\cdot ):=\sum _{i=1}^{d} \vert i\rangle \!\langle i\vert (\cdot )\vert i\rangle \!\langle i\vert$ being the dephasing map. In other words, if a quantum system plays the role of a classical register, then no state apart from those that are diagonal in a fixed orthonormal basis $\{\vert i\rangle \}_{i=1,\ldots , d}$ is ever accessible with free operations.

3 Notable exceptions are monotones based on the partial transpose in entanglement theory [81–83].

4 LL acknowledges useful discussions with Andreas Winter and Krishna Kumar Sabapathy on the problem of calculating $N_r(\vert n\rangle \!\langle n\vert )$.
